# Oxidation of C–H and O–H bonds by a copper complex inspired by the Cu(ii)–tyrosyl species formed in LPMOs

**DOI:** 10.1039/d5sc07166f

**Published:** 2025-10-17

**Authors:** David D. Hebert, Daniel Ye, Isaac Garcia-Bosch

**Affiliations:** a Department of Chemistry, Carnegie Mellon University Pittsburgh Pennsylvania 15213 USA igarciab@andrew.cmu.edu

## Abstract

Cupric tyrosyl intermediates have been invoked as active oxidants in oxidase and oxygenase Cu-dependent metalloenzymes. Inspired by these natural oxidants, we report the proton-coupled electron transfer (PCET) reactivity of Cu complexes bound by a tridentate redox-active ONO pincer ligand and an ancillary amine ligand, [LCu(A)]^*n*+^ (L = bis(3,5-di-*tert*-butyl-2-hydroxyphenyl)amine; A = triethylamine (NEt_3_) or *N*,*N*,*N*′,*N*′-tetramethylpropane-1,3-diamine (tmpda); *n* = 0, 1). Analysis of the stoichiometry of the reactions indicated that the iminosemiquinone complex [^sq^LCu(NEt_3_)] acts as 1H^+^/1e^−^ PCET acceptor, while the benzoquinone analogue [^bq^LCu(NEt_3_)]^+^ reacts in a 2H^+^/2e^−^ fashion. Thermochemical analysis of the PCET reactivity of [^sq^LCu(NEt_3_)] and [^bq^LCu(NEt_3_)]^+^ revealed that [^bq^LCu(NEt_3_)]^+^ is a stronger H-atom acceptor, which led to faster PCET reactions. [^bq^LCu(NEt_3_)]^+^ reacted with substrates containing weak O–H bonds and, to our surprise, also abstracted H-atoms from C–H substrates. The reactivity of [^bq^LCu(NEt_3_)]^+^ was compared with other Cu complexes developed in our laboratory that are stronger H-atom acceptors but do not oxidize C–H substrates, suggesting that non-thermodynamic factors contribute to the enhanced reactivity of [^bq^LCu(NEt_3_)]^+^ towards C–H bonds. This work describes the first example of Cu complex bound by a redox-active ligand able to oxidize C–H bonds, and provides evidence of the involvement of similar species in the oxidation of organic substrates catalyzed by Cu-dependent metalloenzymes such as lytic polysaccharide monooxygenases.

## Abstract 2

This study examines the stoichiometry, thermochemistry, kinetics, and mechanism of the reactions of mononuclear Cu complexes bearing redox-active ligands with organic substrates containing C–H and O–H bonds.

## Graphical abstract

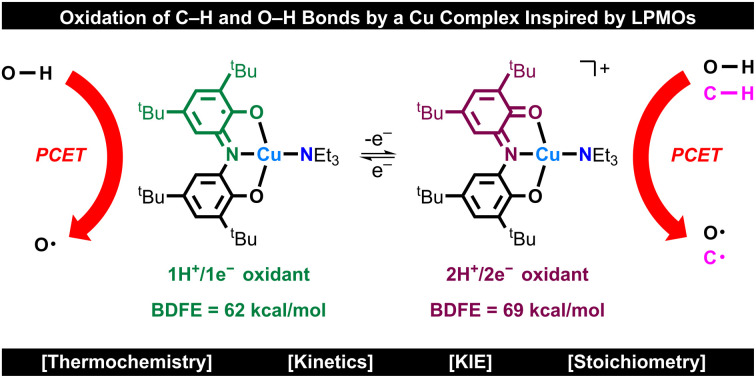

## Introduction

Lytic polysaccharide monooxygenase enzymes (LPMOs) are Cu-dependent enzymes that catalyze the oxidative degradation of recalcitrant polysaccharides such as cellulose and chitin.^1–3^ Degradation is initiated by the hydroxylation of strong C–H bonds in the polysaccharide substrate (bond dissociation free energy, BDFE ∼100 kcal mol^−1^), a reaction that leads to cleavage of the glycosidic bond.^4^ Despite intense study, the precise mechanism(s) by which LPMOs activate such inert bonds remains under debate. Early mechanistic proposals suggested that LPMOs function as monooxygenases utilizing O_2_ as the oxidant.^5^ However, recent studies support a peroxygenase mechanism in which H_2_O_2_ serves as the active oxidant.^6,7^

In addition to their ability to oxidize C–H bonds, LPMOs have also been proposed to perform oxidase-like chemistry by reducing O_2_ to H_2_O_2_, which can then be used for peroxygenase chemistry, or can be further reduced to water (*i.e.*, peroxidase chemistry).^8,9^ Most mechanistic proposals involve reduction of the active-site Cu center to the cuprous state upon substrate binding, enabling subsequent activation of O_2_ or H_2_O_2_ to form a putative Cu^II^–oxyl species which carries out C–H hydroxylation of the substrate.^10,11^ Alternative proposals include the involvement of a Cu^III^OH species, formed *via* deprotonation of the terminal NH_2_ group of the histidine brace bound to the Cu center, or a Cu^II^-tyrosyl-hydroxo species generated *via* 1H^+^/1e^−^ oxidation of the axial tyrosine residue found in most LMPOs.^11^

**Fig. 1:**
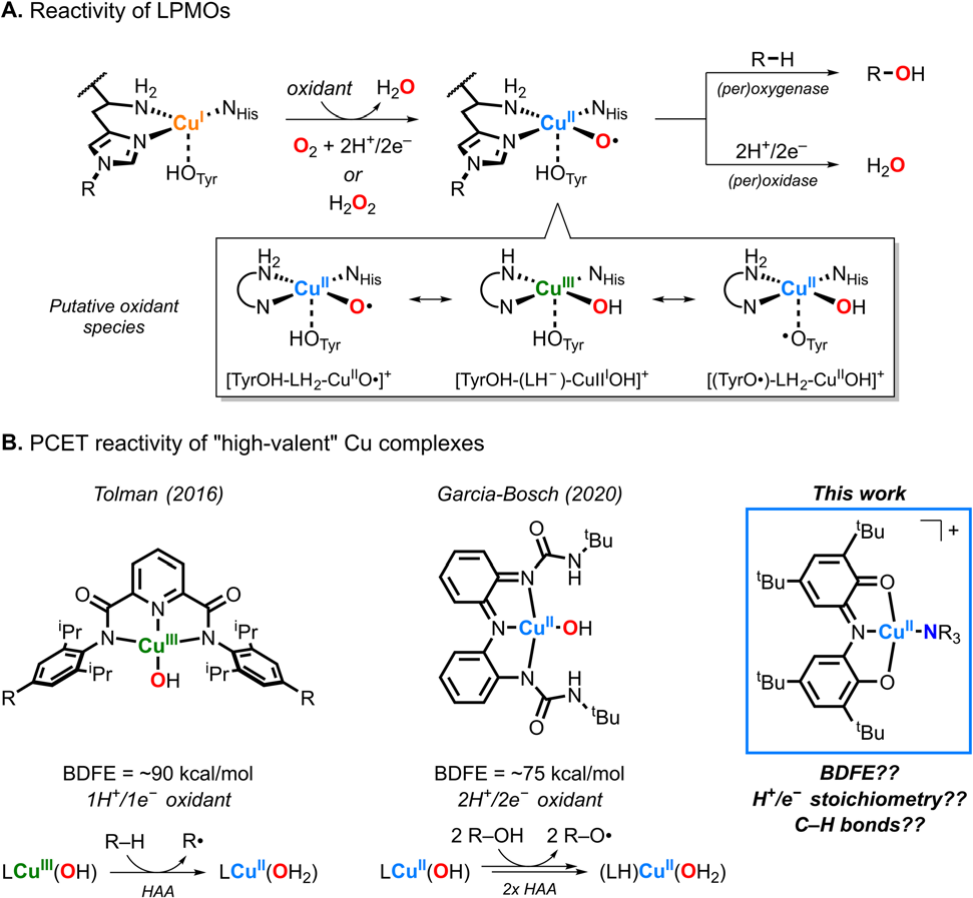
(A) Oxidation of C–H bonds (monooxygenase, peroxygenase) and reduction of O_2_/H_2_O_2_ (oxidase, peroxidase) by LPMOs. (B) PCET reactivity of previously reported “high-valent” Cu complexes and the Cu–ONO complexes studied in this work.

**Scheme 1:**
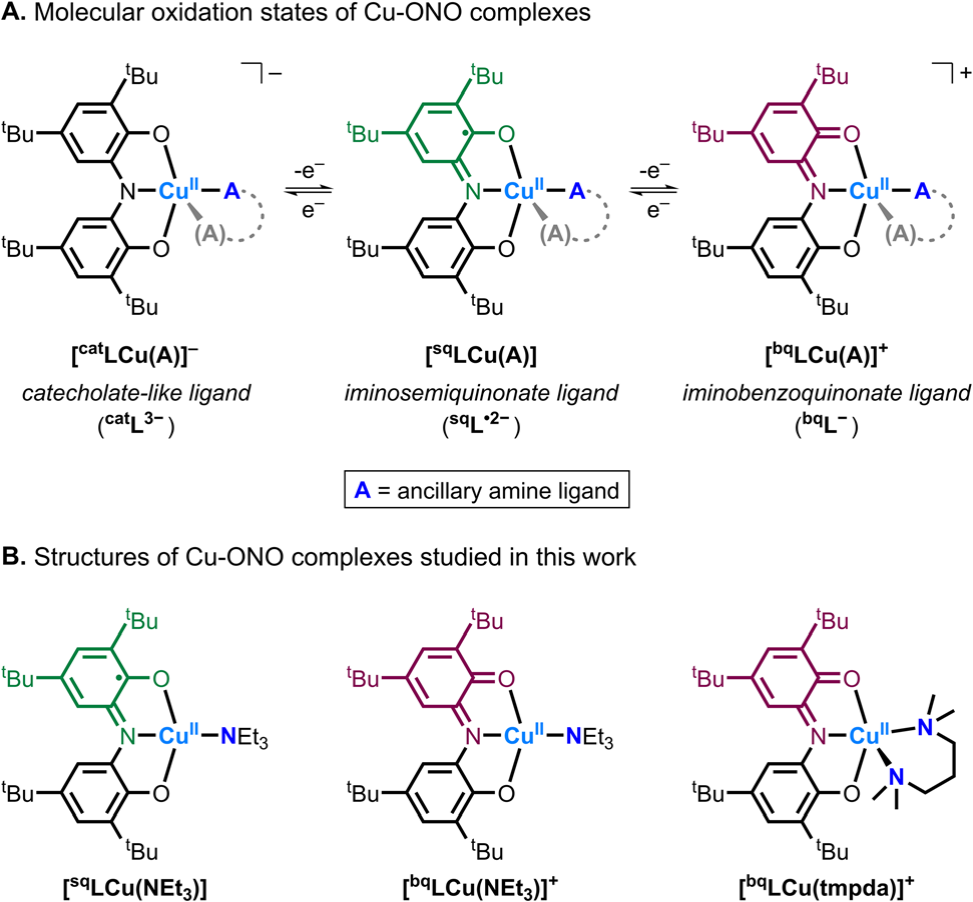
(A) Molecular oxidation states of the Cu–ONO complexes. (B) Structures of the Cu–ONO complexes studied in this work.

In a landmark contribution, Tolman and coworkers described the synthesis and characterization of a mononuclear Cu^III^OH complex capable of oxidizing strong C–H bonds (Fig. 1B).^12^ The reaction was proposed to proceed *via* hydrogen atom transfer (HAT) from the substrate to the Cu^III^OH core, yielding a Cu^II^-aqua species and a carbon-centered radical. While the Cu^III^OH complex exhibited 1H^+^/1e^−^ oxidase-like reactivity, substrate hydroxylation was not observed. Building on this precedent, our laboratory reported the synthesis and characterization of a Cu^II^OH complex supported by a redox-active ligand capable of accessing three discrete molecular oxidation states, modeling the proposed Cu^II^-tyrosyl-hydroxo species in LPMOs.^13^ Our studies showed that the “high-valent” Cu^II^OH species, bound by the oxidized iminobenzoquinonate form of the ligand, acted as a 2H^+^/2e^−^ oxidant but was limited to substrates with weak O–H bonds (Fig. 1B and Scheme 1).

More recently, we reported the synthesis and characterization of a family of mononuclear Cu^II^ complexes supported by a redox-active ONO ligand and ancillary amine ligands (*e.g.*, triethylamine, *N*,*N*,*N*′,*N*′-tetramethylpropane-1,3-diamine; Scheme 1).^14^ The Cu–ONO complexes similarly accessed three molecular oxidation states *via* ligand-center redox processes. In this article, we investigate the reactivity of selected Cu–ONO species towards C–H and O–H bond substrates, providing new insight into Cu–ligand redox cooperativity and proton-coupled electron transfer (PCET) reactivity in bioinspired copper complexes (Fig. 1B).

## Results and discussion

### PCET reactivity: scope and stoichiometry

**Scheme 2:**
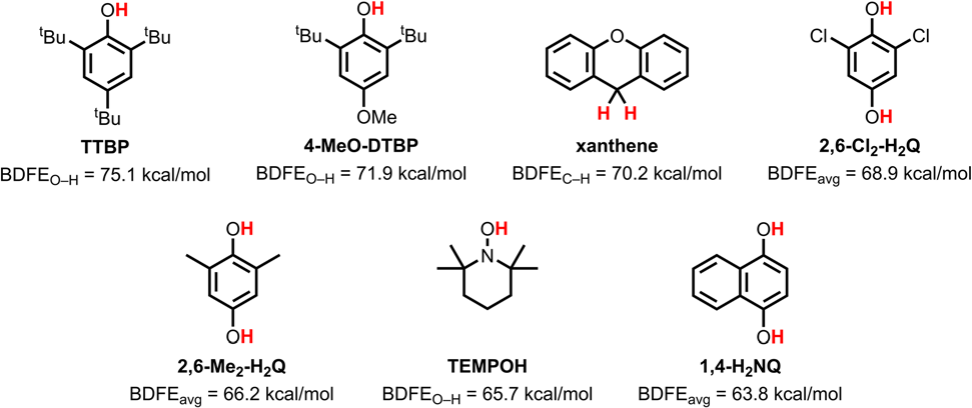
Structures and bond dissociation free energies (BDFEs) of the PCET substrates used in this work.

The PCET reactivity of the Cu–ONO complexes [^sq^LCu(NEt_3_)] and [^bq^LCu(NEt_3_)]^+^ (Scheme 1) was investigated with substrates containing weak O–H bonds, including 2,2,6,6-tetramethylpiperidin-1-ol (TEMPOH), hydroquinones, and phenols (Scheme 2; see the SI). A summary of the observed reactivity is provided in Table 1. Unless otherwise noted, the bond dissociation free energy (BDFE) values for the substrates discussed in this and subsequent sections have been determined experimentally and have an uncertainty of ±1 kcal mol^−1^.^15,16^

**Table 1:** Summary of the qualitative reactivity of [^sq^LCu(NEt_3_)] and [^bq^LCu(NEt_3_)]^+^ with PCET reagents monitored by UV-vis spectroscopy

Substrate	BDFE_O–H_, kcal mol^−1^	Complex	
		[^sq^LCu(NEt_3_)]	[^bq^LCu(NEt_3_)]^+^
TTBP	75.1	n.r.	n.r.
4-MeO-DTBP	71.9	n.r.	Equilibrium
Xanthene	70.2 (DMSO)	n.r.	Reaction
2,6-Cl_2_-H_2_Q	68.9	Equilibrium	Reaction
2,6-Me_2_-H_2_Q	66.2	Equilibrium	Reaction
TEMPOH	65.7	Equilibrium	Reaction
1,4-H_2_NQ	63.8	Reaction	Reaction

Complex [^sq^LCu(NEt_3_)] displayed modest reactivity towards PCET reagents. Complete decay of the characteristic UV-vis absorption features was observed only with 1,4-dihydroxynapthalene (1,4-H_2_NQ), the weakest O–H bond substrate tested (BDFE_avg_ = 63.8 kcal mol^−1^), consistent with reduction of the iminosemiquinonate ligand (^sq^L˙^2−^) to the catecholate-like form (^cat^L^3−^). Partial reactivity, indicative of an equilibrium, was observed with TEMPOH (BDFE_O–H_ = 65.7 kcal mol^−1^), 2,6-dimethyl-1,4-hydroquinone (2,6-Me_2_-H_2_Q; BDFE_avg_ = 66.2 kcal mol^−1^), and 2,6-dichloro-1,4-hydroquinone (2,6-Cl_2_-H_2_Q; BDFE_avg_ = 68.9 kcal mol^−1^). No reaction was observed with 2,6-di-*tert*-butyl-4-methoxyphenol (4-MeO-DTBP; BDFE_O–H_ = 71.9 kcal mol^−1^) or 2,4,6-tri-*tert*-butylphenol (TTBP; BDFE_O–H_ = 75.1 kcal mol^−1^), the strongest O–H bond substrates examined. In contrast, the oxidized complex [^bq^LCu(NEt_3_)]^+^ (generated *via* 1e^−^ oxidation of [^sq^LCu(NEt_3_)] with ferrocenium hexafluorophosphate, FcPF_6_) exhibited greater PCET reactivity. [^bq^LCu(NEt_3_)]^+^ reacted with all O–H bond substrates tested but reached equilibrium with 4-MeO-DTBP and showed no reaction with TTBP.

**Fig. 2:**
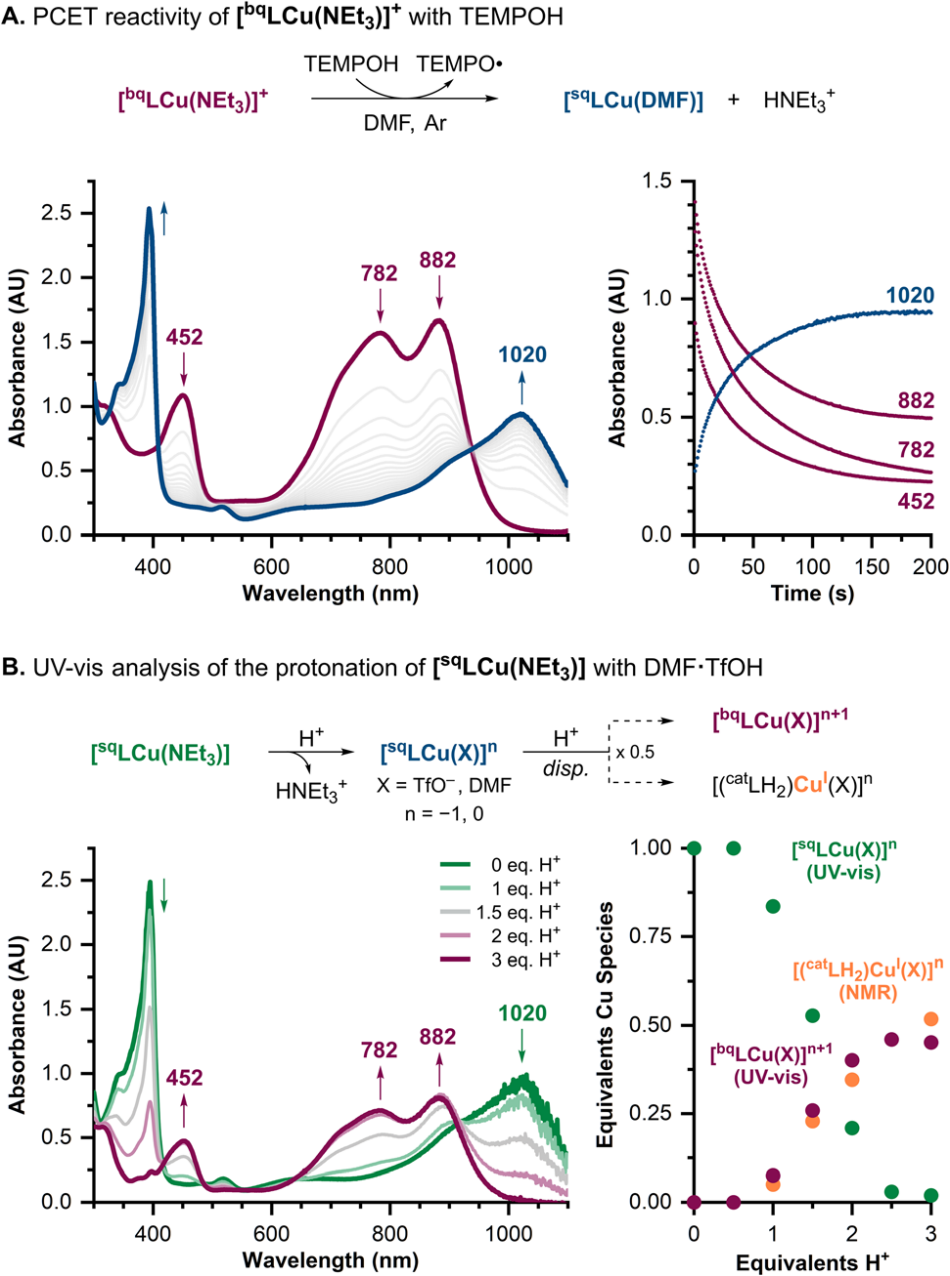
(A) Scheme for the reaction of [^bq^LCu(NEt_3_)]^+^ with TEMPOH, the UV-vis spectra of the reaction, and time course of the absorption bands at 452, 782, 882, and 1020 nm ([Cu] = 0.125 mM, [TEMPOH] = 4.69 mM, 37.5 equiv.). (B) Scheme for the protonation of [^sq^LCu(NEt_3_)] with DMF·TfOH, UV-vis spectra of the titration, and equivalents of the Cu species formed with varying amounts of acid.

The stoichiometry of the PCET reactions was investigated by UV-vis, NMR, and EPR spectroscopy. Reaction of [^bq^LCu(NEt_3_)]^+^ (0.125 mM) with 37.5 equiv. of TEMPOH at room temperature under an argon atmosphere led to decay of the absorption features at 452, 782, and 882 nm, yielding spectra closely resembling those of [^sq^LCu(NEt_3_)], which was recovered in >90% spectroscopic yield (Fig. 2A). These spectral changes are consistent with reduction of the ^bq^L^−^ ligand to ^sq^L˙^2−^. We propose that the product Cu complex is a Cu^II^-iminosemiquinone species in which the ancillary amine ligand has been replaced by a coordinating solvent molecule (*e.g.*, [^sq^LCu(DMF)]; see further details below). EPR analysis of the reaction between [^bq^LCu(NEt_3_)]^+^ and TEMPOH showed formation of 1.5 equiv. of TEMPO radical (see SI). The higher-than-expected yield of TEMPO radical is attributed to the partial reaction of the resulting [^sq^LCu(DMF)] complex with TEMPOH. This was corroborated by EPR analysis of the reaction between [^sq^LCu(NEt_3_)] and TEMPOH, which showed the formation of 0.5 equiv. TEMPO radical (see SI).

Protonation experiments further supported the PCET stoichiometry. The titration of [^sq^LCu(NEt_3_)] with *N*,*N*-dimethylformamidinium triflate (DMF·TfOH)^17^ was monitored by UV-vis and NMR spectroscopy (see Fig. 2B and SI). Addition of 1 equiv. of acid did not lead to significant changes in the UV-vis spectrum. However, NMR analysis showed quantitative formation of HNEt_3_^+^ after addition of 1 equiv. acid, consistent with protonation and displacement of the ancillary NEt_3_ ligand (likely by triflate or solvent). Further addition of acid induced disproportionation to yield 0.5 equiv. of [^bq^LCu(X)]^*n*+1^ (where X = TfO^−^ or DMF and *n* = −1 or 0, respectively) by UV-vis, and 0.5 equiv. of a Cu^I^–ONO species ([(^cat^LH_2_)Cu^I^(X)]^*n*^) detected by NMR (see SI).

Titration of the reduced complex [^cat^LCu(NEt_3_)]^−^ (generated *via* 1e^−^ reduction of [^sq^LCu(NEt_3_)] with cobaltocene, CoCp_2_) with DMF·TfOH was monitored by UV-vis spectroscopy. Addition of 1 equiv. acid resulted in disproportionation, yielding 0.5 equiv. of a species resembling [^sq^LCu(NEt_3_)] (see SI).

**Fig. 3:**
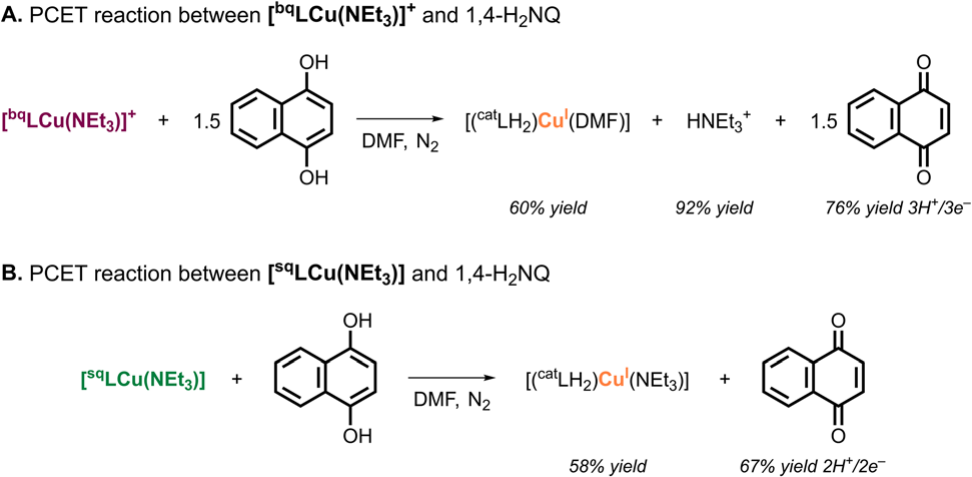
Stoichiometry for the PCET reaction between [^bq^LCu(NEt_3_)]^+^ and 1,4-H_2_NQ (A), and for [^sq^LCu(NEt_3_)] with 1,4-H_2_NQ (B). Note: yields were spectroscopically determined by ^1^H NMR and represent the average of duplicate experiments (see SI).

The stoichiometry of PCET reactions with 1,4-H_2_NQ was analyzed by ^1^H NMR (Fig. 3). The reaction of [^bq^LCu(NEt_3_)]^+^ with 1,4-H_2_NQ (5 equiv.) produced 1.14 equiv. of naphthalene-1,4-dione (1,4-NQ), indicating 3H^+^/3e^−^ PCET stoichiometry (76% yield; see SI). Consistent with the protonation studies, 0.5 equiv. of Cu^I^–ONO species and 1 equiv. of HNEt_3_^+^ were detected. In contrast, the reaction of [^sq^LCu(NEt_3_)] with 5 equiv. 1,4-H_2_NQ afforded 0.67 equiv. 1,4-NQ, corresponding to 67% yield based on 2H^+^/2e^−^ stoichiometry (see SI). The NMR spectra also indicated formation of 0.5 equiv. Cu^I^–ONO species, however, no HNEt_3_^+^ was observed.

**Fig. 4:**
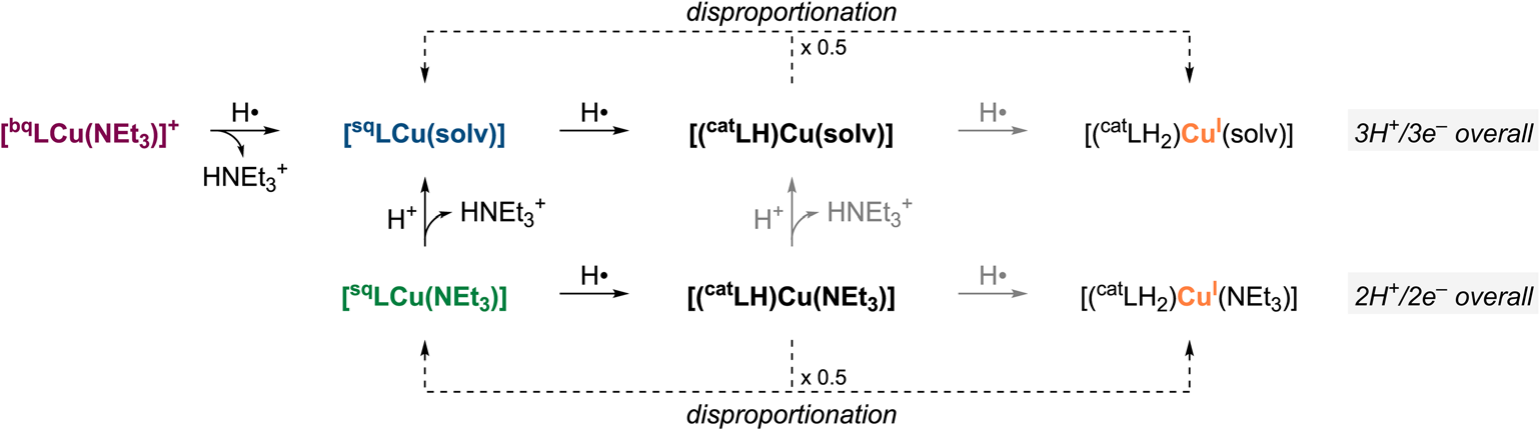
Overall PCET stoichiometry scheme.

The overall PCET stoichiometry is summarized in Fig. 4. Our data indicate that [^bq^LCu(NEt_3_)]^+^ formally undergoes 2H^+^/2e^−^ reductive protonation (*i.e.*, accepts two H-atom equivalents), but subsequent disproportionation yields an overall 3H^+^/3e^−^ stoichiometry. The first PCET event is proposed to reduce the ONO ligand from ^bq^L^−^ to ^sq^L˙^2−^, accompanied by protonation of the ancillary NEt_3_ ligand which is displaced by the solvent to produce [^sq^LCu(solv)]. This is supported by the formation of HNEt_3_^+^ in the reaction of [^bq^LCu(NEt_3_)]^+^ with 1,4-H_2_NQ (by NMR) and a Cu^II^-iminosemiquinone species in the reaction of [^bq^LCu(NEt_3_)]^+^ with TEMPOH (by UV-vis). Our proposal is also consistent with the results observed in the protonation of [^sq^LCu(NEt_3_)], in which the addition of 1 equiv. of acid resulted in only minor changes of the spectra (*i.e.*, the UV-vis of [^sq^LCu(NEt_3_)] and [^sq^LCu(solv)] are almost identical). The second PCET event (1H^+^/1e^−^ reduction of [^sq^LCu(solv)]) is proposed to be a ligand-based reductive protonation of ^sq^L˙^2−^ to ^cat^LH^2−^, triggering disproportionation of the resulting cupric catecholate-like species to form 0.5 equiv. of a cuprous catecholate-like species (characterized and quantified by NMR) and regenerate 0.5 equiv. of [^sq^LCu(solv)], leading to an overall 3H^+^/3e^−^ stoichiometry.

Similarly, [^sq^LCu(NEt_3_)] acts as a formal 1H^+^/1e^−^ acceptor, but disproportionation reactions triggered by its reductive protonation give a net 2H^+^/2e^−^ stoichiometry. The PCET event is proposed to be ligand-centered reductive protonation of ^sq^L˙^2−^ to ^cat^LH^2−^, followed by disproportionation to generate 0.5 equiv. of [^sq^LCu(NEt_3_)] and 0.5 equiv. of a Cu^I^–ONO species.

### Thermochemical analysis

**Fig. 5:**
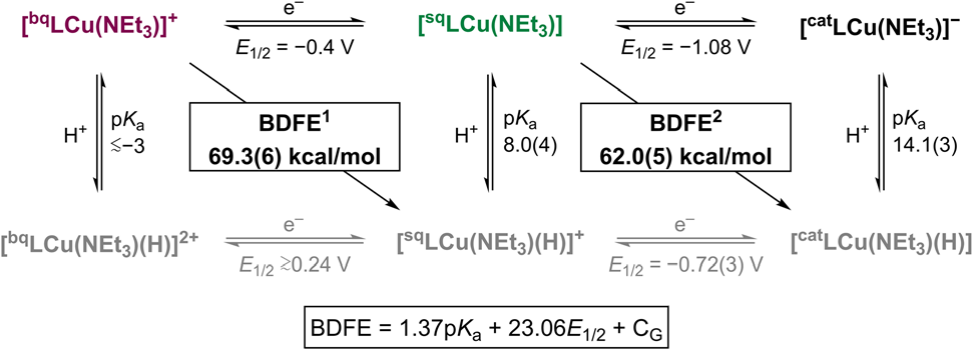
Square-scheme summarizing the thermodynamics of the Cu–ONO species involved in PCET. The Bordwell equation is shown at the bottom (*C*_G_ = 67.6 kcal mol^−1^ in DMF).^15^

The BDFEs of [^sq^LCu(NEt_3_)] and its oxidized form [^bq^LCu(NEt_3_)]^+^ were estimated *via* equilibrium studies with reference substrates of known BDFE_O–H_ values (see SI). The equilibria between [^sq^LCu(NEt_3_)] and TEMPOH (BDFE_O–H_ 65.7 kcal mol^−1^), and between [^bq^LCu(NEt_3_)]^+^ with 4-MeO-DTBP (BDFE_O–H_ 71.9 kcal mol^−1^), were monitored across varying substrate concentrations. Based on the equilibrium positions, the BDFE of [^sq^LCu(NEt_3_)] was estimated to be 62.0 ± 0.5 kcal mol^−1^, while that of [^bq^LCu(NEt_3_)]^+^ was estimated to be 69.3 ± 0.6 kcal mol^−1^ (Fig. 5; see SI).

From the BDFE values and redox potentials of [^sq^LCu(NEt_3_)] (*E*_1/2_ = −1.08 V *vs.* Fc^+/0^) and [^bq^LCu(NEt_3_)]^+^ (*E*_1/2_ = −0.4 V *vs.* Fc^+/0^),^14^ the Bordwell equation (Fig. 5) was used to calculate the corresponding p*K*_a_ values of [^sq^LCu(NEt_3_)] and [^cat^LCu(NEt_3_)]^−^. The p*K*_a_ values for protonation of [^sq^LCu(NEt_3_)] and [^cat^LCu(NEt_3_)]^−^ were calculated to be 8.0 ± 0.4, and 14.1 ± 0.3, respectively.

As discussed above, the UV-vis spectrum of [^sq^LCu(NEt_3_)] did not significantly change upon addition of 1 equiv. of acid, which precluded accurate p*K*_a_ determination by direct titration. However, the observed formation of HNEt_3_^+^ (p*K*_a_ 9.2 in DMF)^18^ upon protonation with 1 equiv. of DMF·TfOH suggests the p*K*_a_ of [^sq^LCu(NEt_3_)] is less than 9. Similarly, experimental determination of the p*K*_a_ of [^cat^LCu(NEt_3_)]^−^ was hindered by disproportionation of the protonated complex. Therefore, the p*K*_a_ of [^cat^LCu(NEt_3_)]^−^ was approximated through qualitative protonation experiments using phenol derivatives of varying acidity (see SI). Disproportionation was observed upon addition of excess 3-nitrophenol (p*K*_a_ = 14.6 in DMF) and 4-chlorophenol (p*K*_a_ = 16.8 in DMF), whereas no reaction occurred with 4-fluorophenol (p*K*_a_ = 18.8 in DMF).^18^ These observations suggest that the p*K*_a_ of [^cat^LCu(NEt_3_)]^−^ lies between 13 and 16, consistent with the calculated p*K*_a_ of 14 (see SI). The p*K*_a_ of [^bq^LCu(NEt_3_)]^+^ was estimated by titration with DMF·TfOH. Titration with 10 equiv. acid resulted in the gradual decay of the complex, as observed by UV-vis spectroscopy. Based on these results, we tentatively assign the p*K*_a_ of [^bq^LCu(NEt_3_)]^+^ to be approximately ≲−3 (see SI).

### Kinetic studies

**Fig. 6:**
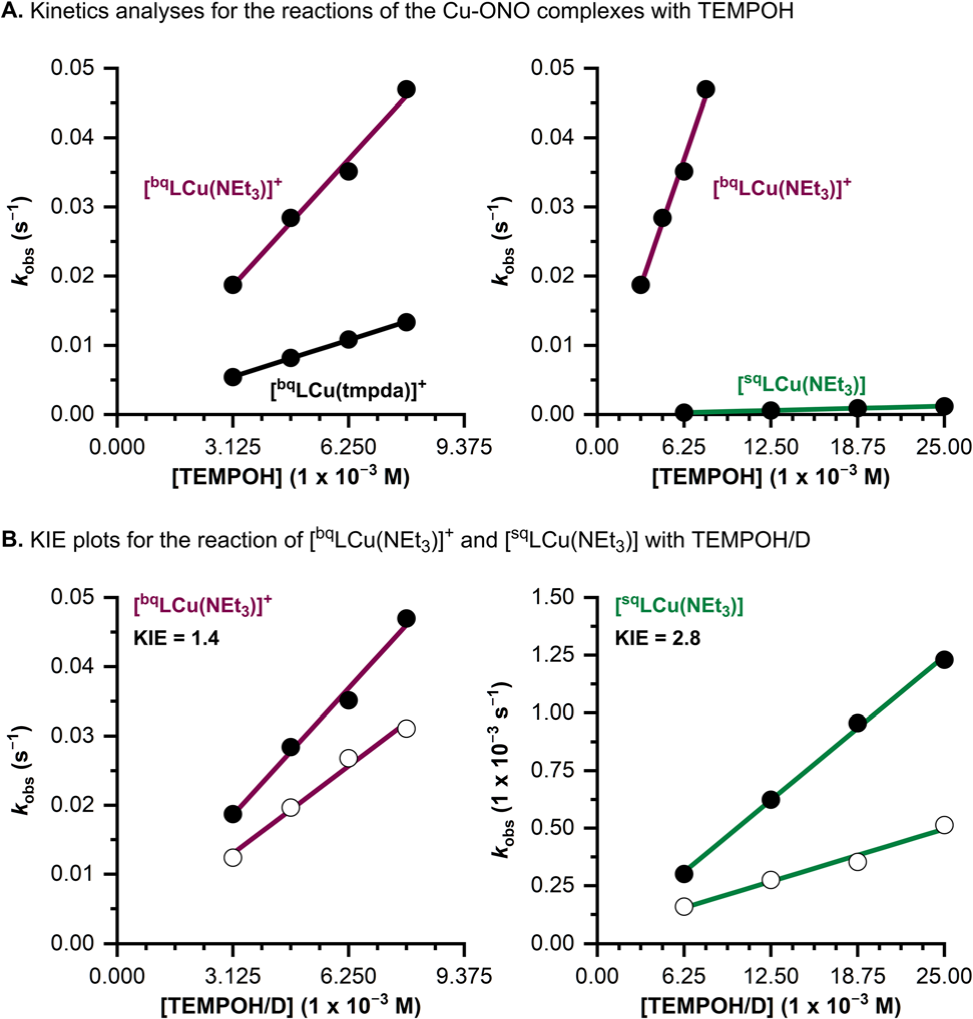
(A) Comparison of *k*_obs_ for the reactions of the Cu–ONO complexes with TEMPOH. (B) KIE analysis of [^bq^LCu(NEt_3_)]^+^ and [^sq^LCu(NEt_3_)] with TEMPOH (black circles) and TEMPOD (white circles).

The kinetics of the PCET reaction between the [^bq^LCu(A)]^+^ complexes (A = NEt_3_, tmpda) and TEMPOH was analyzed in DMF at room temperature under pseudo-first order conditions ([Cu] = 0.125 mM; [TEMPOH] = 3.125–7.8125 mM, see Fig. 6A and SI). Observed rate constants (*k*_obs_) were determined by fitting the initial decay of the UV-vis features of the [^bq^LCu(A)]^+^ complexes using the method of initial rates. A linear dependence of *k*_obs_ was observed, enabling determination of second order rate constants for the PCET reaction (*k*_2_; Fig. 6A and Table 2).

**Table 2:** Second-order rate constants (*k*_2_) and kinetic isotope effect (KIE) values for the reactions of the Cu–ONO complexes with TEMPOH and TEMPOD

Complex	TEMPOH	TEMPOD	KIE
	*k*_2_ (M^−1^ s^−1^)	*k*_2_ (M^−1^ s^−1^)	
[^sq^LCu(NEt_3_)]	0.050	0.018	2.8
[^bq^LCu(NEt_3_)]^+^	5.85	4.04	1.4
[^bq^LCu(tmpda)]^+^	1.70	—	—

Only minor differences in rate were observed upon varying the ancillary ligand, with the tmpda complex (*k*_2_ = 1.70 M^−1^ s^−1^) reacting slightly slower than the NEt_3_ complex (*k*_2_ = 5.85 M^−1^ s^−1^; Fig. 6A and Table 2). This trend is consistent with thermodynamic parameters (*E*_1/2_, p*K*_a_, and BDFE values), which similarly indicated minimal variation between the two complexes (see SI for a discussion of the thermochemistry [^bq^LCu(tmpda)]^+^). The modest rate decrease for [^bq^LCu(tmpda)]^+^ may instead reflect steric effects due to the bulkier bidentate tmpda ligand.

The PCET reaction between [^sq^LCu(NEt_3_)] and TEMPOH proceeded significantly more slowly (*k*_2_ = 0.050 M^−1^ s^−1^) than the reaction with [^bq^LCu(NEt_3_)]^+^ (see Fig. 6A and Table 2), consistent with the thermodynamic data indicating a lower driving force for H-atom transfer (BDFE = 62 kcal mol^−1^ for [^sq^LCu(NEt_3_)] *vs.* 69 kcal mol^−1^ for [^bq^LCu(NEt_3_)]^+^).

Kinetic isotope effect (KIE) experiments were performed using TEMPOD. KIEs values of 1.4 and 2.8 were determined for [^bq^LCu(NEt_3_)]^+^ and [^sq^LCu(NEt_3_)], respectively (Fig. 6B and Table 2). The observed KIEs for both complexes support a concerted proton–electron transfer (CPET) mechanism, in which both the proton and electron are transferred in a single kinetic step.

### Oxidation of weak C–H bonds

**Fig. 7:**
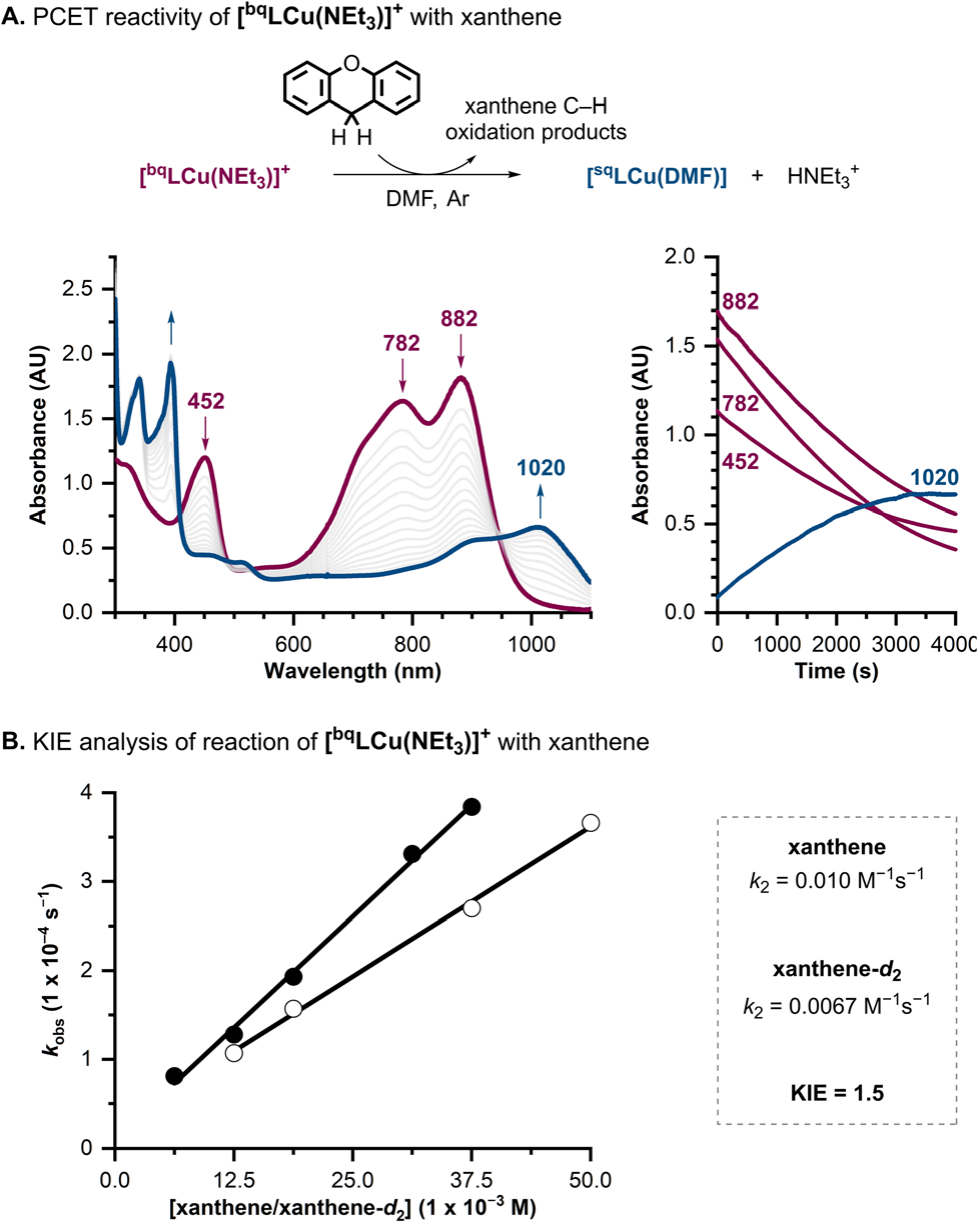
(A) Scheme for the reaction of [^bq^LCu(NEt_3_)]^+^ with xanthene, UV-vis spectra of the reaction, and time course of the absorption bands at 452, 782, 882, and 1020 nm ([Cu] = 0.125 mM, [xanthene] = 37.5 mM, 300 equiv.). (B) KIE analysis showing linear dependence of *k*_obs_ with [xanthene] (black circles) and [xanthene-d_2_] (white circles).

Once we established that [^bq^LCu(NEt_3_)]^+^ and [^sq^LCu(NEt_3_)] complexes were able to accept H-atom equivalents from substrates containing weak O–H bonds, we decided to evaluate their reactivity towards substrates containing weak C–H bonds, including xanthene (BDFE 70.2 kcal mol^−1^ in DMSO) and 9,10-dihydroanthracene (DHA; BDFE = 72.9 kcal mol^−1^ in DMSO).^15^ While [^sq^LCu(NEt_3_)] did not react with any of the C–H substrates tested, the [^bq^LCu(NEt_3_)]^+^ complex reacted with xanthene (Fig. 7A). The reaction was followed by UV-vis spectroscopy, and the spectral changes observed were similar to those recorded in the reaction between [^bq^LCu(NEt_3_)]^+^ and TEMPOH, suggesting that [^bq^LCu(NEt_3_)]^+^ underwent a 1H^+^/1e^−^ reductive protonation. Like the TEMPOH reaction, the Cu product [^sq^LCu(DMF)] was partially recovered, albeit in lower yield, likely due to self-decay of [^bq^LCu(NEt_3_)]^+^ over the much longer time-course of the reaction.

The reaction between [^bq^LCu(NEt_3_)]^+^ and xanthene (10 equiv.) under inert atmosphere was analyzed by NMR spectroscopy. The products were identified and quantified as bixanthene (0.3% yield), xanthydrol (9% yield), and xanthone (7% yield). Reported yields are referenced to [Cu] and normalized based on the electron stoichiometry for the oxidation of xanthene to each product (see SI for details). The formation of xanthydrol (and xanthone) is attributed to the reaction of oxidized xanthene with adventitious water present in the NMR sample.

Kinetic studies of the reaction between [^bq^LCu(NEt_3_)]^+^ and xanthene were carried out under pseudo-first order conditions in DMF at room temperature under argon ([Cu] = 0.125 mM, [xanthene] = 6.27–37.5 mM, see SI). Values of *k*_obs_ were obtained by fitting the initial decay of the peak at 782 nm using the method of initial rates. A linear dependence of *k*_obs_ on [xanthene] was observed, allowing determination of the second-order rate constant for the reaction (*k*_2_ = 0.010 M^−1^ s^−1^; Fig. 7B). This rate is considerably slower than TEMPOH (*k*_2_ = 4.1 M^−1^ s^−1^), consistent with the higher BDFE of the C–H bond in xanthene. Kinetic analysis using deuterated xanthene (xanthene-d_2_) yielded a second-order rate constant of 0.0067 M^−1^ s^−1^, corresponding to a primary KIE of 1.5 and consistent with C–H bond cleavage in the rate-determining step of the reaction.

### Contextualization of our findings

In 1999, Wieghardt and coworkers reported that the complex [^sq^LCu(NEt_3_)] was able to catalyze the aerobic dehydrogenation of alcohols, mimicking the reactivity of galactose oxidase (*i.e.*, RCH_2_OH + O_2_ → RCHO + H_2_O_2_).^19^ The authors suggested that [^sq^LCu(NEt_3_)] coordinated the substrate in the alkoxide form (*via* protonation of the ^sq^L˙^2−^ ligand), triggering an intramolecular PCET event to produce a cuprous protonated complex ([(^cat^LH_2_)Cu^I^(NEt_3_)] or [(^cat^LH)Cu]^−^ + HNEt_3_^+^) and the dehydrogenation product. As we have shown in this article, [^sq^LCu(NEt_3_)] is a poor H-atom acceptor (reacts partially with TEMPOH) but it can efficiently promote the 2H^+^/2e^−^ dehydrogenation of ethanol by virtue of substrate coordination/deprotonation followed by intramolecular PCET (formally a stepwise proton transfer, H-atom transfer, electron transfer for an overall 2H^+^/2e^−^ process). However, the oxidation reactions described herein are mechanistically distinct because they do not involve substrate pre-coordination and involve a single-step concerted proton-coupled electron transfer (CPET). It should be noted that the 2H^+^/2e^−^ dehydrogenation of 1 equiv. of CH_3_CH_2_OH is thermodynamically more favorable than the dehydrogenation of 2 equiv. of TEMPOH (*i.e.*, the BDFE_avg_ of the CH_3_CH_2_OH/CH_3_CHO couple is 56.1 kcal mol^−1^ in CH_3_CN,^20^ while the BDFE_O–H_ of the TEMPOH is 66 kcal mol^−1^ in CH_3_CN).^15^

To the best of our knowledge, [^bq^LCu(NEt_3_)]^+^ is the first mononuclear Cu complex bound by a redox-active ligand capable of performing multi-electron, multi-proton PCET with both C–H and O–H bond substrates. Previous complexes developed in our laboratory, including Cu^II^OH and Ni^II^OH species supported by tridentate tris(amido) (NNN) redox-active ligands, were competent for multiple H-atoms abstractions from weak O–H bonds but were unreactive toward C–H substrates. This difference is notable because the thermodynamic driving forces (BDFE) for H-atom abstraction by the [(^bq^NNN)CuOH] and [(^bq^NNN)NiOH] complexes (76 and 72 kcal mol^−1^, respectively) are slightly higher than that of [^bq^LCu(NEt_3_)]^+^ (69 kcal mol^−1^).^21^

**Fig. 8:**
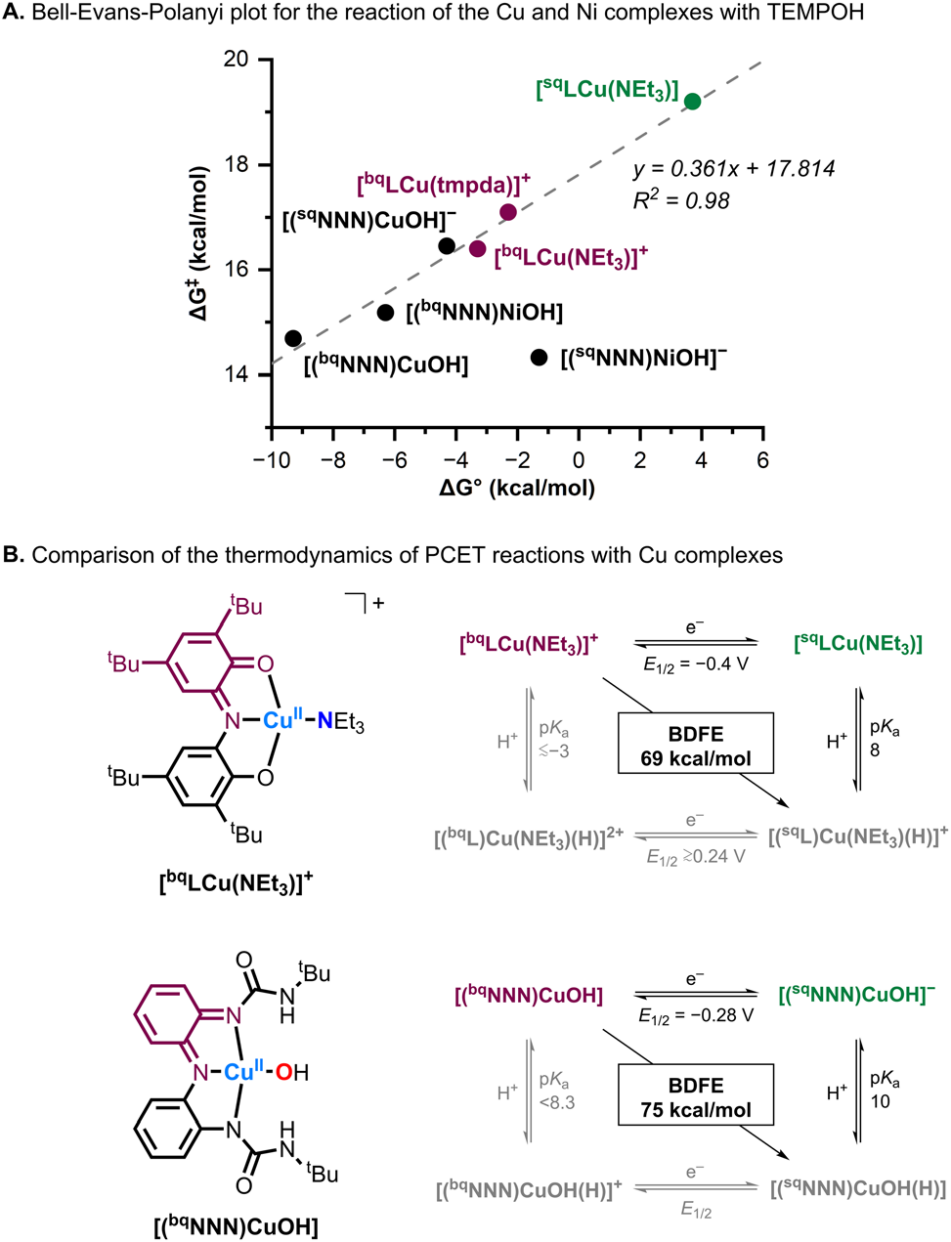
(A) Bell–Evans–Polanyi plot for the reaction of “high-“ and “intermediate-valent” redox-active ligand complexes with TEMPOH. (B) Comparison of the thermodynamics for the reductive protonation of “high-valent” [^bq^LCu(NEt_3_)]^+^ and [(^bq^NNN)CuOH].

These trends are illustrated in the Bell–Evans–Polanyi (BEP) plot (Fig. 8A) which compares the thermodynamics of the PCET reaction of Cu–NNN, Ni–NNN, and Cu–ONO complexes with TEMPOH. The BEP plot relates the thermodynamic driving force (Δ*G*^0^; calculated from the BDFE difference between the metal complex and TEMPOH) to the free energy of activation (Δ*G*^‡^; derived from the Eyring equation). All but one of complexes examined, including [^bq^LCu(NEt_3_)]^+^ and [^sq^LCu(NEt_3_)], fall on a line with a slope ≈ 0.32, consistent with synchronous coupled proton-electron transfer (CPET).^22^ The sole outlier, [(^sq^NNN)NiOH]^−^, exhibits enhanced PCET rates attributed to extensive H-atom tunneling.^21^

Particularly striking is the ability of [^bq^LCu(NEt_3_)]^+^ to oxidize C–H bonds, in contrast to [(^bq^NNN)CuOH], despite the latter being both a better 1H^+^/1e^−^ acceptor and a stronger oxidant with higher basicity (Fig. 8B).^13,21^ This suggests that non-thermodynamic factors, such as sterics, facilitate C–H bond activation in the ONO system. Stack and coworkers have shown that the PCET reactivity of a series of [(L)_2_Cu^III^_2_(O^2−^)_2_]^2+^ systems with similar 1H^+^/1e^−^ potentials (*i.e.*, the BDFE associated with their 1H^+^/1e^−^ reductive protonation was computed to be within 3 kcal mol^−1^) was highly dependent on steric factors, in which “accessible” Cu_2_O_2_ cores reacted with C–H bonds while the “less accessible” ones did not.^23^ Analogously, our findings underscore that maximizing thermodynamic driving force (increasing BDFE) is not, on its own, sufficient to achieve metalloenzyme-like C–H and O–H bond activation.

Our prior work on the reactivity of [(^bq^NNN)CuOH] and related complexes suggested that the PCET events carried out by these cupric complexes were ligand-centered, in which the proton(s) and the electron(s) were transferred to the NNN scaffold.^13,24^ Conversely, in the PCET reactions with [^bq^LCu(NEt_3_)]^+^ we observed the formation of HNEt_3_^+^ and reduction of the redox-active scaffold, which suggests a “separated” coupled-proton electron transfer similar to the C–H oxidation in cytochrome P450 in which the proton and the electron are transferred to different “sites”.^25^

## Conclusions

In this work, we describe the PCET reactivity of Cu complexes supported by redox-active tridentate ONO ligand with weak C–H and O–H bond substrates in detail. Complex [^bq^LCu(NEt_3_)]^+^ was found act as a net 3H^+^/3e^−^ oxidant capable of abstracting H-atoms from C–H and O–H with BDFEs lower than 72 kcal mol^−1^. Complex [^sq^LCu(NEt_3_)] functioned as a net 2H^+^/2e^−^ oxidant with weak O–H bonds with BDFEs lower than 66 kcal mol^−1^. Thermodynamic and kinetic analyses reveal that the PCET reactivity of these systems is consistent with synchronous CPET. Particularly notable is the ability of [^bq^LCu(NEt_3_)]^+^ to oxidize weak C–H bonds despite its lower BDFE compared to previous “high-valent” Cu- and Ni-based systems developed by our laboratory, which are stronger oxidants with higher basicity. This suggests that non-thermodynamic factors such as sterics, coordination geometry, and other factors may play key roles in facilitating C–H bond activation in the ONO system. Our findings underscore that maximizing thermodynamic driving force (*i.e.*, increasing BDFE) is not, on its own, sufficient to achieve metalloenzyme-like C–H and O–H bond activation. Future work will focus on elucidating the mechanistic basis of C–H activation in these systems to guide the design of new complexes capable of activating stronger C–H bonds.

## Author contributions

DDH: conceptualization, formal analysis, investigation (synthesis, UV-vis, NMR, EPR), methodology, validation, visualization, writing – original draft, writing – review & editing; DY: investigation (UV-vis), writing – review & editing; IGB: conceptualization, formal analysis, funding acquisition, resources, supervision, validation, writing – original draft, writing – review & editing.

## Conflicts of interest

There are no conflicts to declare.

## Supplementary Material

SC-016-D5SC07166F-s001

## Data Availability

The data supporting this article have been included as part of the supplementary information (SI). Supplementary information: Experimental details, UV-vis, NMR, EPR spectra. See DOI: https://doi.org/10.1039/d5sc07166f.
