# Supplementary material for: Oxidation of C–H and O–H bonds by a copper complex inspired by the Cu(ii)–tyrosyl species formed in LPMOs
Source: Chem Sci. 2025 Oct 17;16(47):22473–80. doi: 10.1039/d5sc07166f (PMC12551145; doi:10.1039/d5sc07166f)
Supplement: SC-016-D5SC07166F-s001 [file SC-016-D5SC07166F-s001.pdf]

Supplementary Information (SI) for Chemical Science.
This journal is © The Royal Society of Chemistry 2025

Supplementary Information

for

Oxidation of C–H and O–H Bonds by a Copper Complex Inspired by the Cu(II)–Tyrosyl Species Formed in LPMOs

David D. Hebert[†], Daniel Ye[†], Isaac Garcia-Bosch[*,†]

[†]*Department of Chemistry, Carnegie Mellon University, Pittsburgh, Pennsylvania 15213, United States.*

[*]*Corresponding Author*

igarciab@andrew.cmu.edu (Isaac Garcia-Bosch)

Contents

1. Experimental 3
2. Reactivity Studies with PCET Reagents 7
3. Stoichiometry 19
4. ^1H NMR of the ONO Ligand in the Presence of Cu^I and Triethylamine 36
5. Estimation of BDFE and pK_a Values 42
6. Kinetics 51
7. Reaction of $[^{bq}LCu(NEt_3)]^+$ with xanthene monitored by ^1H NMR 58
8. References 65
9. Appendix 67

1. Experimental

General Considerations. All air-free manipulations, including the preparation of stock solutions for UV-vis, NMR, and EPR experiments were carried out in an MBRAUN UNIlab Pro SP glovebox system under a nitrogen atmosphere.

Materials. All reagents were obtained from commercial suppliers and used without further purification unless otherwise noted. [^{sq}LCu(NEt_3)] and [^{sq}LCu(tmpda)] were prepared as previously reported.[1] The following compounds were prepared according to literature procedures: 1,4-dihydroxynaphthalene (1,4-H_2NQ), 2,6-dimethyl-1,4-hydroquinone (2,6-Me_2-H_2Q), and 2,6-dichloro-1,4-hydroquinone (2,6-Cl_2-H_2Q);[2] 2,2,6,6-tetramethyl-1-hydroxypiperidine (TEMPOH);[3] ferrocenium hexafluorophosphate ($FcPF_6$);[4] xanthene-d_2;[5] N,N-dimethylformamidinium triflate (DMF•TfOH);[6] bis(3,5-di-*tert*-butyl-2-hydroxyphenyl)amine ($^{cat}LH_3$);[7] bis(3,5-di-*tert*-butyl-2-hydroxyphenyl)ammonium trifluoroacetate ([$^{cat}LH_4$](CF_3COO)).[8] Deuterated TEMPOH (TEMPOD) was prepared in the same manner as TEMPOH, with D_2O and acetone-d_6 used in place of H_2O and acetone. Deuteration (>95%) of TEMPOD and xanthene-d_2 was confirmed by ^{1}H NMR. Dimethylformamide (DMF) was dried over 4 Å molecular sieves, distilled under reduced pressure, sparged with N_2, and stored in the glovebox. All other solvents were purchased at the highest level of purity and further dried, deoxygenated, and purified by passage through a MBRAUN MB SPS-7 activated alumina solvent purification system. Deuterated solvents were purchased from Cambridge Isotope Laboratories (Tewksbury, MA, USA) and used as received.

Spectroscopic Methods. UV-vis spectra were collected using an Agilent Cary 8454 diode array spectrophotometer equipped with a Unisoku CoolSpeK cryostat (UNISOKU Co., Hirakata, Japan) for temperature control and magnetic stirring. Data acquisition was performed using the Agilent UV-Visible ChemStation software. Spectral fitting and data processing were carried out using the *uv_pro* Python library.[9] All UV-vis spectra were collected at 25 °C under a flow of argon gas using a custom-made 1 cm pathlength quartz Schlenk cuvette. X-band EPR spectra of frozen solutions were recorded on a Bruker ELEXSYS spectrometer equipped with an Oxford liquid helium cryostat and a Bruker bimodal cavity. The microwave frequency was calibrated with a frequency counter, and the magnetic field was measured with an NMR gaussmeter. Sample temperatures were calibrated using a CX-1050 Cernox sensor mounted inside an EPR tube. A modulation amplitude of 0.5 mT and frequency of 100 kHz were used for all EPR spectra. ^{1}H NMR spectra were collected using a Bruker Avance NEO 500 MHz NMR spectrometer, with 16–64 scans collected per spectrum. All NMR spectra were calibrated to the DMF solvent residual aldehyde signal (δ = 8.03 ppm).

UV-vis Reactivity Studies with PCET Reagents. DMF solutions of **[^{sq}LCu(NEt$_3$)]** (7.5 m) and the desired PCET substrate (375 mM) were prepared and loaded into gastight microsyringes. A Schlenk cuvette was charged with DMF (2.9 mL), a magnetic stir bar, and sealed with a rubber septum. The cuvette and syringes were removed from the glovebox, and the cuvette was used to blank the UV-vis spectrometer. **[^{sq}LCu(NEt$_3$)]** solution (50 μL) was injected into the cuvette through the septum. The substrate solution (50 μL) was then added via microsyringe. All reactions were performed at 25 °C.

The same procedure was used for reactions with **[^{bq}LCu(NEt$_3$)]$^+$**. The "high-valent" complex was generated *in situ* by addition of 50 μL of a DMF solution of $FcPF_6$ (7.5 mM, 1 equiv; prepared in the glovebox) prior to addition of substrate.

Protonation of [^{sq}LCu(NEt$_3$)], [^{cat}LCu(NEt$_3$)]$^-$, and [^{bq}LCu(NEt$_3$)]$^+$ with DMF•TfOH Monitored by UV-vis Spectroscopy. Stock solutions of **[^{sq}LCu(NEt$_3$)]** (7.5 mM), and DMF•TfOH (7.5 mM) were prepared in DMF and transferred into 250 μL gastight microsyringes. A Schlenk cuvette was charged with DMF (2.8 mL), a magnetic stir bar, and sealed with a rubber septum. The cuvette and syringes were removed from the glovebox, and the cuvette was used to blank the UV-vis spectrometer. **[^{sq}LCu(NEt$_3$)]** stock solution (50 μL) was injected into the cuvette through the septum. The complex was titrated with incremental additions of DMF•TfOH solution (12.5 μL per addition, corresponding to 0.25 equiv per addition) at 25 °C until a total of 4 equiv of acid had been added. After each addition, the solution was allowed to equilibrate until no further spectral changes were observed before the next addition was made.

The same procedure was used in titrations of **[^{cat}LCu(NEt$_3$)]$^-$** and **[^{bq}LCu(NEt$_3$)]$^+$**. The reduced and oxidized complexes were generated *in situ* by addition of 50 μL of DMF solutions of cobaltocene ($CoCp_2$) or $FcPF_6$ (7.5 mM, 1 equiv; prepared in a glovebox), respectively, to the cuvette prior to addition of acid.

Additional details on the spectral fitting procedure used to determine the relative concentrations of Cu species formed during the titrations are provided in **Section 2** below.

Protonation of [^{sq}LCu(NEt$_3$)] with DMF•TfOH Monitored by ^{1}H NMR Spectroscopy. Stock solutions were prepared in DMF-d_7 solutions as follows: **[^{sq}LCu(NEt$_3$)]** (7.5 mM), DMF•TfOH (30.0 mM), and 1,3,5-trimethoxybenzene (37.5 mM). The solutions were transferred into gastight microsyringes. Four 1 mL scintillation vials were each charged with DMF-d_7 (300, 275, 250, and 200 μL), **[^{sq}LCu(NEt$_3$)]** stock solution (200 μL), and 1,3,5-trimethoxybenzene solution (50 μL). To each vial, the appropriate volume of DMF•TfOH solution (50, 75, 100, or 150 μL) was added to give 1.0, 1.5, 2.0, and 3.0 equivalents relative to [Cu], respectively. Each solution had a final volume of 600 μL with [Cu] = 2.5 mM. The vials were capped and shaken to ensure mixing. Each solution was then transferred to NMR tubes and removed from the glovebox for NMR analysis. Quantification was performed using the resonances of 1,3,5-trimethoxybenzene as an internal standard.

PCET Reaction with 1,4-H_2NQ Monitored by ^{1}H NMR Spectroscopy. Stock solutions were prepared in DMF-*d_7* as follows: **[^{sq}LCu(NEt_3)]** (7.5 mM), $FcPF_6$ (30.0 mM), 1,4-H_2NQ (150 mM), and 1,3,5-trimethoxybenzene (37.5 mM). The solutions were transferred into gastight microsyringes.

For the reaction with **[^{sq}LCu(NEt_3)]**, a 1 mL scintillation vial was charged with DMF-*d_7* 150 μL, **[^{sq}LCu(NEt_3)]** stock solution (200 μL), and 1,3,5-trimethoxybenzene solution (200 μL). For the reaction with **[^{bq}LCu(NEt_3)]$^+$**, a second vial was charged with DMF-*d_7* (100 μL), **[^{sq}LCu(NEt_3)]** stock solution (200 μL), $FcPF_6$ solution (50 μL) and 1,3,5-trimethoxybenzene solution (200 μL). To each vial, 1,4-H_2NQ solution (50 μL) was then added, resulting in a final volume of 600 μL and [Cu] = 2.5 mM for both samples. The vials were capped, gently shaken to mix, and allowed to incubate at ambient temperature for 1 h.

After incubation, the **[^{sq}LCu(NEt_3)]** reaction mixture changed color from green to purple, while the **[^{bq}LCu(NEt_3)]$^+$** mixture remained green. The solutions were then transferred to NMR tubes and removed from the glovebox for NMR analysis. Quantification was performed using the resonances of 1,3,5-trimethoxybenzene as an internal standard.

Quantification of TEMPO Radical by EPR Spectroscopy. Stock solutions were prepared in DMF as follows: **[^{sq}LCu(NEt_3)]** (6.0 mM), $FcPF_6$ (12 mM), TEMPOH (60 mM). A stock solution of **[^{bq}LCu(NEt_3)]$^+$** (3 mM) was prepared by diluting the **[^{sq}LCu(NEt_3)]** stock solution (500 μL) with DMF (250 μL), followed by addition of $FcPF_6$ solution (250 µL). The solutions were transferred into gastight microsyringes for subsequent use. For the reaction with **[^{sq}LCu(NEt_3)]**, a 1 mL scintillation vial was charged with DMF (150 μL), **[^{sq}LCu(NEt_3)]** stock solution (50 μL), and TEMPOH solution (100 μL). For the reaction with **[^{bq}LCu(NEt_3)]$^+$**, a second vial was charged with DMF (100 μL), **[^{bq}LCu(NEt_3)]$^+$** stock solution (100 μL) and TEMPOH solution (100 μL). The vials were capped and gently shaken to ensure mixing. After 2 min, the solutions (each with a volume of 300 μL and [Cu] = 1 mM) were transferred to quartz EPR tubes, removed from the glovebox, and frozen in liquid N_2 for analysis.

To confirm the concentration of **[^{bq}LCu(NEt_3)]$^+$** for accurate determination of the yield of TEMPO radical, an additional reference sample was prepared by mixing **[^{bq}LCu(NEt_3)]$^+$** stock solution (100 μL) and DMF (200 μL).

^{1}H NMR of the ONO Ligand in the Presence of Cu^I and Triethylamine. In a glovebox, ONO ligand ($^{cat}LH_3$; 5.1 mg, 12 μmol) and [$(CH_3CN)_4$Cu]PF_6 (4.5 mg, 12 μmol, 1.0 equiv) weighed into separate 1 mL scintillation vials. A stock solution of triethylamine (480 mM) was prepared in DMF-*d_7*. The ligand was dissolved in DMF-*d_7* (600 μL), and triethylamine stock solution (25 μL, 1.0 equiv) was added via microsyringe. [$(CH_3CN)_4$Cu]PF_6 was then added as a solid, resulting in a color change from colorless to yellow. The solution was transferred to an NMR tube, capped, sealed with PTFE tape, and removed from the glovebox for analysis.

The same procedure was used for the protonated ONO ligand ([$^{cat}LH_4$](CF_3COO); 6.5 mg, 12 μmol) in the presence of [$(CH_3CN)_4$Cu]PF_6 and triethylamine. In this case, addition of [$(CH_3CN)_4$Cu]PF_6 to the ligand/triethylamine solution resulted in a color change from colorless to blue, along with the

formation of finely dispersed metallic Cu from disproportionation of Cu^I. Both procedures above were also repeated in the absence of triethylamine (see **Section 4** below).

Estimation of BDFE and pK_a Values. The equilibrium positions of the reaction between **[^{sq}LCu(NEt$_3$)]** and TEMPOH, and **[^{bq}LCu(NEt$_3$)]$^+$** were performed using the same procedure described in the "UV-vis Reactivity Studies with PCET Reagents" section above. The equilibrium position was determined by monitoring the reactions until no further spectral changes were observed. Additional details on the calculation of BDFE and pK_a values for the complexes are provided in **Section 5** below.

Protonation of [^{cat}LCu(NEt$_3$)]$^-$ with Phenols. Stock solutions of **[^{sq}LCu(NEt$_3$)]** (7.5 mM), $CoCp_2$ (7.5 mM), and the desired phenolic acid (15 mM) were prepared in DMF and transferred into 250 μL gastight microsyringes. A Schlenk cuvette was charged with DMF (2.8 mL), a magnetic stir bar, and sealed with a rubber septum. The cuvette and syringes were removed from the glovebox, and the cuvette was used to blank the UV-vis spectrometer. **[^{sq}LCu(NEt$_3$)]** stock solution (50 *μ*L) was injected into the cuvette through the septum, and $CoCp_2$ solution (50 *μ*L) was added to generate **[^{cat}LCu(NEt$_3$)]$^-$**. The complex was titrated with incremental additions of phenolic acid solution (25 *μ*L per addition, corresponding to 1.0 equiv per addition) at 25 °C until a total of 10 equiv of acid had been added. After each addition, the solution was allowed to equilibrate until no further spectral changes were observed before the next addition was made.

Kinetics Studies. Stock solutions of Cu complex (7.5 mM) and the desired substrate (375–750 mM) were prepared in DMF solutions and transferred into gastight microsyringes. A Schlenk cuvette was charged with DMF (2.8 mL), a magnetic stir bar, and sealed with a rubber septum. The cuvette and syringes were removed from the glovebox. The cuvette was used to blank the UV-vis spectrometer, and Cu complex stock solution (50 *μ*L) was injected into the cuvette through the septum. The desired amount of substrate solution was then injected into the cell.

The same procedure was used for reactions with **[^{bq}LCu(NEt$_3$)]$^+$**. The "high-valent" complex was generated *in situ* by addition of 50 *μ*L of a DMF solution of $FcPF_6$ (7.5 mM, 1 equiv; prepared in the glovebox) prior to addition of substrate. Additional details on the kinetic analysis and fitting procedure are provided in **Section 6** below.

PCET Reaction with Xanthene Monitored by ^{1}H NMR Spectroscopy. In a glovebox, **[^{sq}LCu(NEt$_3$)]** (3.7 mg, 6.3 *μ*mol) was dissolved in DMF-d_7 (0.8 mL) in a 1 mL scintillation vial. Solid $FcPF_6$ (2.2 mg, 6.6 *μ*mol, 1.0 equiv) was added to the solution in portions with intermittent mixing to generate the "high-valent" **[^{bq}LCu(NEt$_3$)]$^+$** complex. Xanthene (12 mg, 66 *μ*mol, 10 equiv) was then added as a solid, and the vial was capped and gently shaken to ensure mixing. The final solution (0.8 mL, [Cu] = 8.0 mM) was transferred to an NMR tube, capped, sealed with PTFE tape, and removed from the glovebox for analysis. NMR spectra were recorded over the course of one week to monitor the formation of xanthene oxidation products. After each measurement, the sample was returned to the glovebox to minimize exposure to atmospheric oxygen. After one week, solid 1,3,5-trimethoxybenzene (1.5 mg, 8.9 *μ*mol) was added to the sample to serve as an internal standard for quantification of the xanthene oxidation products.

2. Reactivity Studies with PCET Reagents

The reactivity of the **[LCu(NEt$_3$)]^n** complexes towards PCET reagents was screened by UV-vis spectroscopy. Spectra were collected at regular intervals over the course of the reaction, and the progress of the reaction was monitored by following changes in the characteristic absorption bands of the Cu complex. Reactivity was assessed qualitatively based on the extent of spectral conversion (e.g., **[^{bq}LCu(NEt$_3$)]$^+$** → **[^{sq}LCu(solv)]** or **[^{sq}LCu(NEt$_3$)]** → **[(^{cat}LH$_2$)Cu^I(NEt$_3$)]**). This qualitative screening provided preliminary insight into the bond dissociation free energy (BDFE) of the complexes by identifying the substrates of known BDFE with which they could undergo PCET.

Scheme S1. The PCET reagents used in this study and their literature BDFE values.

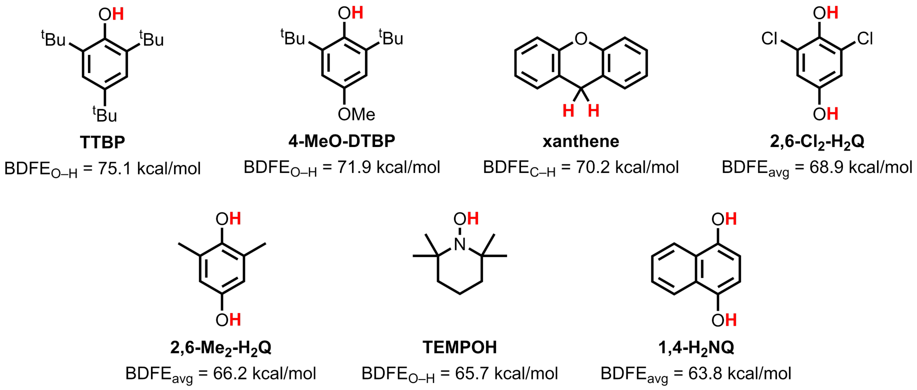

The BDFE values of phenols, TEMPOH, and the hydroquinone substrates are known in DMF.[10] For the 2H$^+$/2e$^-$ donor hydroquinone substrates, the average BDFE of both O–H bonds (BDFE$_{avg}$) is reported. The BDFE of xanthene has been reported in DMSO,[11] which we estimate to be similar to the BDFE in DMF.[12]

2.1. [^{bq}LCu(NEt$_3$)]$^+$

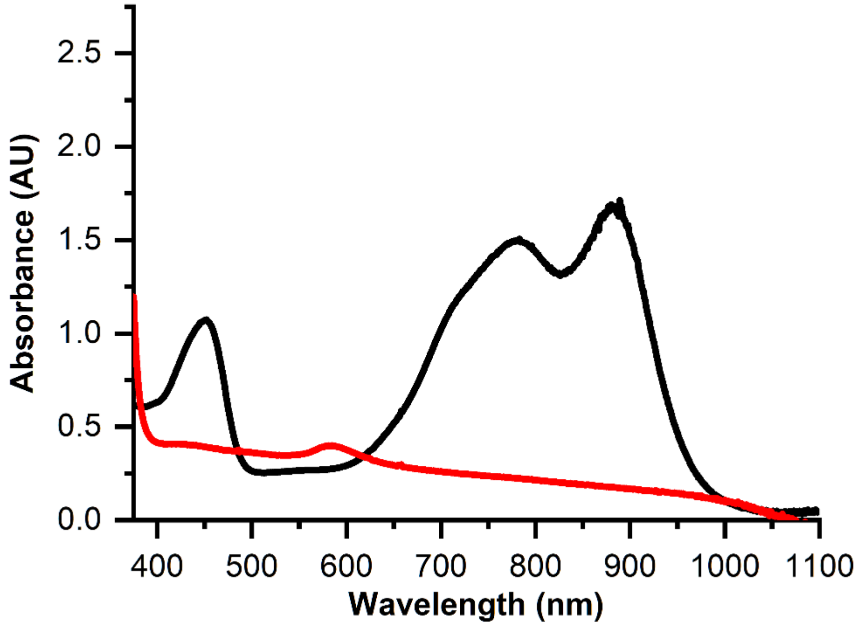

Figure S1. Initial (black trace) and final (red trace) UV-vis spectra for the reaction of **[^{bq}LCu(NEt$_3$)]$^+$** (0.125 mM) with 1,4-H_2NQ (50 equiv; BDFE$_{avg}$ = 63.8 kcal/mol)[10] in DMF at 25 °C. The reaction proceeds completely, including complete consumption of the intermediate **[^{sq}LCu(solv)]** species (see **Figure 4** in the main text).

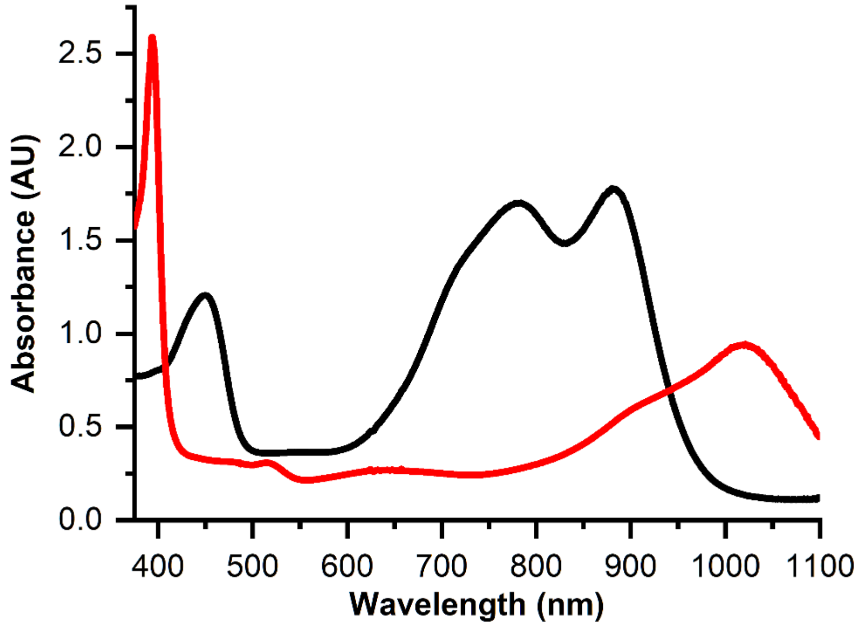

Figure S2. Initial (black trace) and final (red trace) UV-vis spectra for the reaction of **$[^{bq}LCu(NEt_3)]^+$** (0.125 mM) with TEMPOH (50 equiv; $BDFE_{O\text{–}H}$ = 65.7 kcal/mol)[10] in DMF at 25 °C. The reaction proceeds completely.

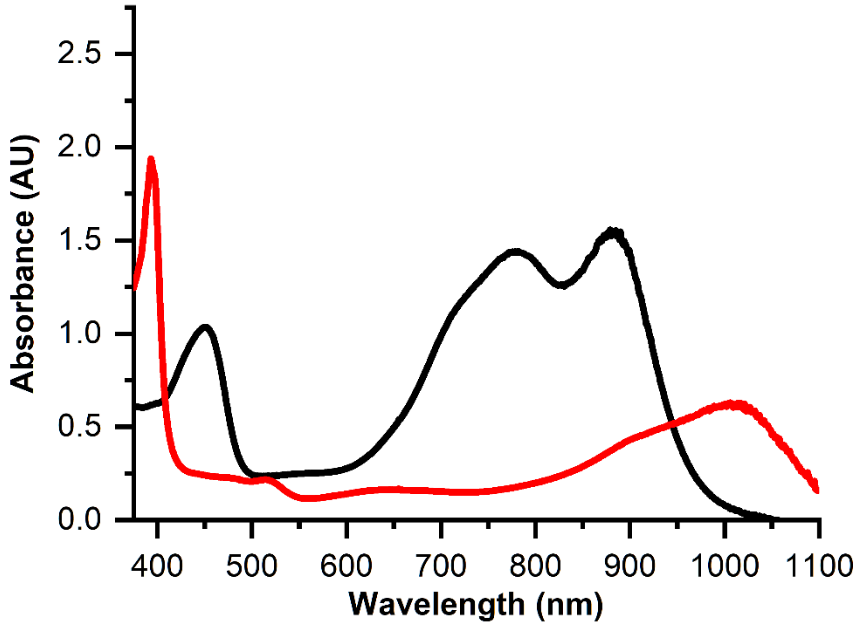

Figure S3. Initial (black trace) and final (red trace) UV-vis spectra for the reaction of **[^{bq}LCu(NEt$_3$)]$^+$** (0.125 mM) with 2,6-Me$_2$-H$_2$Q (50 equiv; BDFE$_{avg}$ = 66.2 kcal/mol)[10] in DMF at 25 °C. The reaction proceeds completely.

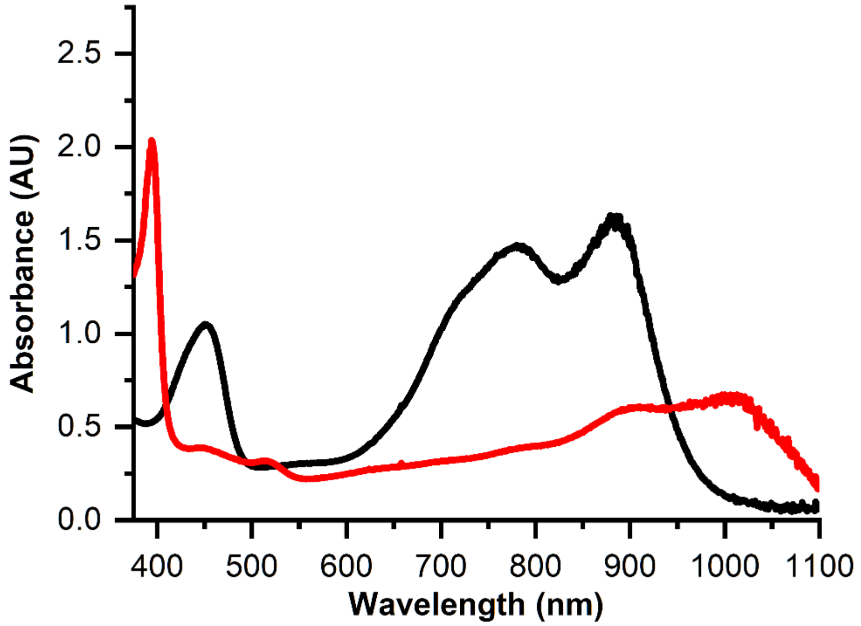

Figure S4. Initial (black trace) and final (red trace) UV-vis spectra for the reaction of **$[^{bq}LCu(NEt_3)]^+$** (0.125 mM) with 2,6-Cl_2-H_2Q (50 equiv; $BDFE_{avg}$ = 68.9 kcal/mol)[10] in DMF at 25 °C. The reaction proceeds completely.

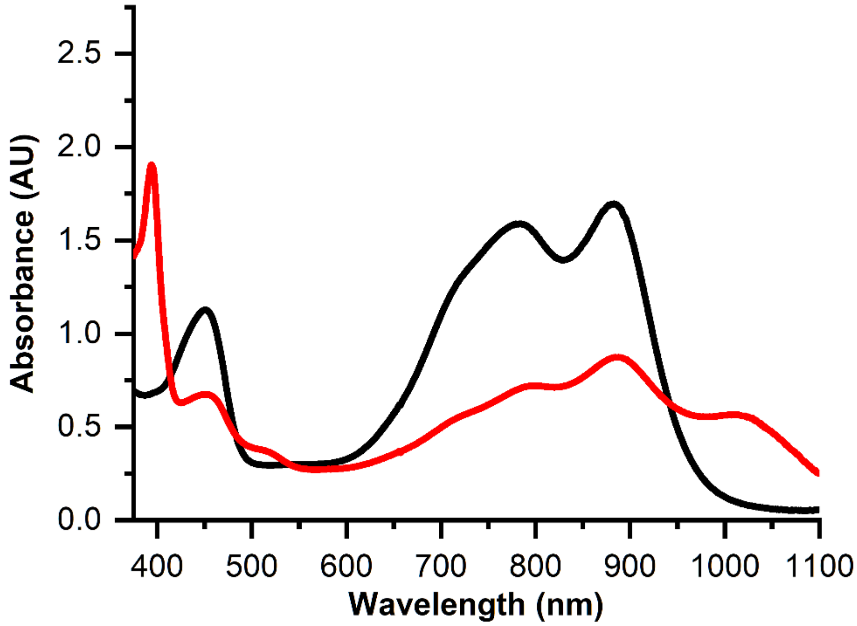

Figure S5. Initial (black trace) and final (red trace) UV-vis spectra for the reaction of **[^{bq}LCu(NEt$_3$)]$^+$** (0.125 mM) with 4-MeO-DTBP (37.5 equiv; BDFE$_{O-H}$ = 71.9 kcal/mol)[10] in DMF at 25 °C. The complex is only partially consumed at the end of the reaction (equilibrium).

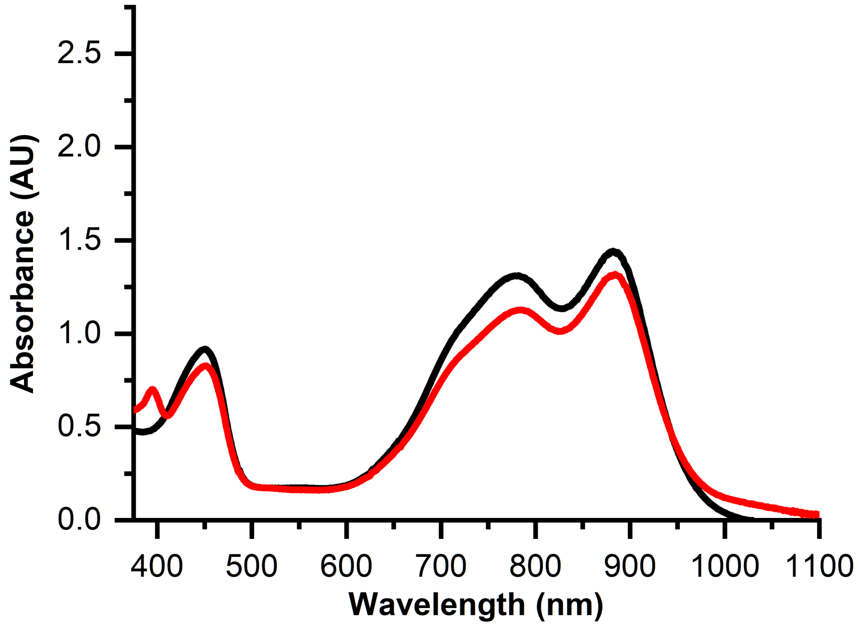

Figure S6. Initial (black trace) and final (red trace) UV-vis spectra for the reaction of **[^{bq}LCu(NEt$_3$)]$^+$** (0.125 mM) with TTBP (25 equiv; BDFE$_{O-H}$ = 75.1 kcal/mol)[10] in DMF at 25 °C. The observed decrease in absorbance is due to self-decay of the complex, and no TTBP• radical features[13] are detected, indicating no reaction.

2.2. [^{sq}LCu(NEt$_3$)]

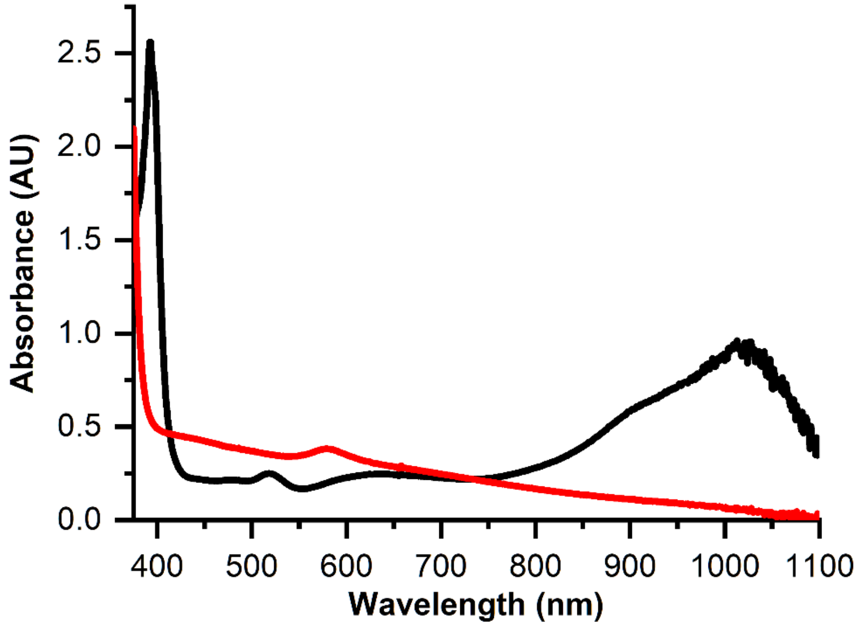

Figure S7. Initial (black trace) and final (red trace) UV-vis spectra for the reaction of **[^{sq}LCu(NEt$_3$)]** (0.125 mM) with 1,4-H_2NQ (50 equiv; BDFE$_{avg}$ = 63.8 kcal/mol)[10] in DMF at 25 °C. The reaction proceeds completely.

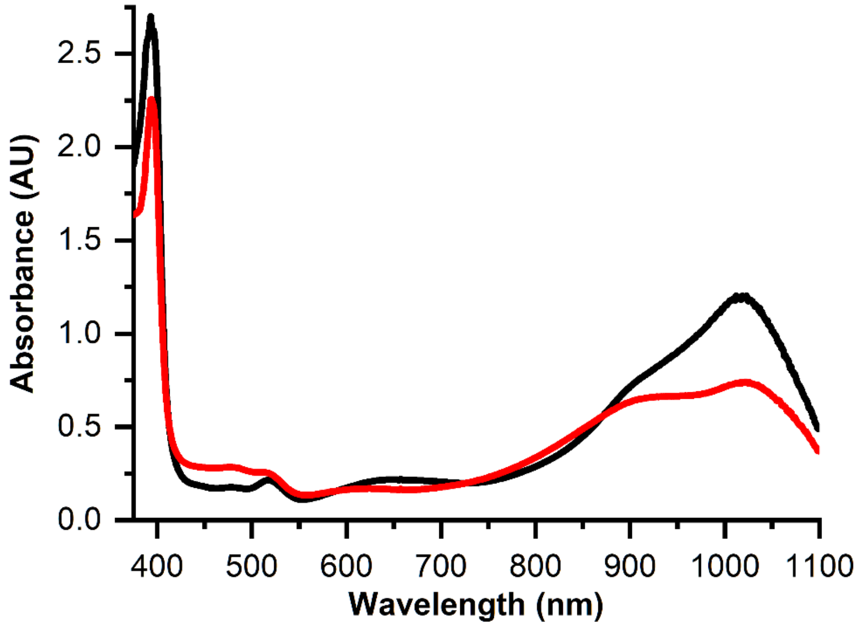

Figure S8. Initial (black trace) and final (red trace) UV-vis spectra for the reaction of **[^{sq}LCu(NEt$_3$)]** (0.125 mM) with TEMPOH (50 equiv; BDFE$_{O-H}$ = 65.7 kcal/mol)[10] in DMF at 25 °C. The complex is only partially consumed at the end of the reaction (equilibrium).

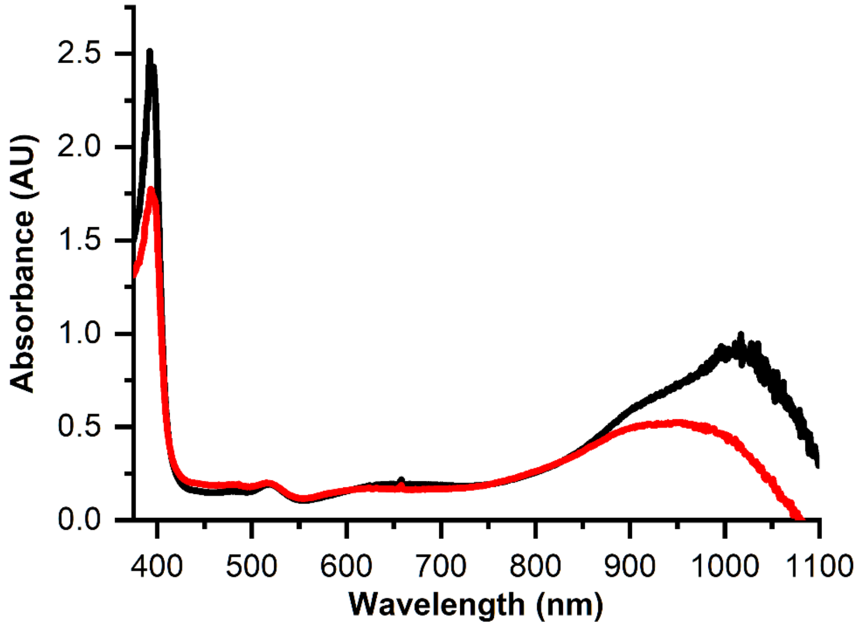

Figure S9. Initial (black trace) and final (red trace) UV-vis spectra for the reaction of **[^{sq}LCu(NEt$_3$)]** (0.125 mM) with 2,6-Me$_2$-H$_2$Q (50 equiv; BDFE$_{avg}$ = 66.2 kcal/mol)[10] in DMF at 25 °C. The complex is only partially consumed at the end of the reaction (equilibrium).

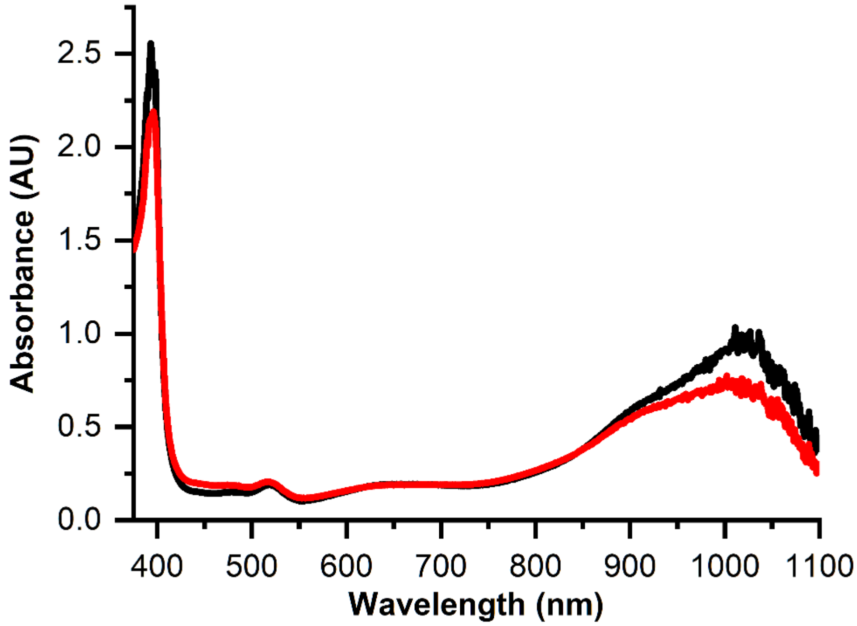

Figure S10. Initial (black trace) and final (red trace) UV-vis spectra for the reaction of **[^{sq}LCu(NEt$_3$)]** (0.125 mM) with 2,6-Cl_2-H_2Q (50 equiv; $BDFE_{avg}$ = 68.9 kcal/mol)[10] in DMF at 25 °C. The complex is only partially consumed at the end of the reaction (equilibrium).

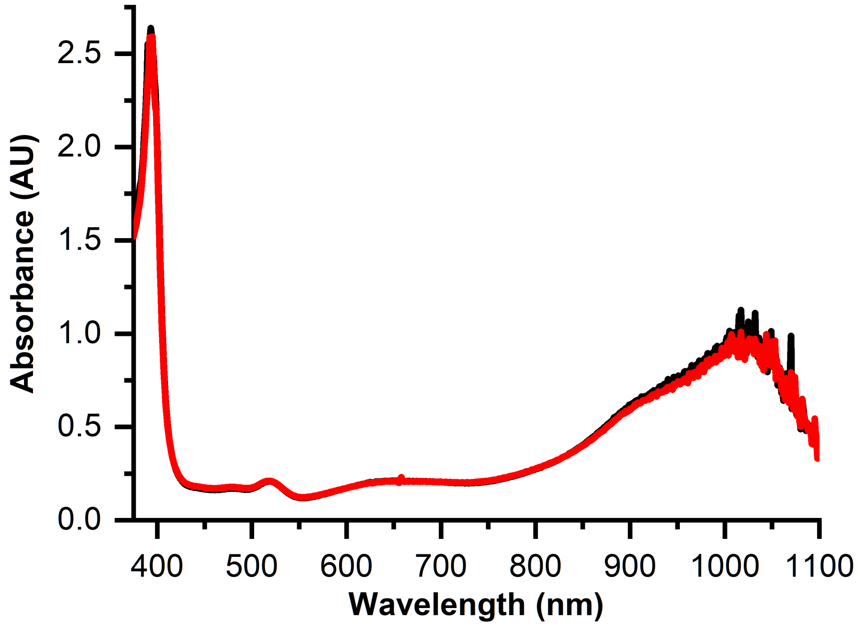

Figure S11. Initial (black trace) and final (red trace) UV-vis spectra for the reaction of **[^{sq}LCu(NEt$_3$)]** (0.125 mM) with 4-MeO-DTBP (25 equiv; BDFE$_{O–H}$ = 71.9 kcal/mol)[10] in DMF at 25 °C. No reaction is observed.

3. Stoichiometry

3.1. Protonation of [^{sq}LCu(NEt$_3$)] with DMF•TfOH

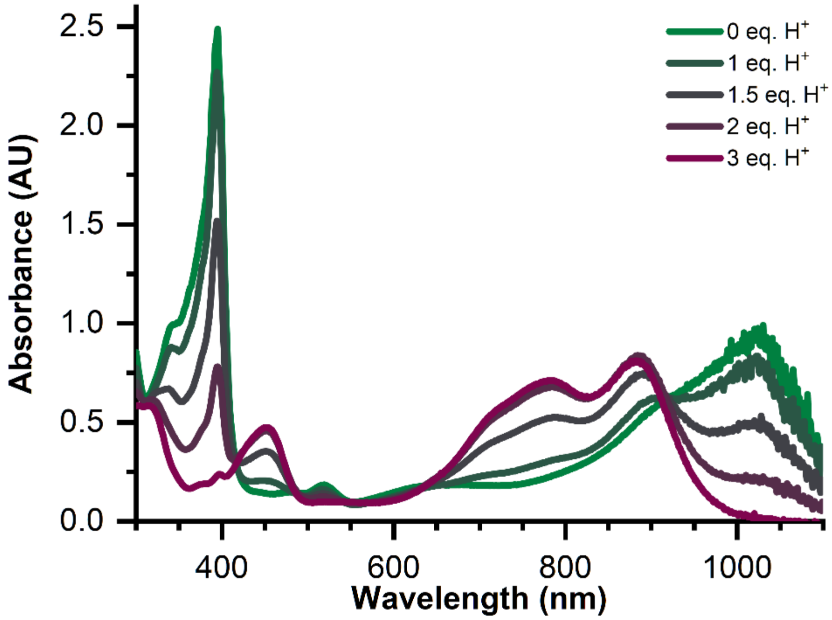

Figure S12. UV-vis spectra for the titration of **[^{sq}LCu(NEt$_3$)]** (0.125 mM) with DMF•TfOH in DMF at 25 °C.

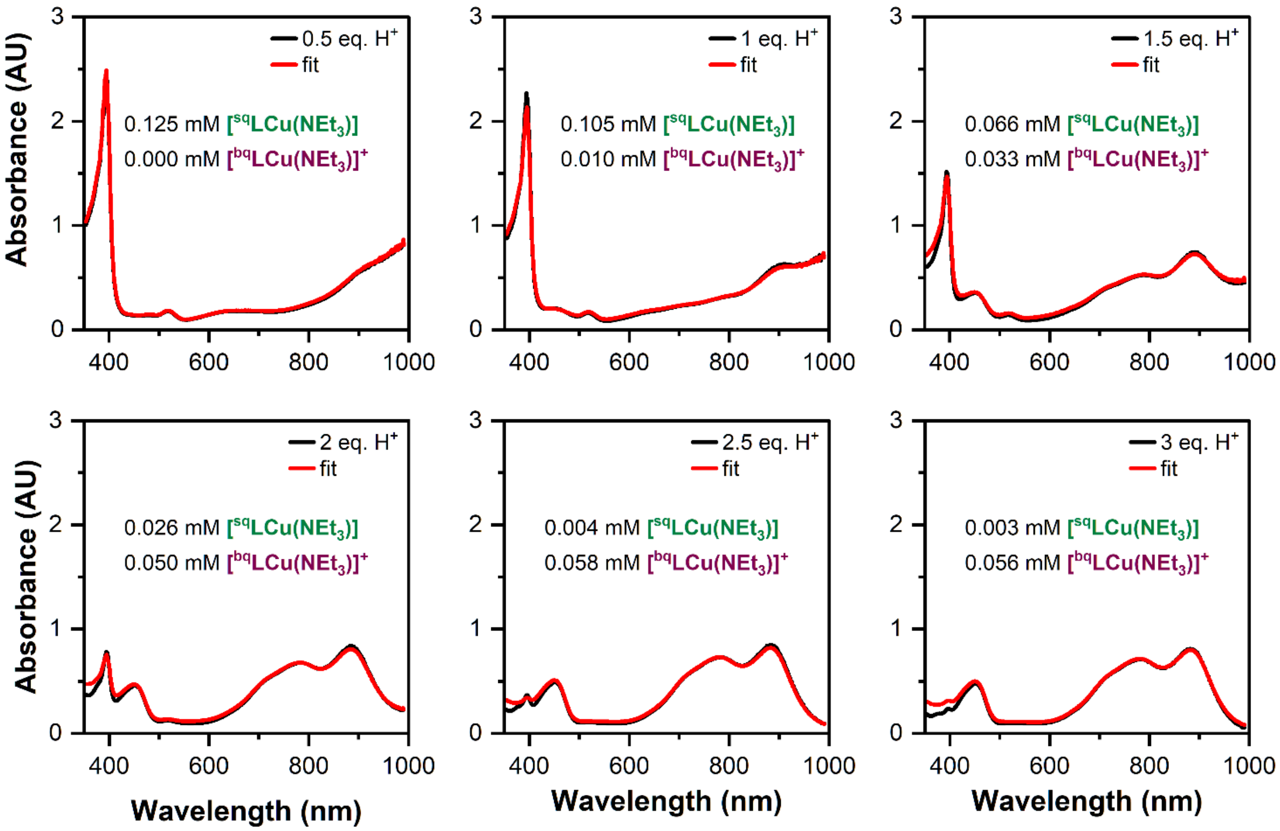

Figure S13. Results of fitting a linear combination of the spectra of pure **[^{sq}LCu(NEt$_3$)]** and **[^{bq}LCu(NEt$_3$)]$^+$** to the spectra from the titration of **[^{sq}LCu(NEt$_3$)]** with DMF•TfOH. The estimated relative concentration of each complex is shown at each titration point. *Note:* Although the identity of the ancillary ligand (X) following protonation is uncertain, it was assumed that the species **[^{sq}LCu(X)]** and **[^{bq}LCu(X)]$^+$** (where X = NEt$_3$, DMF, or TfO$^-$) exhibit UV-vis spectra and molar absorptivities similar to those of **[^{sq}LCu(NEt$_3$)]** and **[^{bq}LCu(NEt$_3$)]$^+$**.

Fitting: The relative concentrations of the observed Cu species **[^{sq}LCu(X)]** and **[^{bq}LCu(X)]$^+$** (where X = NEt$_3$, DMF, or TfO$^-$) at each titration point were estimated by fitting the spectra of the titration mixtures to a linear combination of the spectra of pure **[^{sq}LCu(NEt$_3$)]** and **[^{bq}LCu(NEt$_3$)]$^+$** with known concentration via minimization of the mean absolute error[14] (**Figure S13**). Although the identity of the ancillary ligand (X) following protonation is uncertain, due to likely protonation and displacement of NEt$_3$ (see ^{1}H NMR spectra below), the UV-vis features of **[^{sq}LCu(X)]** and **[^{bq}LCu(X)]$^+$** are assumed to be similar to those of the NEt$_3$-ligated complexes, based on comparisons with related complexes.[1] Fitting was performed using only the 375–1000 nm region of the spectra to exclude noise present at the edges of the instrument's detection range.

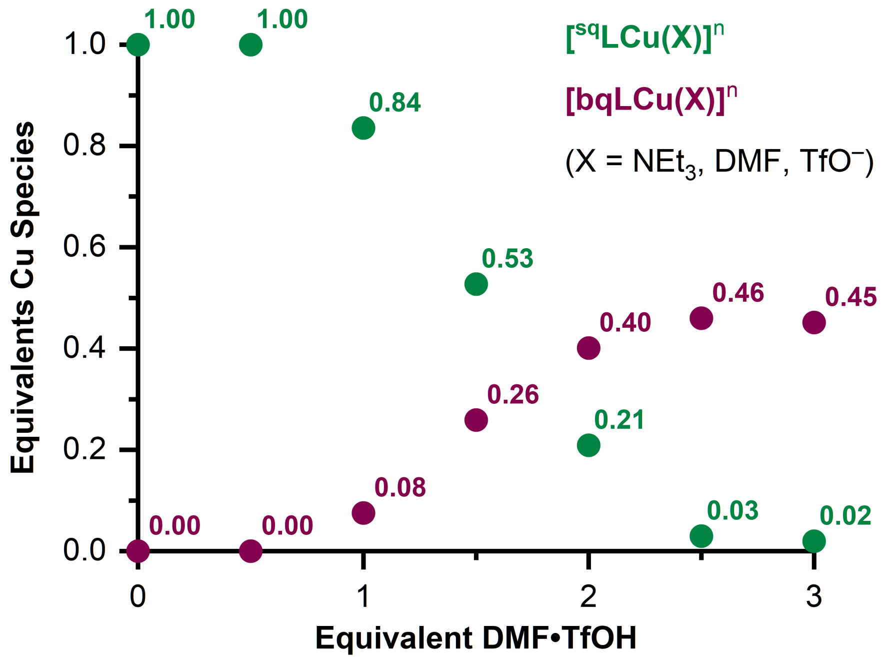

Figure S14. The equivalents of the Cu species formed upon titration of **[^{sq}LCu(NEt$_3$)]** (0.125 mM) with DMF•TfOH. The fitted relative concentrations of **[^{sq}LCu(X)]** and **[^{bq}LCu(X)]**$^+$ from **Figure S13** were converted into equivalents based on the initial concentration of **[^{sq}LCu(NEt$_3$)]** (0.125 mM).

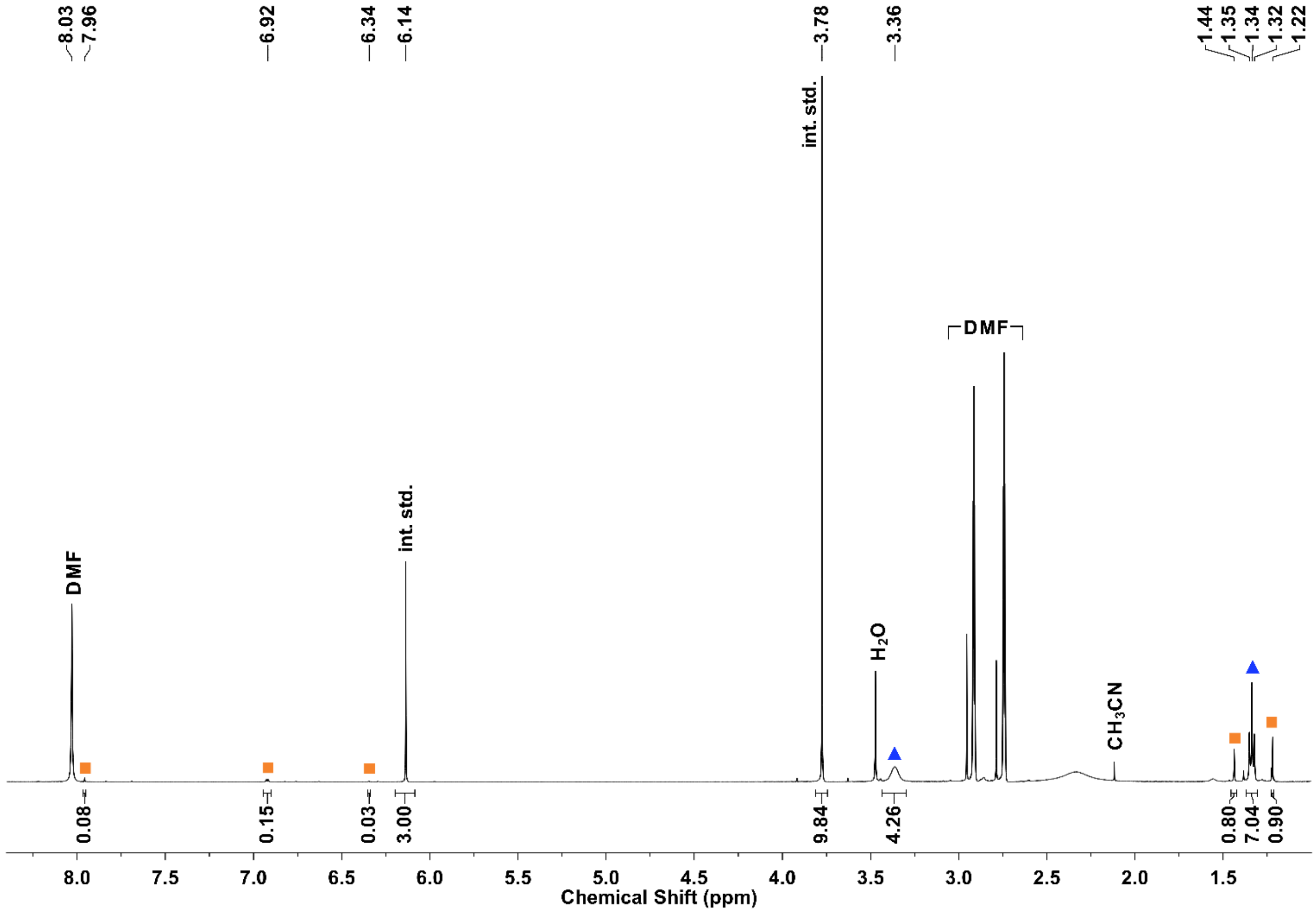

Figure S15. ^{1}H NMR spectrum of **[^{sq}LCu(NEt$_3$)]** (2.50 mM) in DMF-d_7 after addition of 1 equiv DMF•TfOH. Peaks labeled "▲" correspond to $HNEt_3^+$, and those labeled "■" correspond to the cuprous species **[(^{cat}LH$_2$)Cu^{I}(DMF)]**. *Note:* Internal standard (int. std. = 1,3,5-trimethoxybenzene; 3.125 mM).

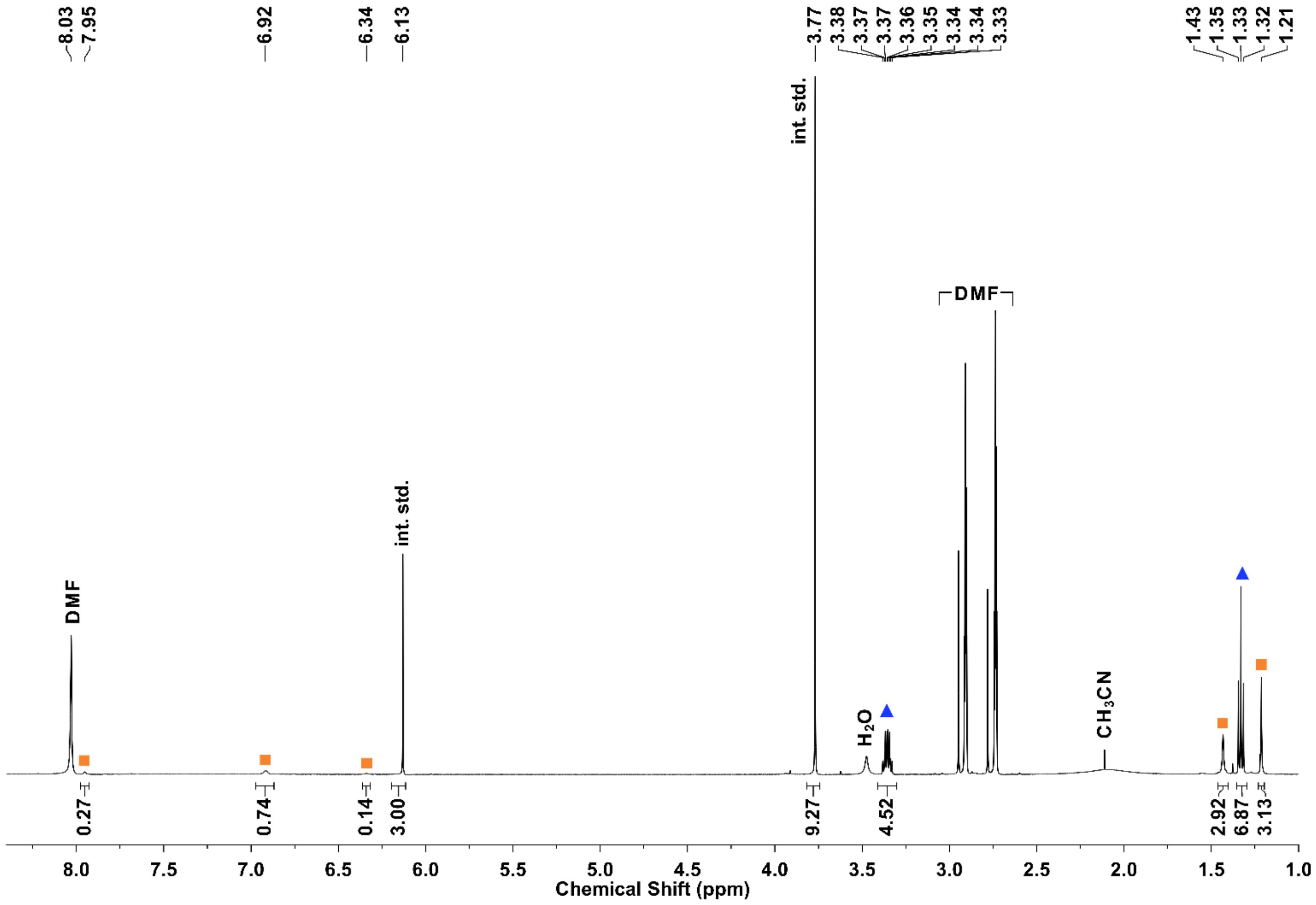

Figure S16. ^{1}H NMR spectrum of **[^{sq}LCu(NEt$_3$)]** (2.50 mM) in DMF-d_7 after addition of 1.5 equiv DMF•TfOH. Peaks labeled "▲" correspond to $HNEt_3^+$, and those labeled "■" correspond to **[(^{cat}LH$_2$)Cu^{I}(DMF)]**. *Note:* Internal standard (int. std. = 1,3,5-trimethoxybenzene; 3.125 mM).

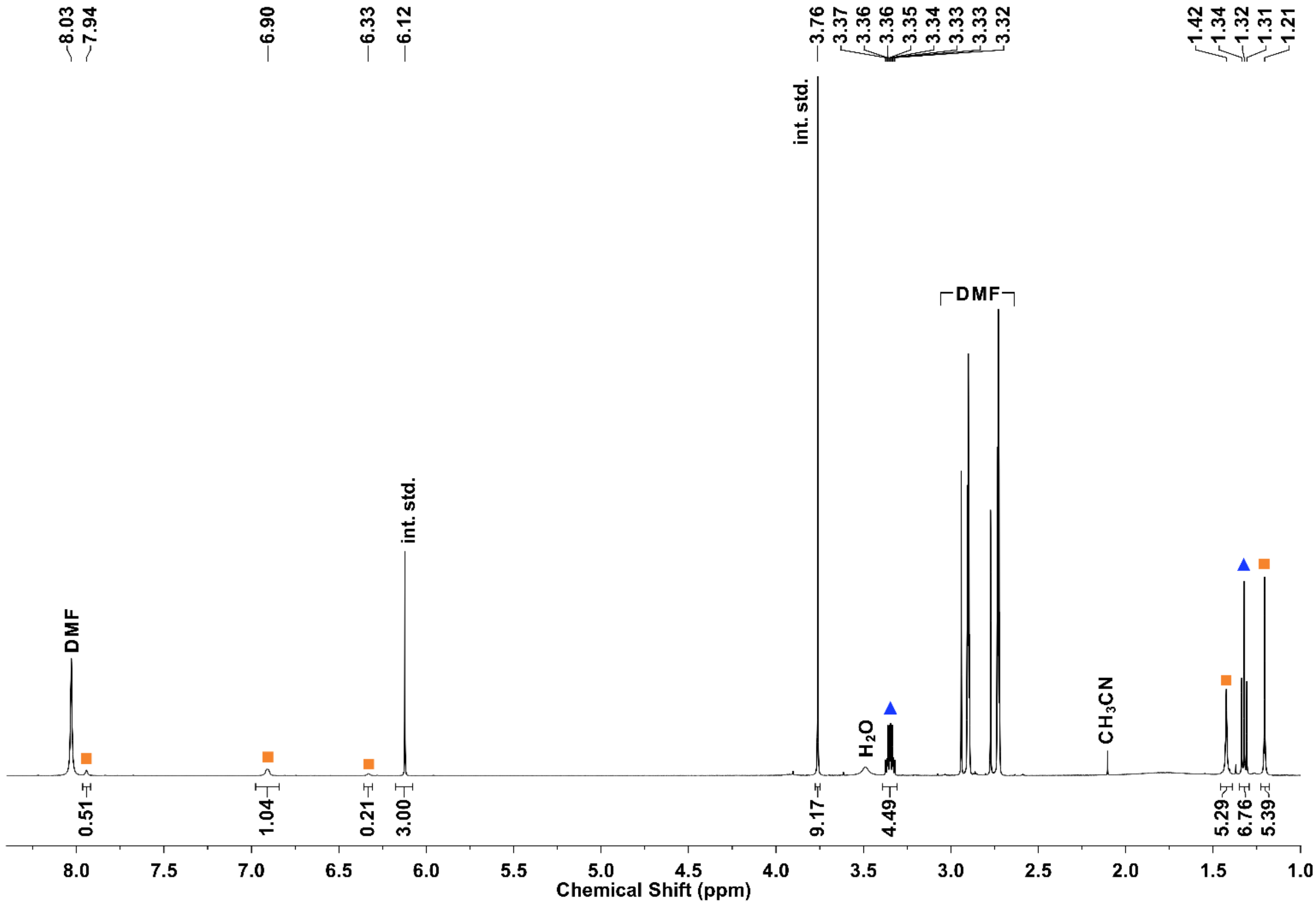

Figure S17. ^{1}H NMR spectrum of **[^{sq}LCu(NEt$_3$)]** (2.50 mM) in DMF-d_7 after addition of 2 equiv DMF•TfOH. Peaks labeled "▲" correspond to $HNEt_3^+$, and those labeled "■" correspond to the cuprous species **[(^{cat}LH$_2$)Cu^I(DMF)]**. *Note:* Internal standard (int. std. = 1,3,5-trimethoxybenzene; 3.125 mM).

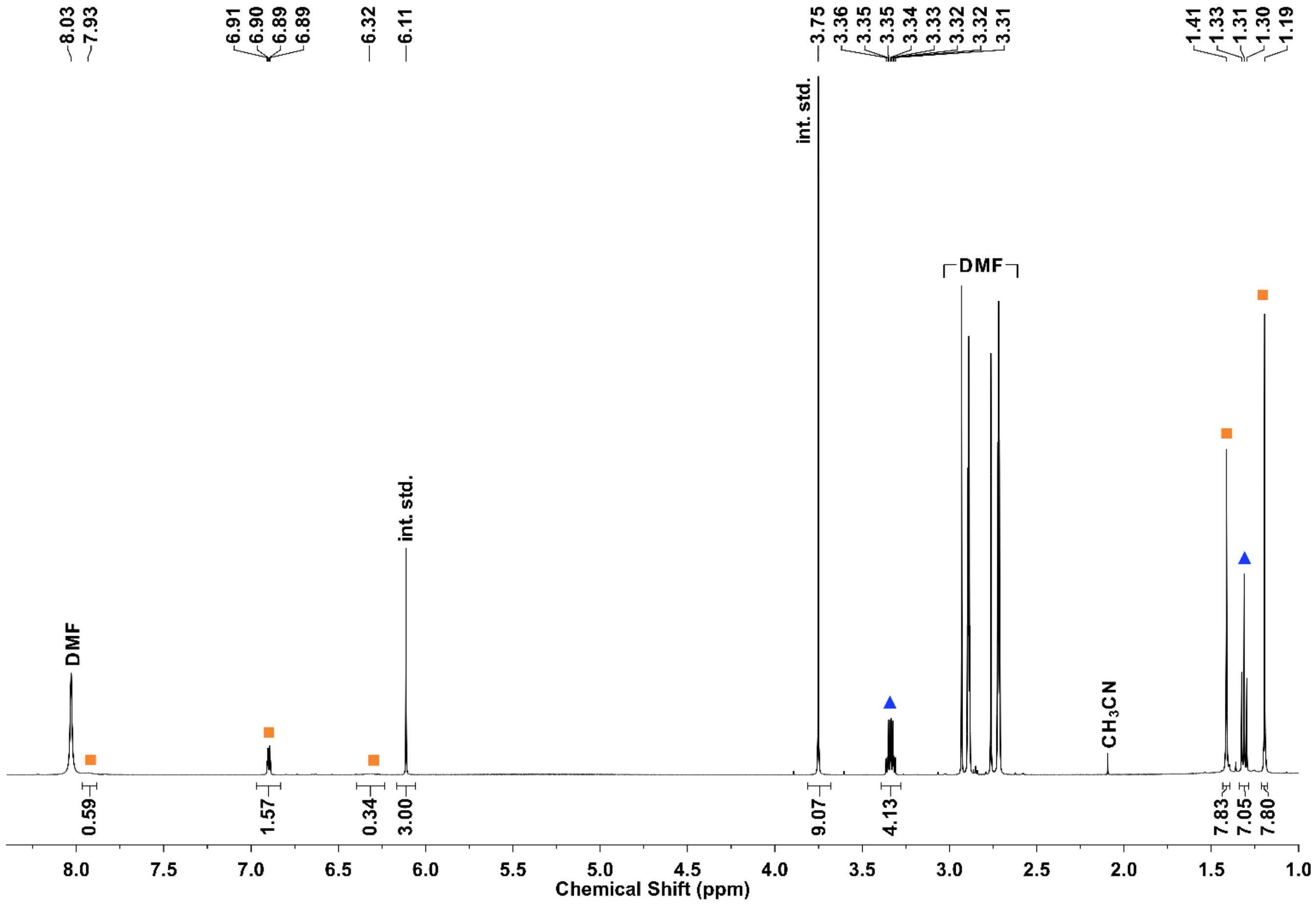

Figure S18. ^{1}H NMR spectrum of **[^{sq}LCu(NEt$_3$)]** (2.50 mM) in DMF-d_7 after addition of 3 equiv DMF•TfOH. Peaks labeled "▲" correspond to $HNEt_3^+$, and those labeled "■" correspond to **[(^{cat}LH$_2$)Cu^{I}(DMF)]**. *Note:* Internal standard (int. std. = 1,3,5-trimethoxybenzene; 3.125 mM).

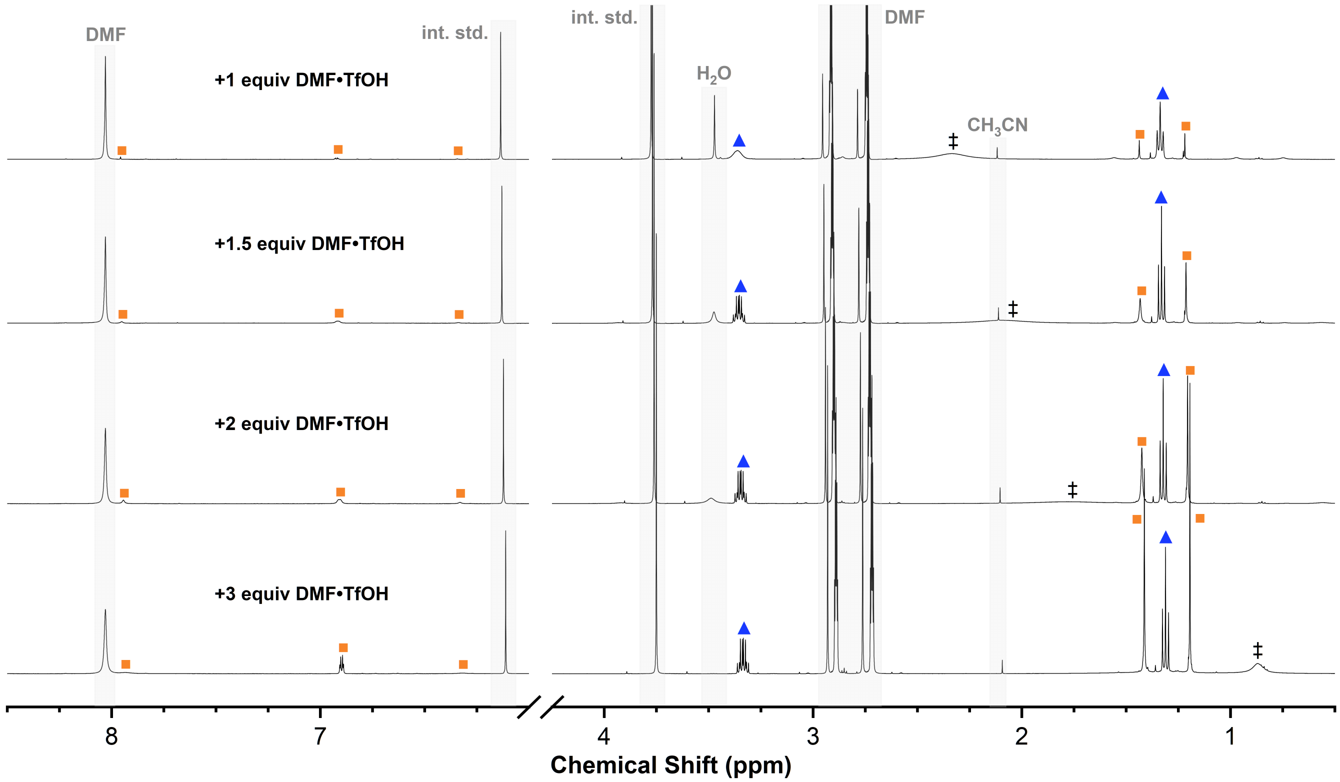

Figure S19. Stacked ^{1}H NMR spectra of **[^{sq}LCu(NEt$_3$)]** in DMF-d_7 after the addition of varying equivalents of DMF•TfOH. The spectra were aligned to the DMF solvent residual peak at 8.03 ppm. Signals corresponding to the internal standard (int. std.; 1,3,5-trimethoxybenzene), DMF solvent residual, water, and acetonitrile are outlined in gray boxes. Peaks labeled "▲" correspond to $HNEt_3^+$, and those labeled "■" correspond to **[(^{cat}LH$_2$)Cu^I(DMF)]**. The broad signals labeled "‡" are assigned to paramagnetic Cu^{II} species.

Table S1. Equivalents of products formed in the protonation of **[^{sq}LCu(NEt$_3$)]** with DMF•TfOH as determined by ^{1}H NMR.

**Equiv. DMF•TfOH**	**Equiv [HNEt$_3^+$]**	**Equiv [(^{cat}LH$_2$)Cu^I(DMF)]**
**1**	0.93	0.05
**1.5**	0.95	0.22
**2**	0.94	0.35
**3**	0.92	0.52

Note: Concentrations were determined by the integration of the corresponding signals relative to the internal standard (1,3,5-trimethoxybenzene; 3.125 mM). The concentrations in the table represent the average concentration determined from all labelled peaks of a given analyte (except for **[(^{cat}LH$_2$)Cu^I(DMF)]**, with which only aryl and *tert*-butyl resonances were considered). Equivalents were calculated based on the concentration of each species relative to the initial concentration of **[^{sq}LCu(NEt$_3$)]** (2.50 mM).

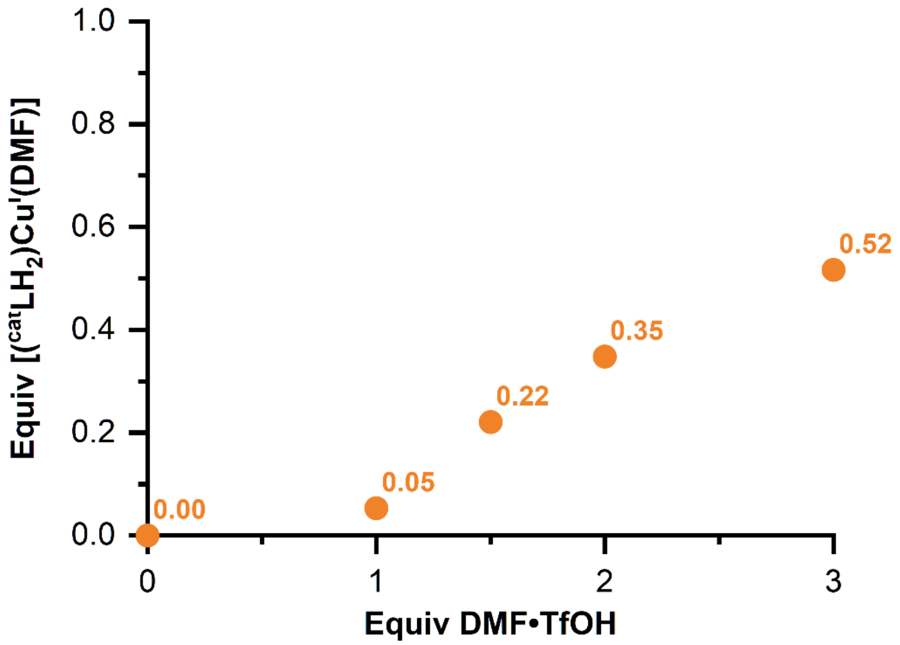

Figure S20. The equivalents of **[(${}^{cat}LH_2$)Cu^I(DMF)]** formed upon titration of **[^{sq}LCu(NEt$_3$)]** with DMF•TfOH as determined by ^{1}H NMR. The concentrations of **[(${}^{cat}LH_2$)Cu^I(DMF)]** at each titration point from **Table S1** were converted into equivalents based on the initial concentration of **[^{sq}LCu(NEt$_3$)]** (2.50 mM).

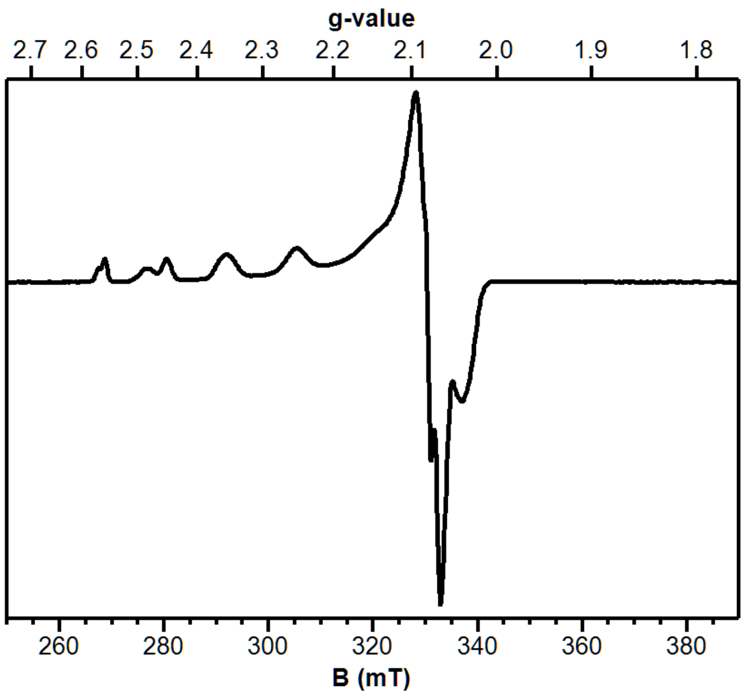

Figure S21. Perpendicular mode EPR spectrum of a frozen DMF solution of **[^{sq}LCu(NEt$_3$)]** (1 mM) after addition of 3 equiv DMF•TfOH. The hyperfine coupling peaks show the presence of multiple Cu^{II} species (T = 11 K).

3.2. Protonation of [^{cat}LCu(NEt$_3$)]$^-$ with DMF•TfOH

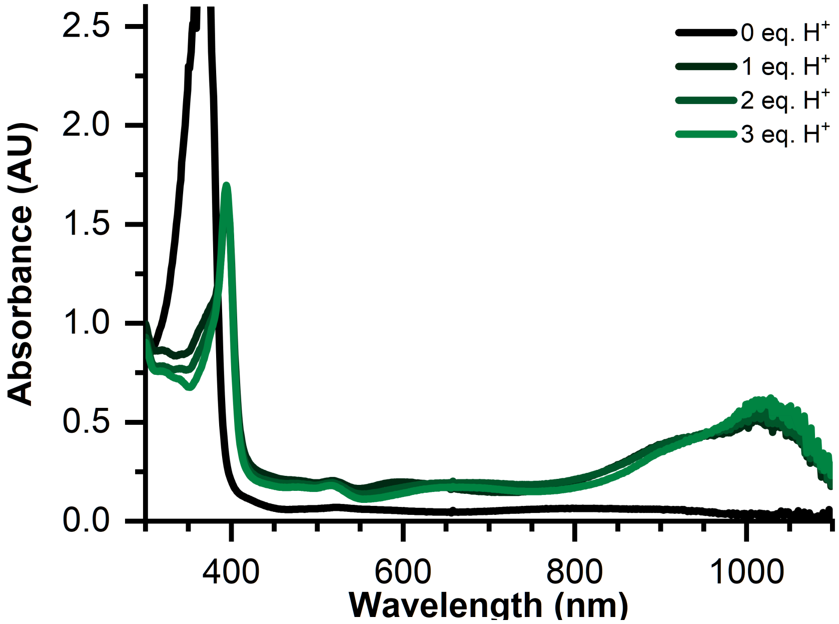

Figure S22. UV-vis spectra for the titration of **[^{cat}LCu(NEt$_3$)]$^-$** (0.125 mM) with DMF•TfOH in DMF at 25 °C.

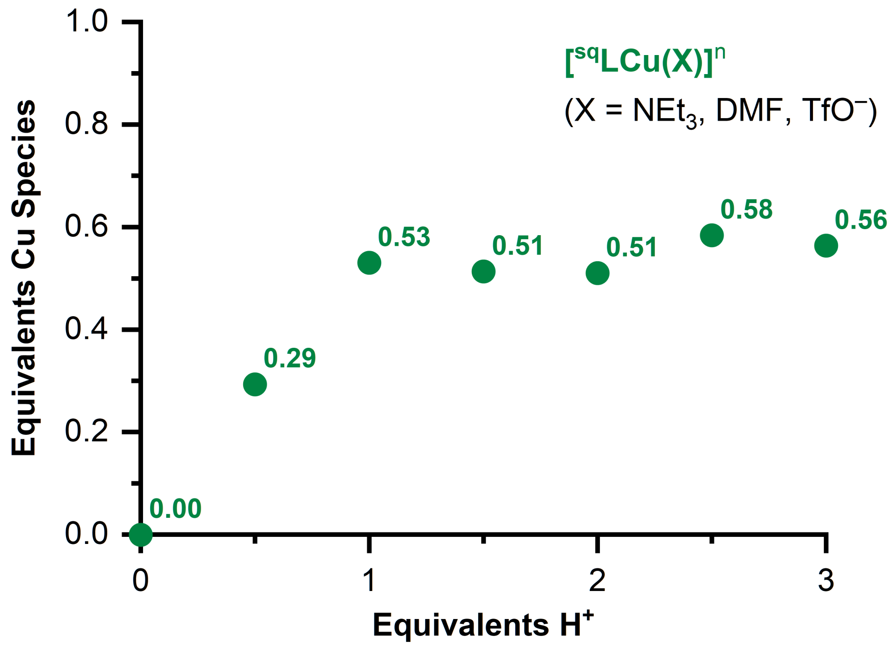

Figure S23. The equivalents of **[^{sq}LCu(X)]^{n}** species formed upon titration of **[^{cat}LCu(NEt$_3$)]$^{-}$** (0.125 mM) with DMF•TfOH, as determined by UV-vis spectroscopy. The concentration of **[^{sq}LCu(X)]^{n}** at each titration point was calculated from the absorbance at 1020 nm (ε = 7540 $M^{-1}cm^{-1}$)[1] and converted to equivalents relative to the initial concentration of **[^{cat}LCu(NEt$_3$)]$^{-}$**. Due to the negligible absorption of **[^{cat}LCu(NEt$_3$)]$^{-}$** at 1020 nm, no baseline correction was applied, except at 0 equiv acid, which was manually set to zero. *Note:* Although the identity of the ancillary ligand (X) following protonation is uncertain, it was assumed that the species **[^{sq}LCu(X)]** (where X = NEt$_3$, DMF, or TfO^{-}) exhibit UV-vis spectra and molar absorptivities similar to those of **[^{sq}LCu(NEt$_3$)]** based on comparisons with related complexes.[1]

3.3. PCET Reaction with TEMPOH

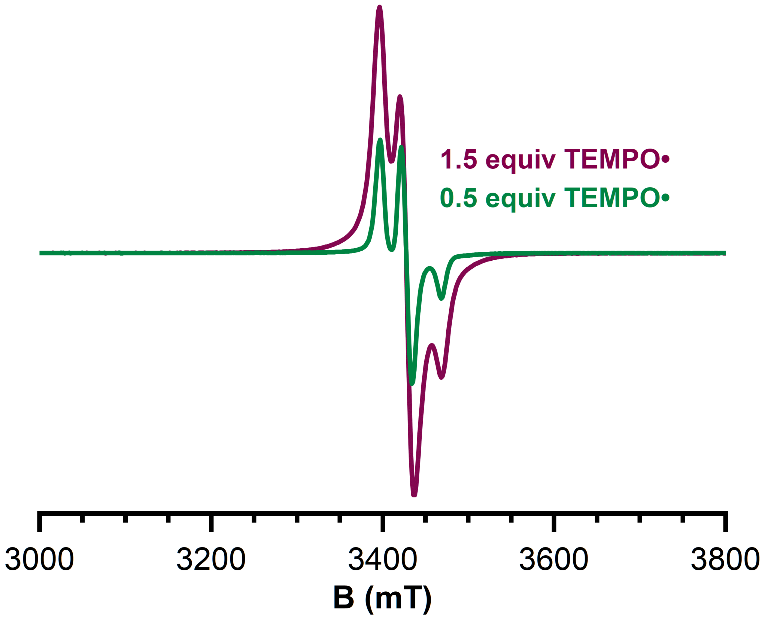

Figure S24. Perpendicular mode EPR spectra of the frozen reaction mixtures (in DMF) of the **[LCu(NEt$_3$)]^n** complexes (1 mM) with TEMPOH (20 equiv). The reaction with **[^{sq}LCu(NEt$_3$)]** produced 0.5 equiv TEMPO• radical (green trace), while the reaction with **[^{bq}LCu(NEt$_3$)]$^+$** (maroon trace) produced 1.5 equiv TEMPO•. The spectra were recorded at 19 K.

3.4. PCET Reaction with 1,4-H$_2$NQ

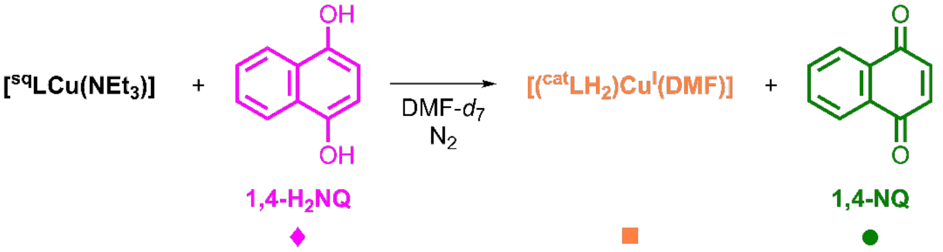

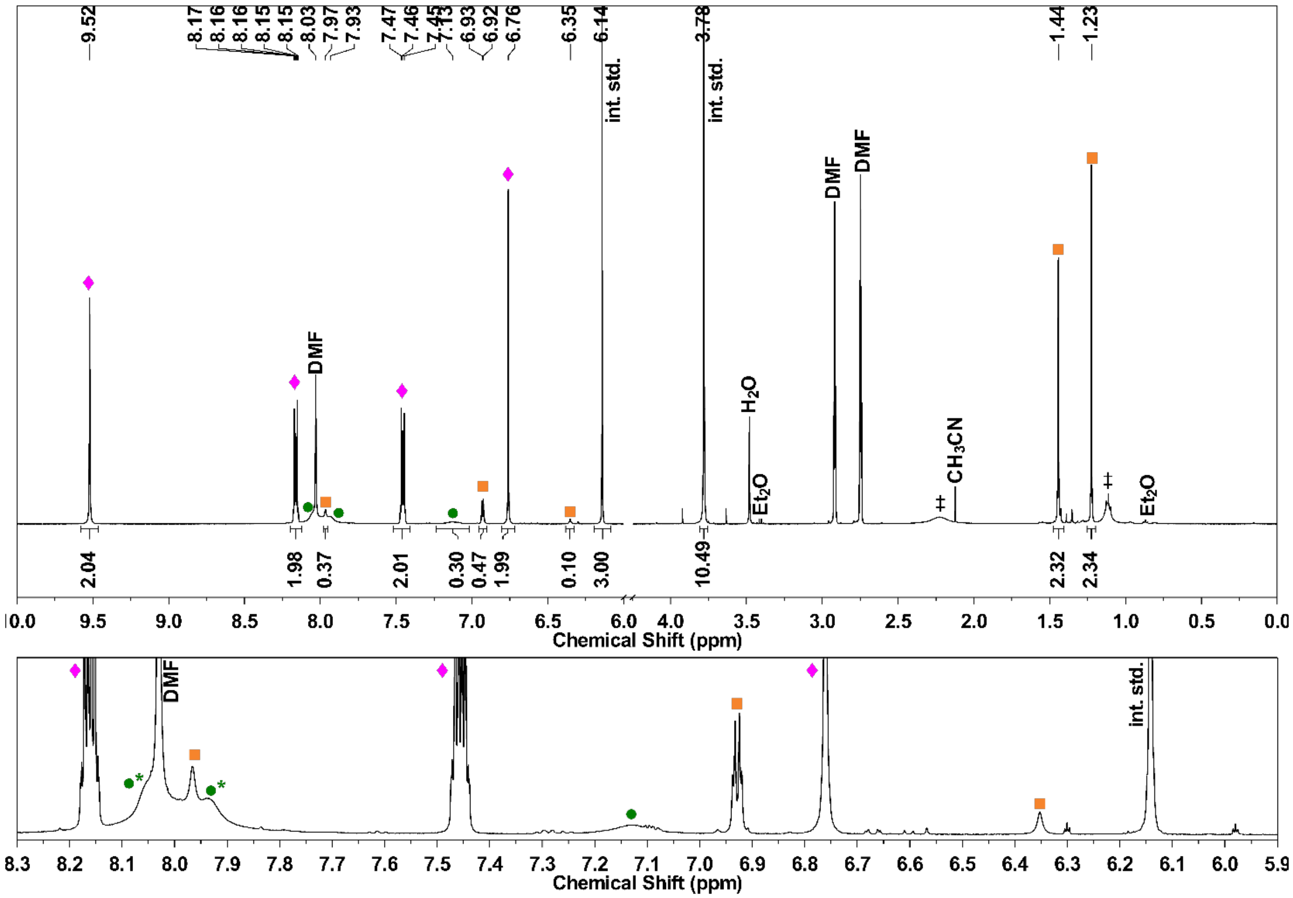

Figure S25. ^{1}H NMR spectrum of the reaction between **[^{sq}LCu(NEt$_3$)]** (2.5 mM) with 1,4-H$_2$NQ (12.5 mM, 5 equiv) in DMF-d_7 (top) and an expansion showing the 8.3–5.9 ppm region (bottom). Peaks labeled "♦" correspond to 1,4-H$_2$NQ; "●" to 1,4-NQ, and "■" to the cuprous species **[(^{cat}LH$_2$)Cu^{I}(NEt$_3$)]**. The broad signals labeled "‡" are assigned to paramagnetic Cu^{II} species. *Note:* The aromatic 1,4-NQ peaks in the 7.95–8.05 ppm region ("●*" in the bottom spectrum) are broad and overlap with the DMF solvent residual signal and the phenolic resonances of **[(^{cat}LH$_2$)Cu^{I}(NEt$_3$)]** and were therefore not used for quantification.

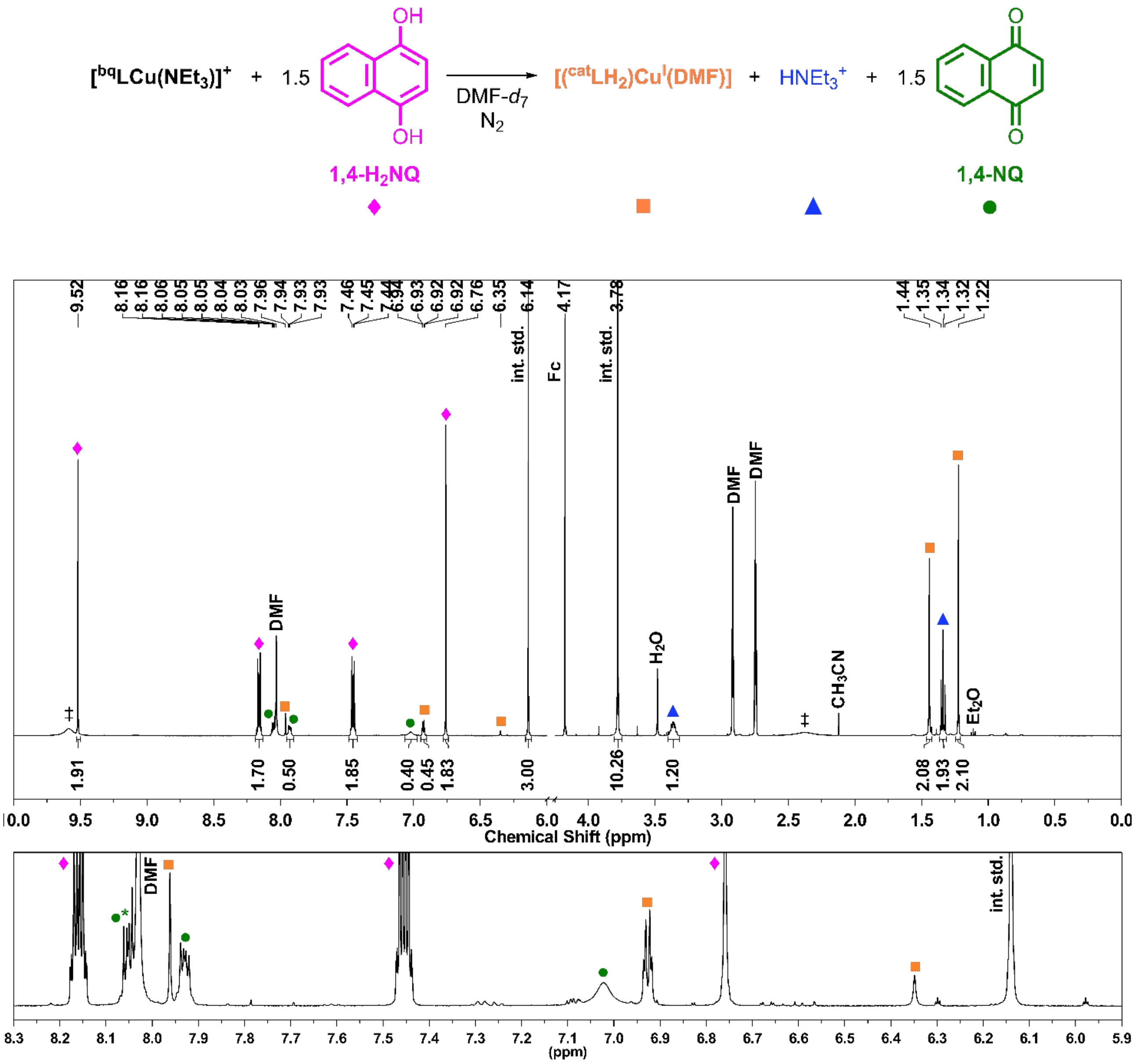

Figure S26. ^{1}H NMR spectrum of the reaction between **[^{bq}LCu(NEt$_3$)]$^+$** (2.5 mM) with 1,4-H_2NQ (12.5 mM, 5 equiv) in DMF-*d*$_7$ (top) and an expansion showing the 8.3–5.9 ppm region (bottom). Peaks labeled "♦" correspond to 1,4-H_2NQ; "●" to 1,4-NQ; "▲" to $HNEt_3^+$, and "■" to the cuprous species **[(^{cat}LH$_2$)Cu^I(DMF)]**. The broad signals labeled "‡" are assigned to paramagnetic Cu^{II} species. *Note:* The aromatic 1,4-NQ peaks at 8.05 ppm ("●*" in the bottom spectrum) overlap with the DMF solvent residual signal and were therefore not used for quantification.

Table S2. Equivalents and yields of products formed per [Cu] in reactions with 1,4-H_2NQ, as determined by ^{1}H NMR spectroscopy.

	**[^{sq}LCu(NEt$_3$)]**		**[^{bq}LCu(NEt$_3$)]$^+$**	
	**Equiv. relative to [Cu]$_0$**	**Yield (%)***	**Equiv. relative to [Cu]$_0$**	**Yield (%)†**
1,4-NQ	0.67	67	1.14	76
$HNEt_3^+$	0	--	0.92	--
[(^{cat}LH$_2$)Cu^I(X)] §	0.58‡	--	0.60‡	--

* based on 2H$^+$/2e$^-$ stoichiometry; † based on 3H$^+$/3e$^-$ stoichiometry; ‡ Only the aryl and *tert*-butyl proton resonances were used for quantification of **[(^{cat}LH$_2$)Cu^I(X)]**. § X = NEt$_3$ and DMF in the reactions with **[^{sq}LCu(NEt$_3$)]** and **[^{bq}LCu(NEt$_3$)]$^+$**, respectively. *Note:* Yields and equivalents are based on the average of two duplicate experiments.

Scheme S2. Reaction scheme and yields for PCET reaction between the **[LCu(NEt$_3$)]^n** complexes and 1,4-H_2NQ.

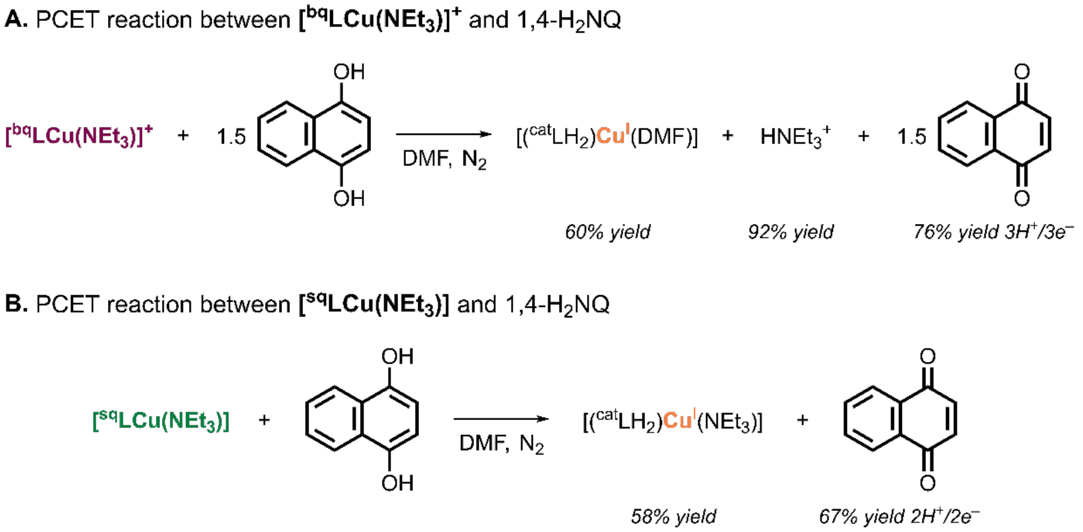

Comment on the protonation state of the cuprous species: Based on the balanced PCET reaction equation, the cuprous product is assigned as **[(^{cat}LH$_2$)Cu^I(X)]** (where X = NEt$_3$ or DMF). However, the ^{1}H NMR spectra of these reaction mixtures suggest that the ligand may be fully protonated (^{cat}LH$_3$), which we attribute to adventitious water in the NMR samples. For clarity and consistency with the main text, the notation **[(^{cat}LH$_2$)Cu^I(X)]** is retained throughout the Supporting Information, with the caveat that the ligand may have a different protonation state under the conditions of the NMR experiments.

4. ^{1}H NMR of the ONO Ligand in the Presence of Cu^I and Triethylamine

To assist in the assignment of the cuprous Cu-ONO species observed in the protonation and PCET NMR experiments, ^{1}H NMR spectra were recorded in DMF-d_7 for the ONO ligand ($^{cat}LH_3$), protonated ONO ligand ([$^{cat}LH_4$](CF_3CO_2)), and each ligand in the presence of Cu^I, with and without NEt_3. Comparison of the spectra reveal only minimal changes in the chemical shifts of the ONO ligand upon addition of Cu^I, or Cu^I and NEt_3.

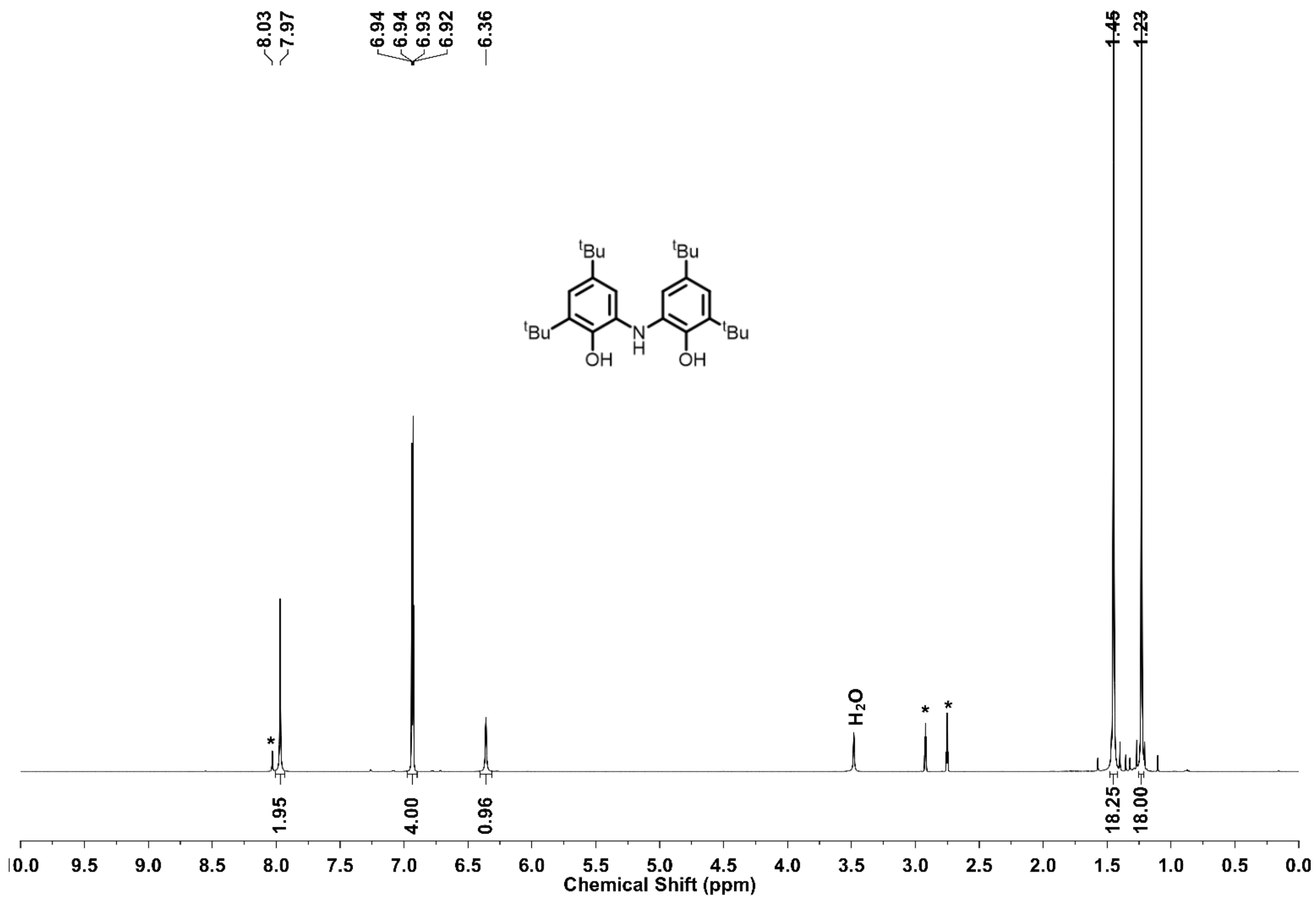

Figure S27. ^{1}H NMR spectrum of the ONO ligand ($^{cat}LH_3$) in DMF-d_7. DMF solvent residual signals are labeled with "*".

Bis(3,5-di-tert-butyl-2-hydroxyphenyl)amine: ^{1}H NMR (500 MHz, DMF-d_7) δ [ppm]: 7.97 (s, 2H), 6.93 (dd, J = 6.3, 2.4 Hz, 4H), 6.36 (s, 1H), 1.45 (s, 18H), 1.23 (s, 18H).

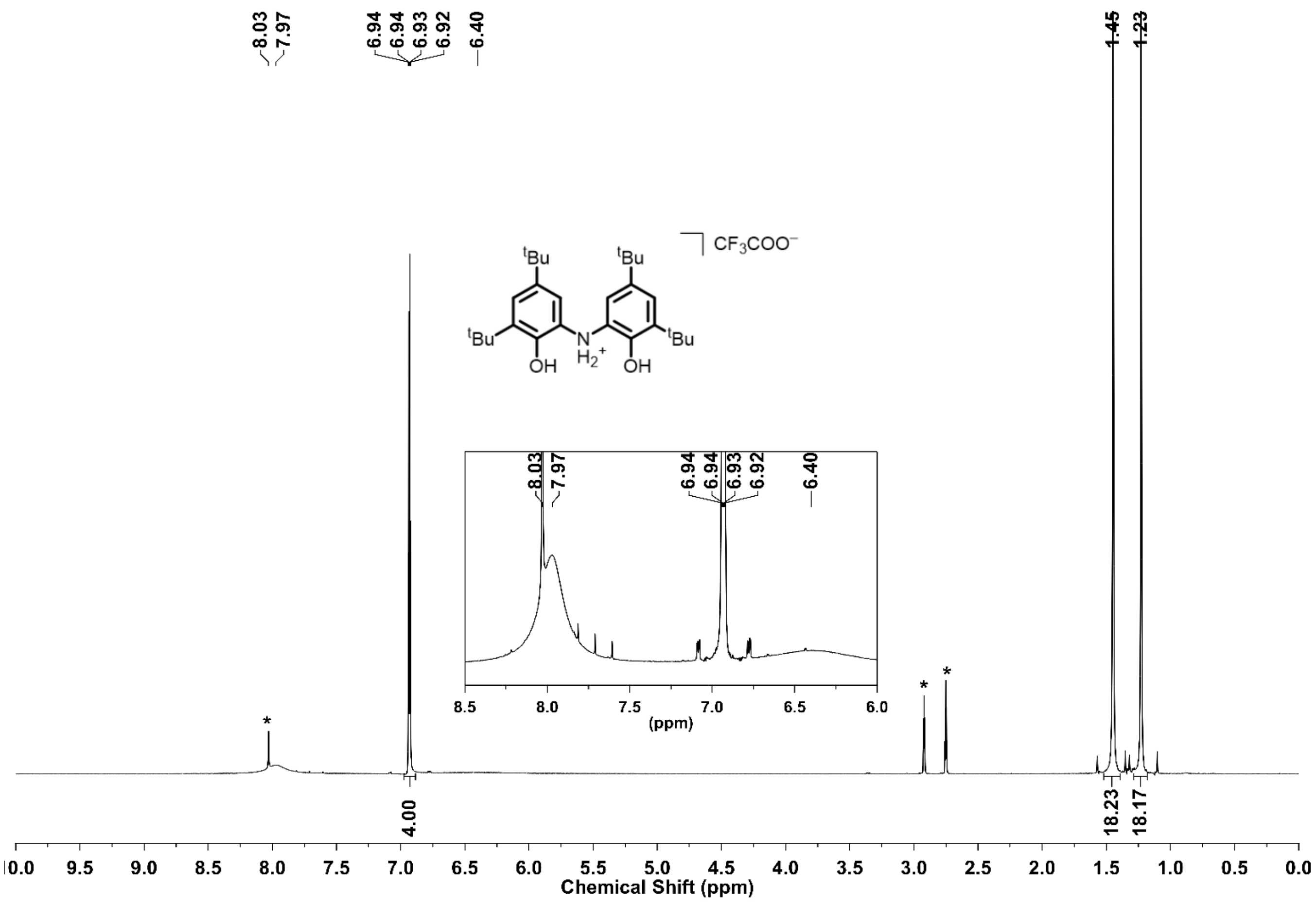

Figure S28. ^1H NMR spectrum of the protonated ONO ligand ($[^{cat}LH_4](CF_3CO_2)$) in DMF-d_7. The exchangeable O–H (7.97 ppm) and N–H (6.40 ppm) signals are significantly broadened, as shown in the zoomed expansion of the 8.5–6.0 ppm region. DMF solvent residual signals are labeled with "*".

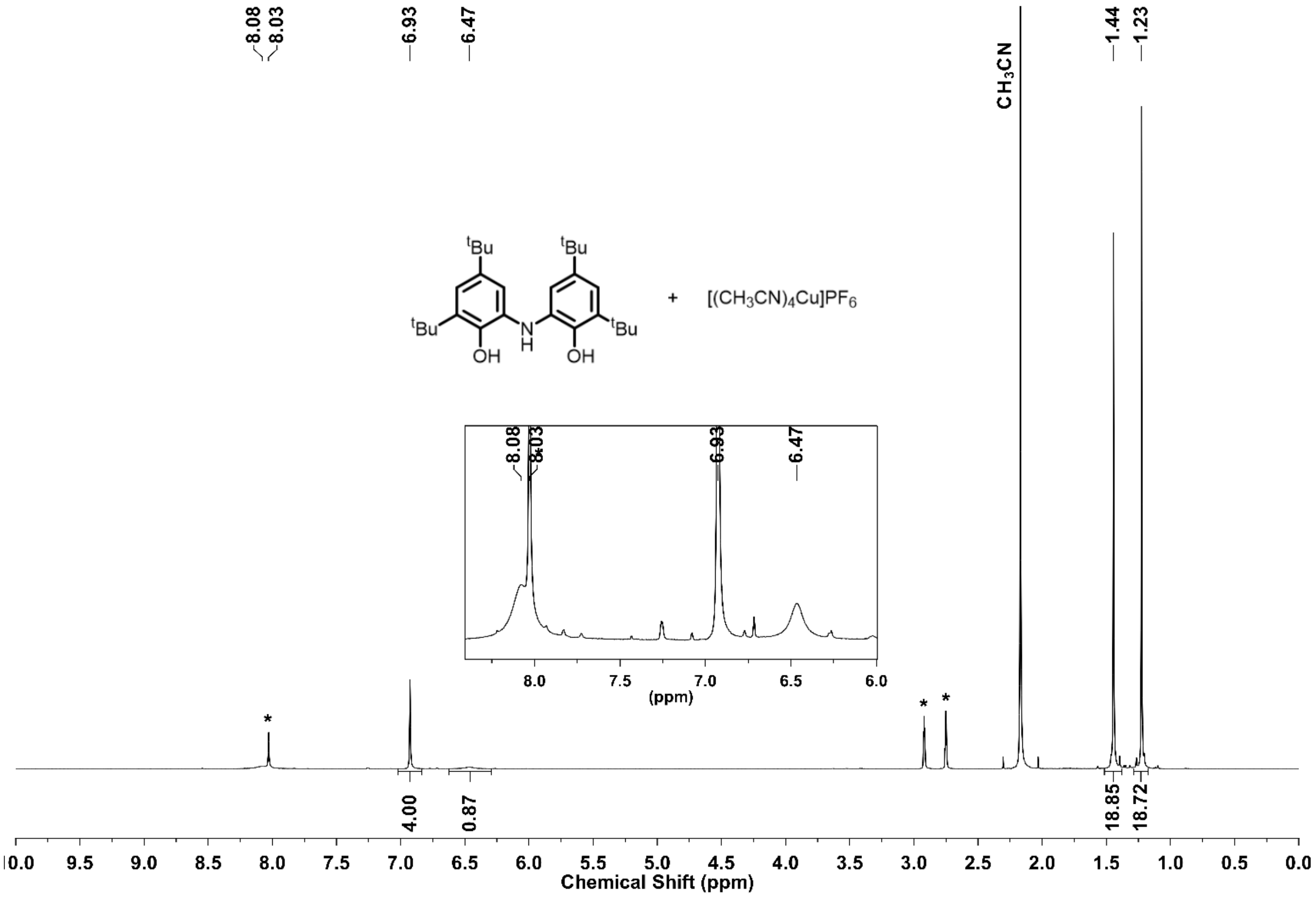

Figure S29. ^{1}H NMR spectrum in DMF-d_7 of the ONO ligand ($^{cat}LH_3$) in the presence of Cu^{I} (from $[(CH_3CN)_4Cu^{I}]PF_6$). The exchangeable O–H (8.08 ppm) and N–H (6.47 ppm) signals are broad, as shown in the zoomed expansion of the 8.6–6.0 ppm region. DMF solvent residual signals are labeled with "*".

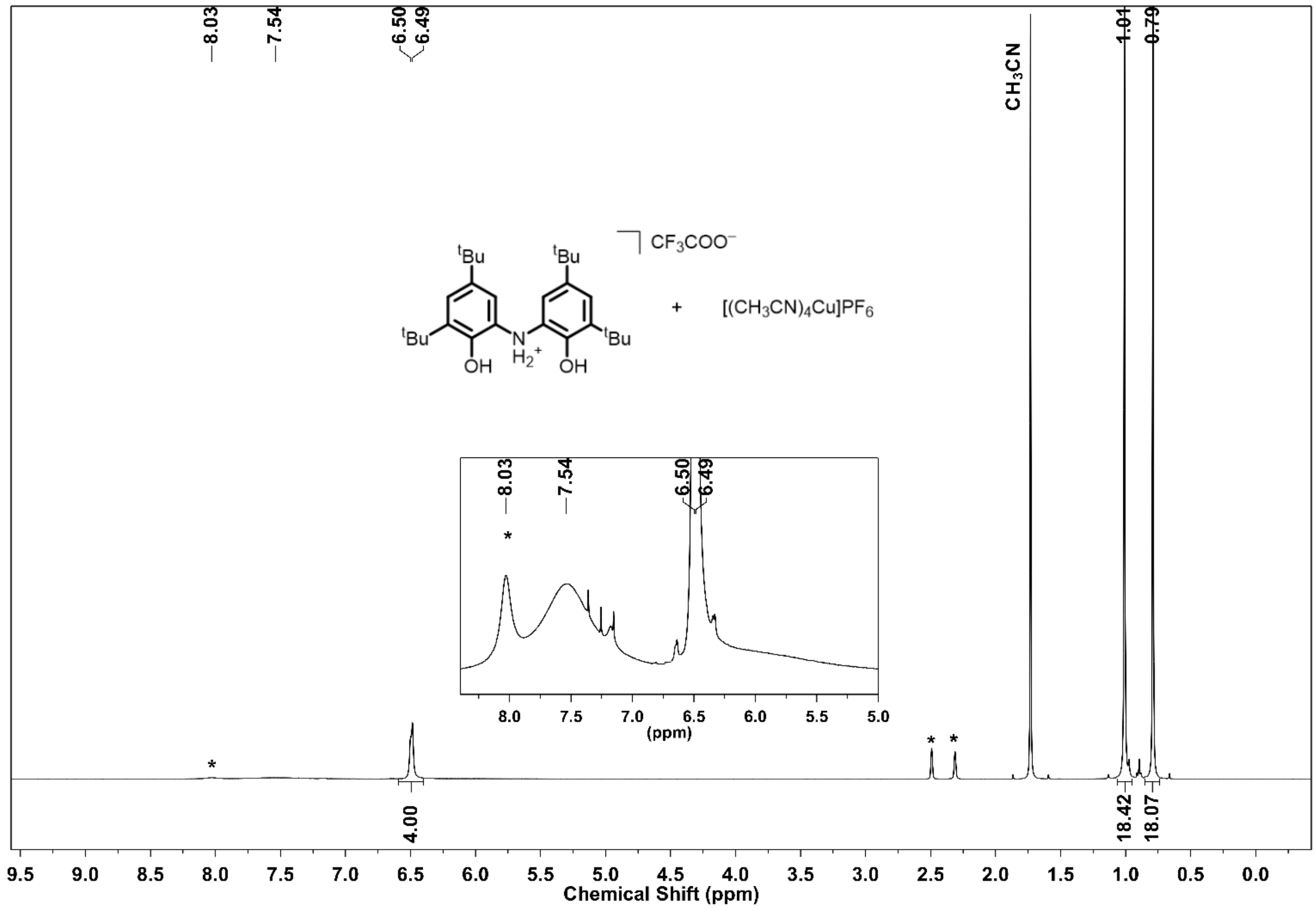

Figure S30. ^{1}H NMR spectrum in DMF-d_7 of the protonated ONO ligand ([^{cat}LH$_4$](CF$_3$CO$_2$)) in the presence of Cu^{I} (from [(CH$_3$CN)$_4$Cu^{I}]PF$_6$). The exchangeable O–H (7.54 ppm) and N–H (unlabeled) signals are significantly broadened, as shown in the zoomed expansion of the 8.6–5.0 ppm region. DMF solvent residual signals are labeled with "*".

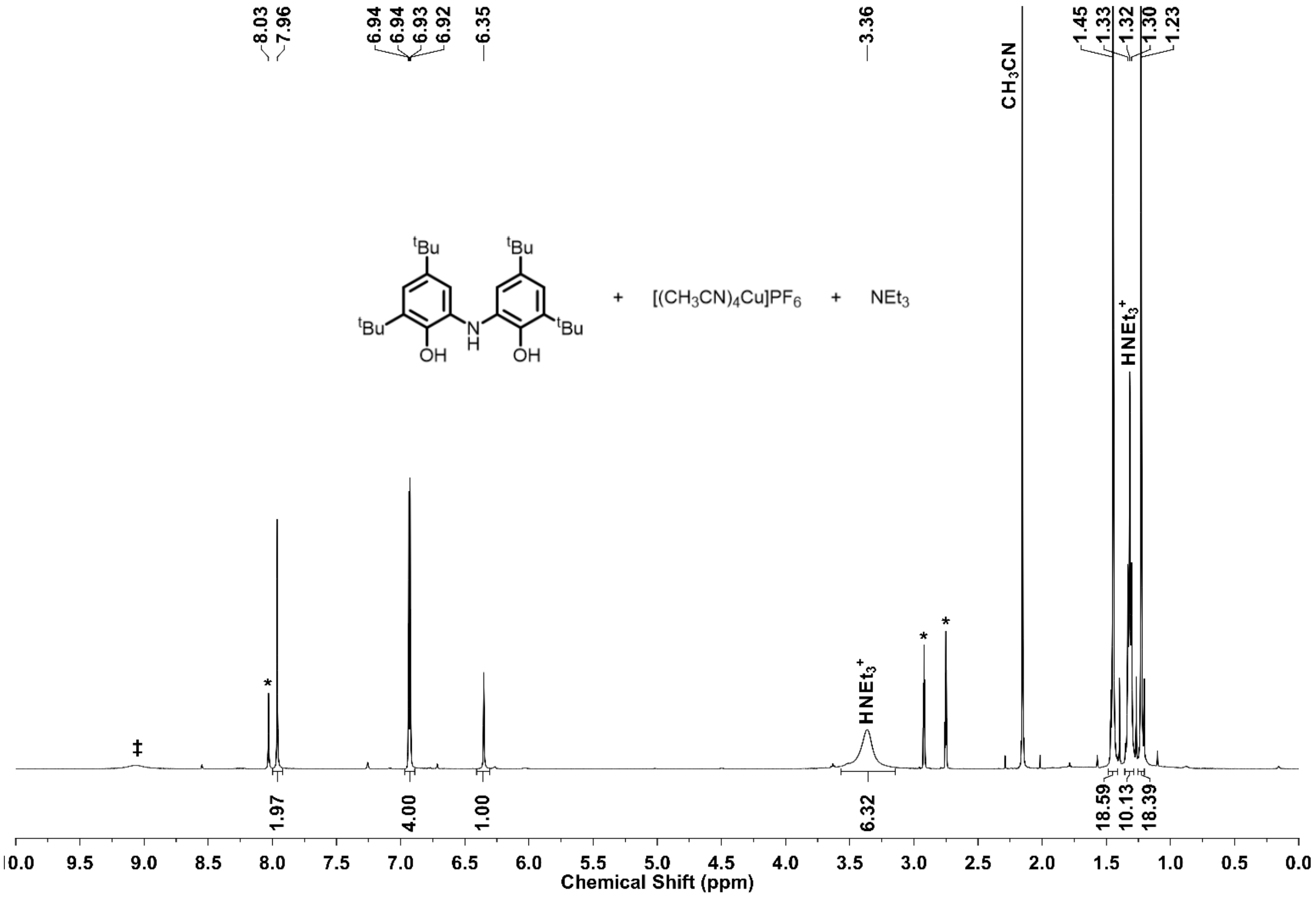

Figure S31. ^{1}H NMR spectrum in DMF-d_7 of the ONO ligand ($^{cat}LH_3$) in the presence of Cu^I (from $[(CH_3CN)_4Cu^I]PF_6$) and NEt_3. DMF solvent residual signals are labeled with "*". The peak labeled "‡" is assigned to a paramagnetic Cu^{II} species (from disproportionation of Cu^I).

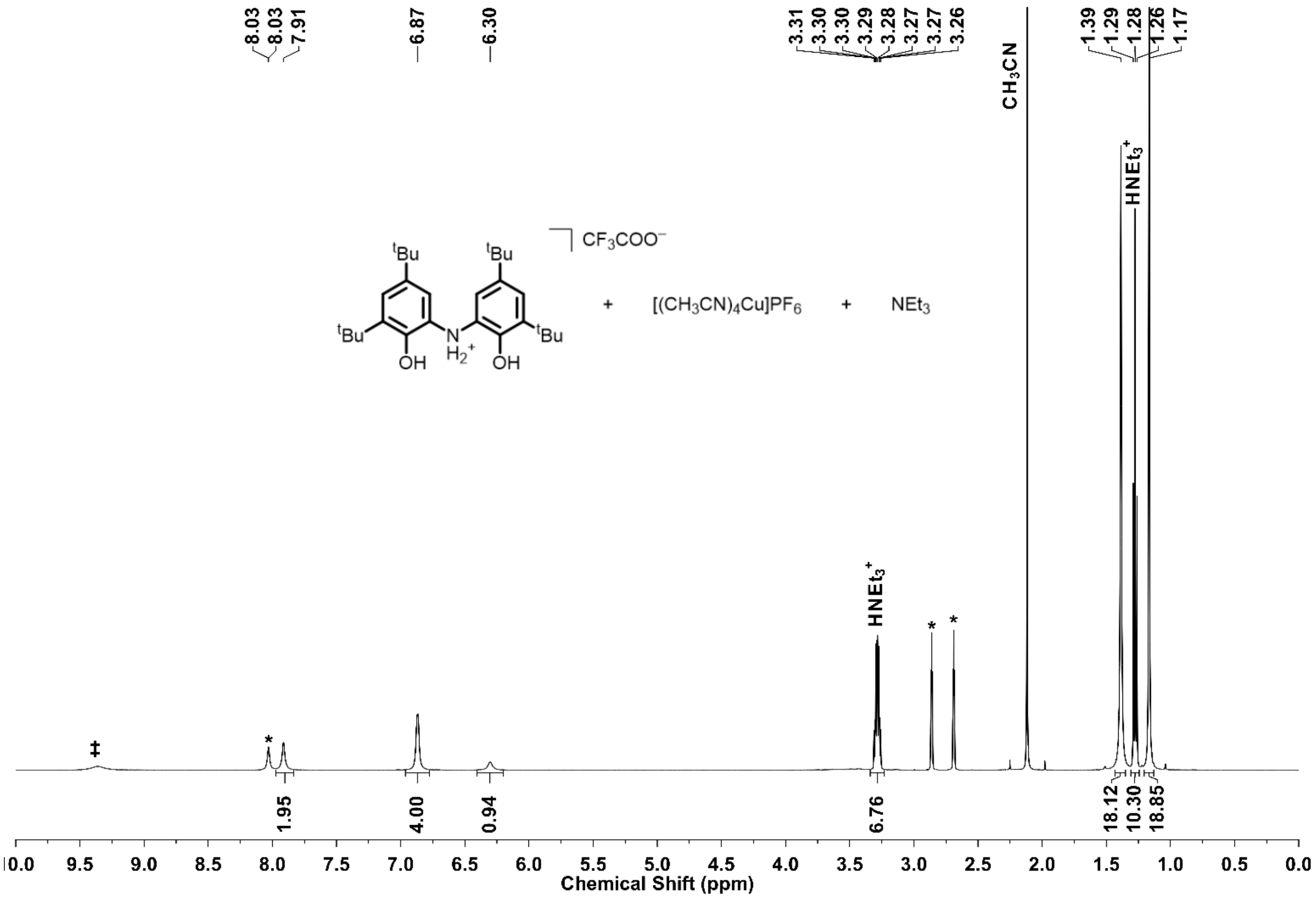

Figure S32. ^{1}H NMR spectrum in DMF-d_7 of the protonated ONO ligand ($[^{cat}LH_4](CF_3CO_2)$) in the presence of Cu^{I} (from $[(CH_3CN)_4Cu^{I}]PF_6$) and NEt_3. The peak labeled "‡" is assigned to a paramagnetic Cu^{II} species (from disproportionation of Cu^{I}). The ligand signals are all appreciably broadened due to the presence of Cu^{II} from disproportionation of Cu^{I}.

5. Estimation of BDFE and pK_a Values

5.1. [^{bq}LCu(NEt$_3$)]$^+$

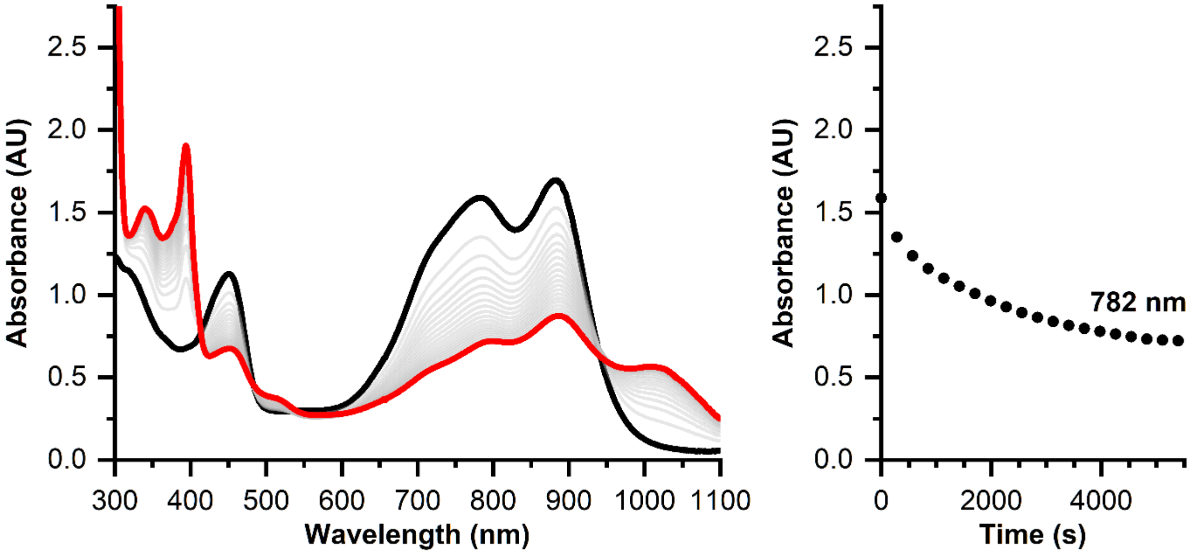

Figure S33. UV-vis spectra (left) and time course of the peak at 782 nm (right) in the reaction between **[^{bq}LCu(NEt$_3$)]$^+$** (0.125 mM) and 4-MeO-DTBP (4.69 mM; 37.5 equiv) in DMF at 25 °C.

The bond dissociated free energy (BDFE) of **[^{bq}LCu(NEt$_3$)]$^+$** was approximated by analysing the equilibrium position of its reaction with 2,6-di-*tert*-butyl-4-methoxyphenol (4-MeO-DTBP; BDFE$_{O-H}$ = 71.9 kcal/mol in DMF)[14] at multiple concentrations in DMF at 25 °C. The equilibrium was treated as reversible, and the equilibrium constant (K_{eq}) was determined using the following expression:

[^{bq}LCu(NEt$_3$)]$^+$ + 4-MeO-DTBP $\rightleftharpoons$ [^{sq}LCu(DMF)] + HNEt$_3^+$ + 4-MeO-DTBP•

$$K_{eq} = \frac{x^2}{(c_i - x)(c_{sub} - x)} \qquad (1)$$

where x is the change in concentration of **[^{bq}LCu(NEt$_3$)]$^+$**, c_i is the initial concentration of **[^{bq}LCu(NEt$_3$)]$^+$**, and c_{sub} is the initial concentration of 4-MeO-DTBP.

Equilibrium constants were determined using a "global" approach by monitoring the absorbance changes at three diagnostic wavelengths (452 nm, ε = 8450 M^{-1}cm^{-1}; 782 nm, ε = 11850 M^{-1}cm^{-1}; 882 nm, ε = 12900 M^{-1}cm^{-1}).[1] A summary of the calculated K_{eq} values is provided in **Table S3**.

Table S3. Equilibrium constants (K_{eq}) for the reaction of **[^{bq}LCu(NEt$_3$)]$^+$** at multiple concentrations of 4-MeO-DTBP in DMF.

Equiv. 4-MeO-DTBP	K_{eq} (452 nm)	K_{eq} (782 nm)	K_{eq} (882 nm)
**12.5**	0.0248	0.0713	0.0440
**37.5**	0.00772	0.0198	0.0126
**50**	0.00365	0.00496	0.00272

From the equilibrium constants, the standard Gibbs free energy change (ΔG°) for the was calculated using the Gibbs free energy equation:

$$\Delta G^\circ = -RT \ln K_{eq} \tag{2}$$

Where R = 1.987×10^{-3} kcal/molK and T = 298 K.

The BDFE of **[^{bq}LCu(NEt$_3$)]$^+$** was then estimated by subtracting the calculated ΔG° from the known BDFE of 4-MeO-DTBP (71.9 kcal/mol in DMF):[10]

$$\mathrm{BDFE_{complex}} = \mathrm{BDFE_{phenol}} - \Delta G^\circ \tag{3}$$

With the BDFE values of **[^{bq}LCu(NEt$_3$)]$^+$**, the corresponding pK_a values for the protonation of **[^{sq}LCu(NEt$_3$)]** were derived from the BDFEs using the Bordwell equation:

$$\mathrm{p}K_\mathrm{a} = \mathrm{BDFE} - 23.06E_{1/2} - C_G \tag{4}$$

where $E_{1/2}$ is the redox potential of **[^{bq}LCu(NEt$_3$)]$^+$** (−0.4 V vs. Fc$^{+/0}$ in DMF),[1] and C_G is a solvent-dependent constant (67.6 kcal/mol for DMF).[16] A summary of the calculated ΔG°, BDFE, and pK_a values is shown in **Table S4**.

Table S4. Averaged standard Gibbs free energy change (ΔG°) and bond dissociation free energy (BDFE) values for the reductive protonation of **[^{bq}LCu(NEt$_3$)]$^+$** with 4-MeO-DTBP and the corresponding pK_a values for the protonation of **[^{sq}LCu(NEt$_3$)]**.

Wavelength	Avg. ΔG° (kcal/mol)	Avg. BDFE (kcal/mol)	Avg. pK_a
**452**	2.8 ± 0.5	69.1 ± 0.5	7.8 ± 0.3
**782**	2.3 ± 0.6	69.6 ± 0.6	8.2 ± 0.5
**882**	2.6 ± 0.7	69.3 ± 0.7	7.9 ± 0.5
**Overall Average**	**2.6 ± 0.6**	**69.3 ± 0.6**	**8.0 ± 0.4**

The overall average BDFE for the reductive protonation of **[^{bq}LCu(NEt$_3$)]$^+$** was determined to be 69.3 ± 0.6 kcal/mol, corresponding to a pK_a of 8.1 ± 0.4 for the protonation of **[^{sq}LCu(NEt$_3$)]**.

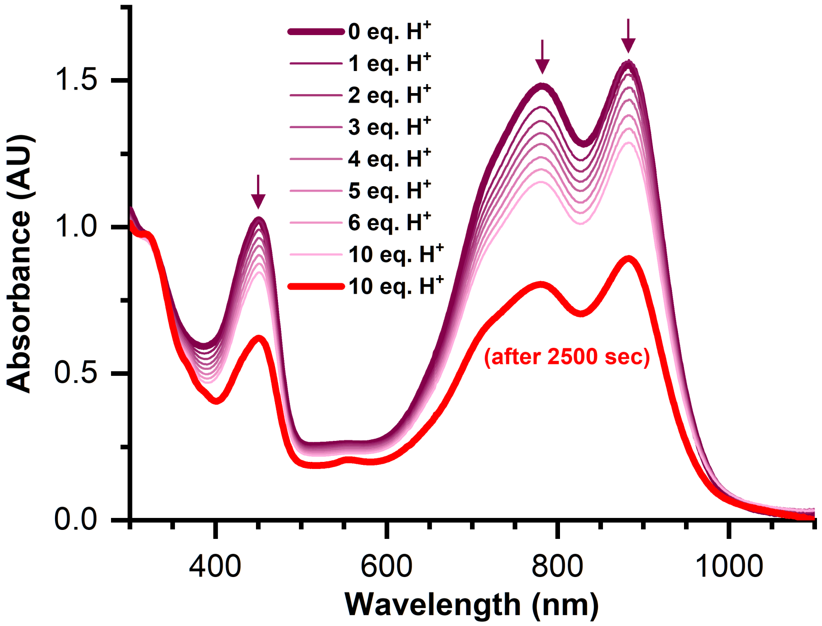

Figure S34. UV-vis spectra for the titration of **$[^{bq}LCu(NEt_3)]^+$** (0.125 mM) with DMF•TfOH (10 equiv) in DMF at 25 °C. The bold red trace corresponds to the spectrum recorded 2500 s after addition of the cumulative addition of 10 equiv DMF•TfOH.

The protonation of **$[^{bq}LCu(NEt_3)]^+$** with DMF•TfOH was monitored by UV-vis spectroscopy, where titration with 10 equiv acid resulted in gradual decay of the complex. Using the reported pK_a of DMF•TfOH in acetonitrile (6.1)[15] and the correlation $pK_a(\mathrm{DMF}) = 0.91 \cdot pK_a(\mathrm{acetonitrile}) - 7.6$,[16] its estimated p$K_a$ in DMF is −2.4. Based on these data, the pK_a **$[^{bq}LCu(NEt_3)]^+$** is qualitatively assigned as ≲−3, corresponding to a reduction potential of ≳0.24 V vs. $Fc^{+/0}$ for the protonated species **$[^{bq}LCu(NEt_3)(H)]^{2+}$**.

Scheme S3. Square scheme for the reductive protonation of **$[^{bq}LCu(NEt_3)]^+$**.

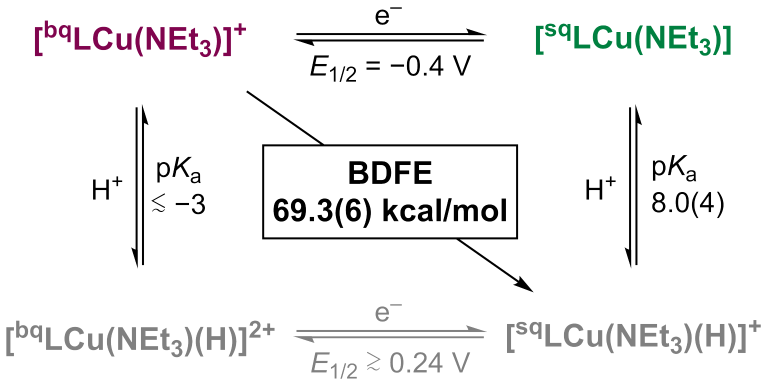

5.2. [^{sq}LCu(NEt$_3$)]

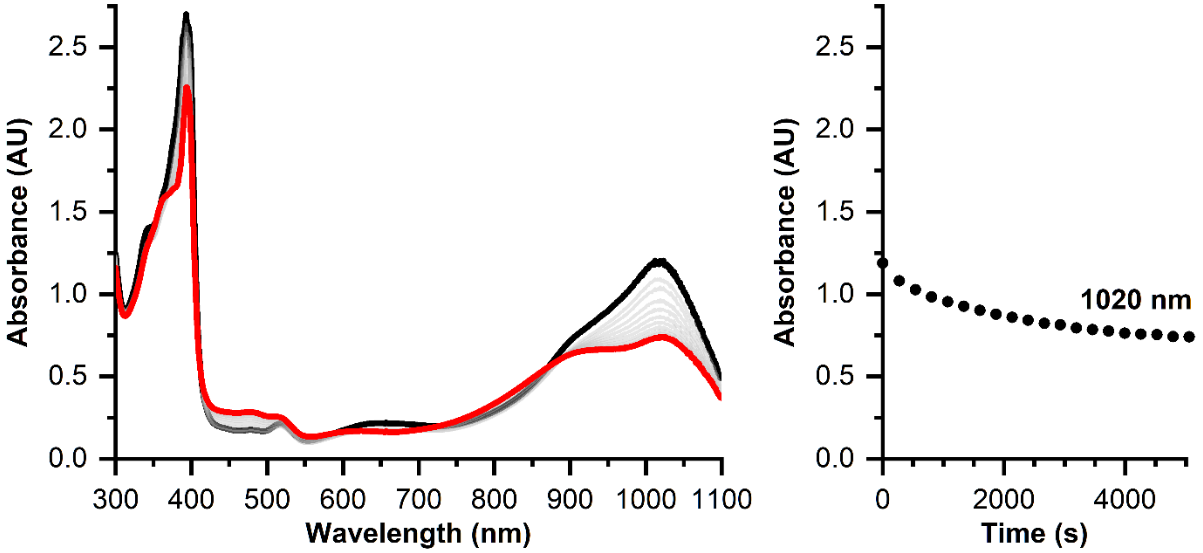

Figure S35. UV-vis spectra (left) and time course of the peak at 1020 nm (right) in the reaction between **[^{sq}LCu(NEt$_3$)]** (0.125 mM) and TEMPOH (6.25 mM; 50 equiv) in DMF at 25 °C.

The BDFE of **[^{sq}LCu(NEt$_3$)]** and pK_a of **[^{cat}LCu(NEt$_3$)]$^-$** were approximated using a similar analysis described above for **[^{bq}LCu(NEt$_3$)]$^+$** by analysing the equilibrium position of the reaction of **[^{sq}LCu(NEt$_3$)]** with 2,2,6,6-tetramethylpiperidin-1-ol (TEMPOH; BDFE$_{O-H}$ = 65.7 kcal/mol in DMF)[1] at multiple concentrations in DMF at 25 °C. Equilibrium constants were determined by monitoring the absorbance changes at 1020 nm (ε = 7540 M^{-1}cm^{-1}).[1] A summary of the calculated K_{eq}, ΔG°, BDFE, and pK_a values is provided in **Table S5**. The reaction between **[^{sq}LCu(NEt$_3$)]** was assumed to be a reversible equilibrium as follows:

$$[^{sq}LCu(NEt_3)] + TEMPOH \rightleftharpoons [(^{cat}LH)Cu(NEt_3)] + TEMPO\bullet$$

Table S5. Equilibrium constants (K_{eq}), standard Gibbs free energy change (ΔG°), and bond dissociation free energy (BDFE) values for the reductive protonation of **[^{sq}LCu(NEt$_3$)]** with TEMPOH in DMF and the corresponding pK_a values for the protonation of **[^{cat}LCu(NEt$_3$)]$^-$**.

Equiv. TEMPOH	K_{eq}	ΔG° (kcal/mol)	BDFE (kcal/mol)	pK_a
**50**	0.00535	3.1	62.6	14.5
**100**	0.00170	3.8	61.9	14.0
**150**	0.000887	4.2	61.5	13.8
**Overall Average**		**3.7 ± 0.5**	**62.0 ± 0.5**	**14.1 ± 0.3**

Scheme S4. Square scheme for the reductive protonation of **[^{sq}LCu(NEt$_3$)]**.

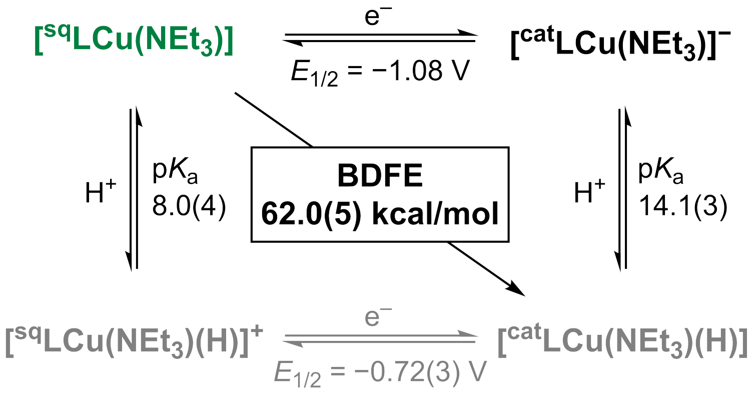

5.3. [^{cat}LCu(NEt$_3$)]$^-$

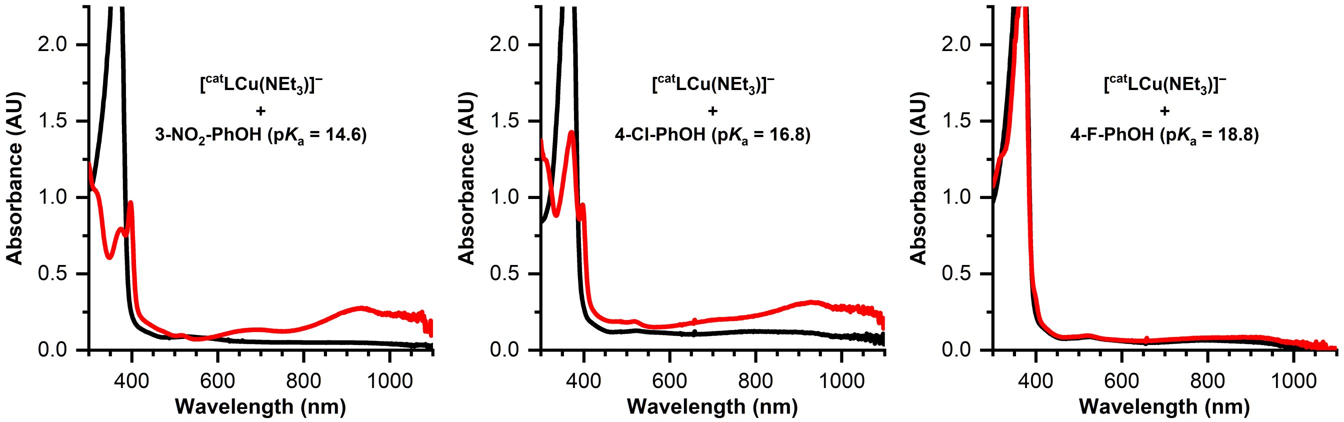

Figure S36. Representative UV-vis spectra of the protonation of **[^{cat}LCu(NEt$_3$)]$^-$** with phenols (10 equiv) of varying acidity to qualitatively assess the pK_a of the complex. The pK_a values of the phenols are taken from literature values in DMF or otherwise converted from reported values in DMSO.[3]

Protonation-induced disproportionation is observed in the reactions with 3-NO_2-PhOH and 4-Cl-PhOh, however no reaction is observed with 4-F-PhOH. These results suggest that the pK_a of **[^{cat}LCu(NEt$_3$)]$^-$** lies between 13–16, consistent with the pK_a value calculated from the Bordwell equation above.

5.4. $[^{bq}LCu(tmpda)]^+$

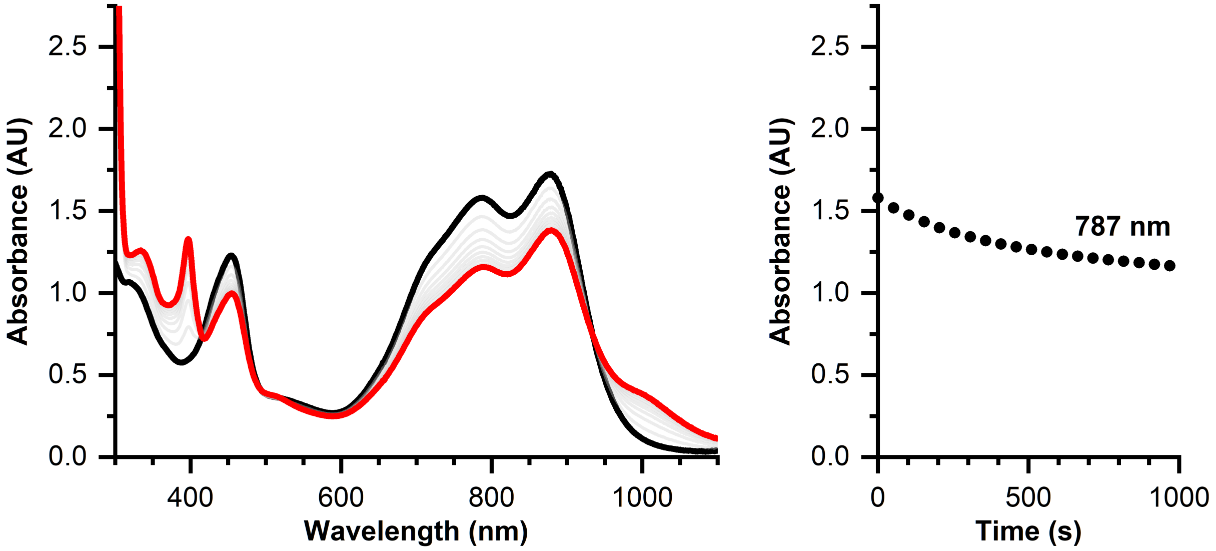

Figure S37. UV-vis spectra (left) and time course of the peak at 787 nm (right) in the reaction between $\mathbf{[^{bq}LCu(tmpda)]^+}$ (0.125 mM) and 4-MeO-DTBP (4.69 mM; 37.5 equiv) in DMF at 25 °C.

The BDFE of $\mathbf{[^{bq}LCu(tmpda)]^+}$ and pK_a of $\mathbf{[^{sq}LCu(tmpda)]}$ were approximated using the same analysis described above for $\mathbf{[^{bq}LCu(NEt_3)]^+}$ by analysing the equilibrium position of the reaction of $\mathbf{[^{bq}LCu(tmpda)]^+}$ with 4-MeO-DTBP in DMF at 25 °C. Equilibrium constants were determined using a "global" approach by monitoring the absorbance changes at three diagnostic wavelengths (455 nm, ε = 8800 $M^{-1}cm^{-1}$; 787 nm, ε = 11060 $M^{-1}cm^{-1}$; 875 nm, ε = 12200 $M^{-1}cm^{-1}$).[1] A summary of the calculated K_{eq} values is provided in **Table S6**.

Table S6. Equilibrium constants (K_{eq}) for the reaction of $\mathbf{[^{bq}LCu(tmpda)]^+}$ with 4-MeO-DTBP in DMF at multiple wavelengths.

Equiv. 4-MeO-DTBP	K_{eq} (455 nm)	K_{eq} (787 nm)	K_{eq} (875 nm)
**37.5**	0.00129	0.00296	0.00139

Table S7. Standard Gibbs free energy change (ΔG°) and bond dissociation free energy (BDFE) values for the reductive protonation of **[^{bq}LCu(tmpda)]$^+$** with 4-MeO-DTBP and the corresponding pK_a values for the protonation of **[^{sq}LCu(tmpda)]**.

Wavelength	ΔG° (kcal/mol)	BDFE (kcal/mol)	pK_a
**455**	3.9	68	7.2
**787**	3.4	68.5	7.5
**875**	3.9	68	7.2
**Overall Average***	**3.7 ± 0.6**	**68.2 ± 0.6**	**7.2 ± 0.4**

* Because the equilibrium between **[^{bq}LCu(tmpda)]$^+$** + 4-MeO-DTBP was measured at a single concentration of substrate, we report its uncertainty as the method-specific standard deviation determined for the related complex **[^{bq}LCu(NEt$_3$)]$^+$** measured under identical conditions.

Scheme S5. Square scheme for the reductive protonation of **[^{bq}LCu(tmpda)]$^+$**.

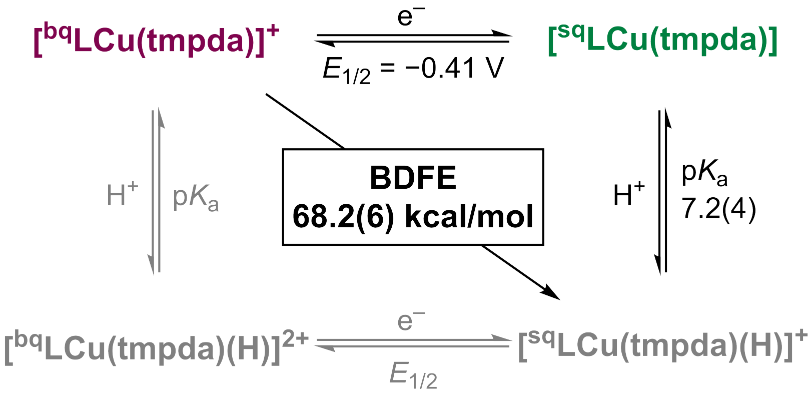

6. Kinetics

Observed rate constants (k_{obs}) were determined under pseudo-first order conditions by the method of initial rates. UV–vis absorption traces of the copper complex were monitored at the relevant λ_{max}, and the initial portion of each decay trace (first 5–15% of the total signal change) was fit to a linear function. The slope of this fit provided the initial rate for each experiment, which was converted to k_{obs} under the assumption of proportionality between absorbance and reactant concentration.

Representative fits of the absorption traces and linear fits of k_{obs} for the reactions of the $[LCu(NEt_3)]^n$ complexes with various substrates are shown on the following pages.

6.1. $[^{bq}LCu(NEt_3)]^+$

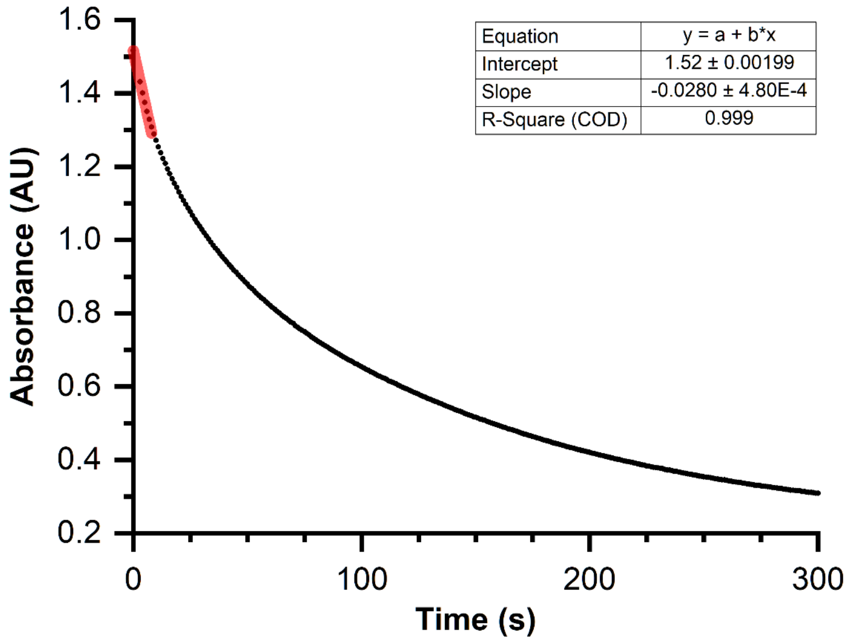

Figure S38. Representative fit of the initial decay of absorbance at λ_{max} = 782 nm vs. time for the reaction between $\mathbf{[^{bq}LCu(NEt_3)]^+}$ (0.125 mM) and TEMPOH (3.125 mM, 25 equiv) in DMF at 25 °C. The slope of the fit corresponds to the initial rate of the reaction.

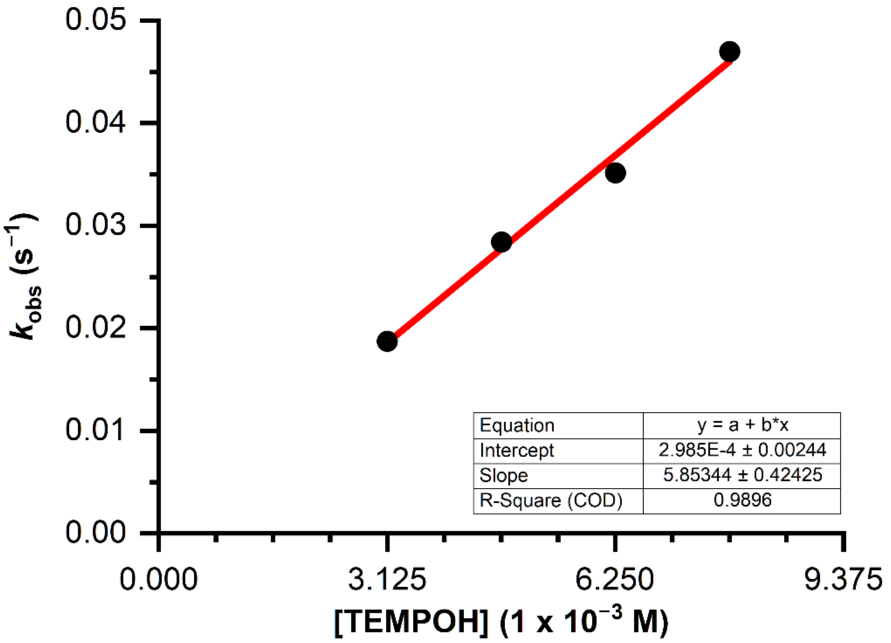

Figure S39. Linear fit of k_{obs} versus concentration of TEMPOH (3.125–7.8125 mM; 25–62.5 equiv) for the reactions with $\mathbf{[^{bq}LCu(NEt_3)]^+}$ (0.125 mM) in DMF at 25 °C. The slope of the fit corresponds to the second-order rate constant (k_2).

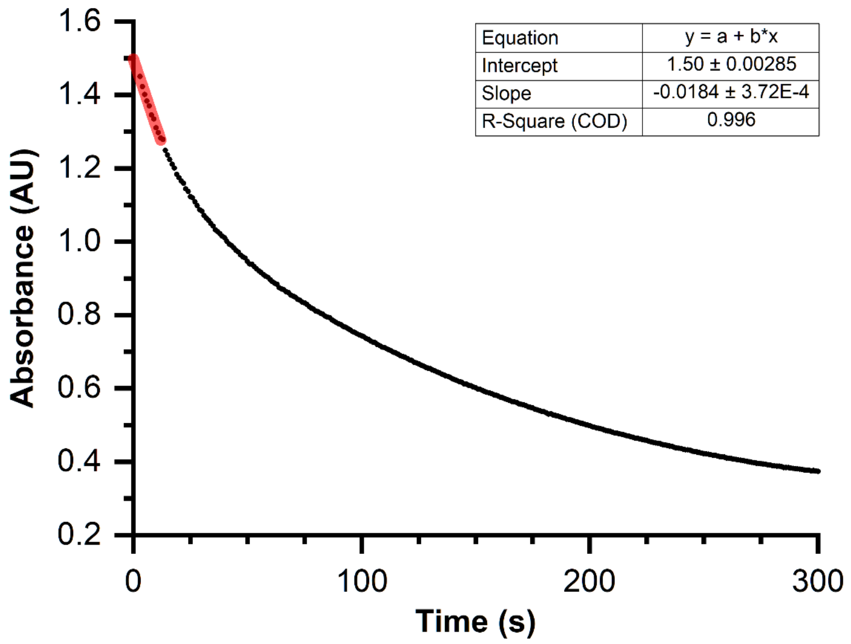

Figure S40. Representative fit of the initial decay of the peak at λ_{max} = 782 nm for the reaction between **[^{bq}LCu(NEt$_3$)]$^+$** (0.125 mM) and TEMPOD (3.125 mM, 25 equiv) in DMF at 25 °C. The slope of the fit corresponds to the initial rate of the reaction.

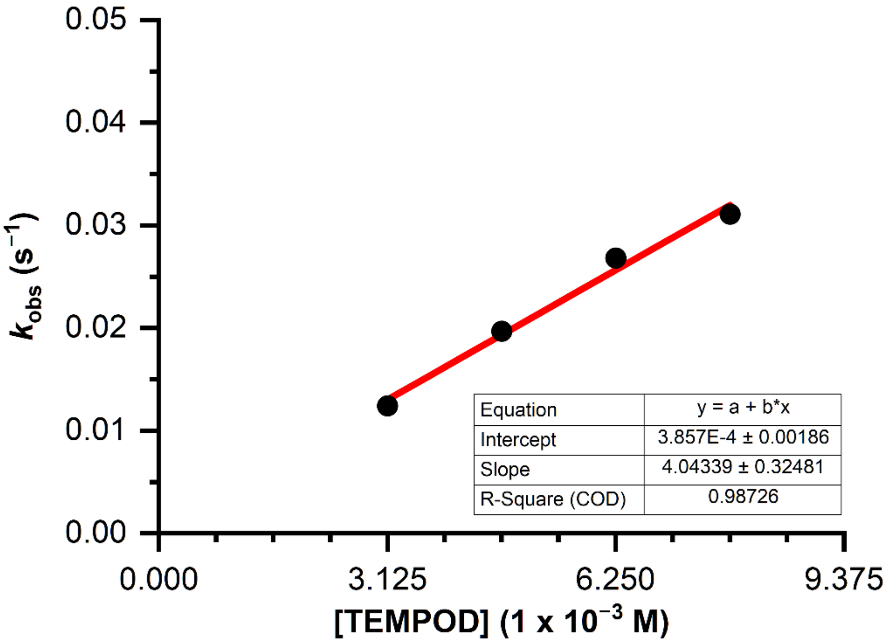

Figure S41. Linear fit of k_{obs} versus concentration of TEMPOD (3.125–7.8125 mM; 25–62.5 equiv) for the reaction of **[^{bq}LCu(NEt$_3$)]$^+$** (0.125 mM) in DMF at 25 °C. The slope of the fit corresponds to the second-order rate constant (k_2).

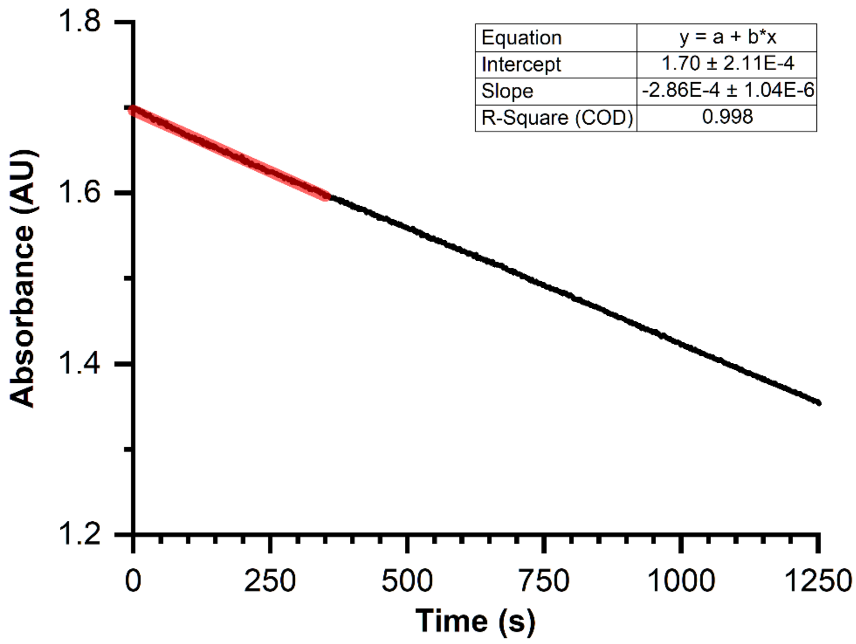

Figure S42. Representative fit of the initial decay of the peak at λ_{max} = 782 nm for the reaction between **[^{bq}LCu(NEt$_3$)]$^+$** (0.125 mM) and xanthene (18.75 mM, 150 equiv) in DMF at 25 °C. The slope of the fit corresponds to the initial rate of the reaction.

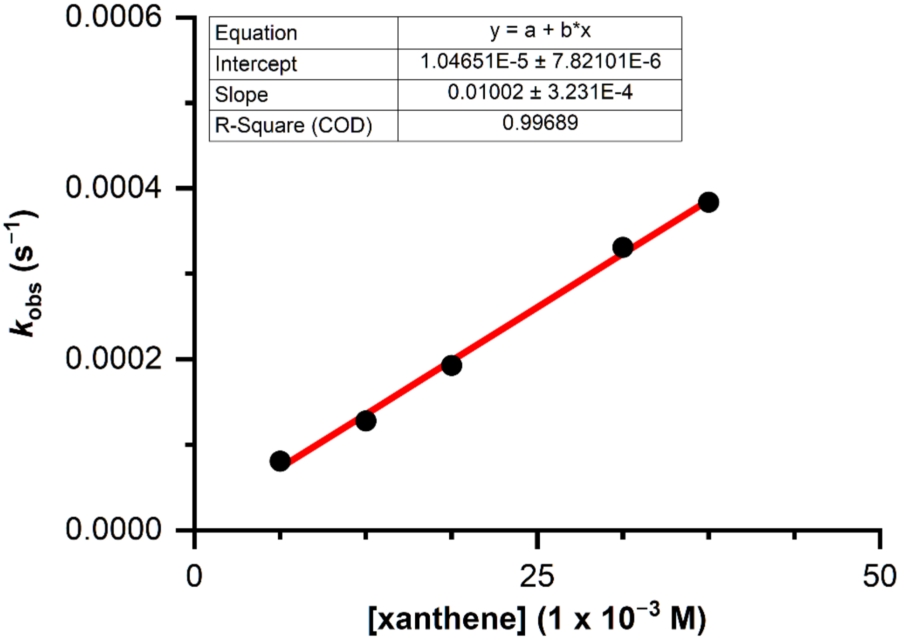

Figure S43. Linear fit of k_{obs} versus concentration of xanthene (6.25–37.5 mM; 50–300 equiv) for the reaction of **[^{bq}LCu(NEt$_3$)]$^+$** (0.125 mM) in DMF at 25 °C. The slope of the fit corresponds to the second-order rate constant (k_2).

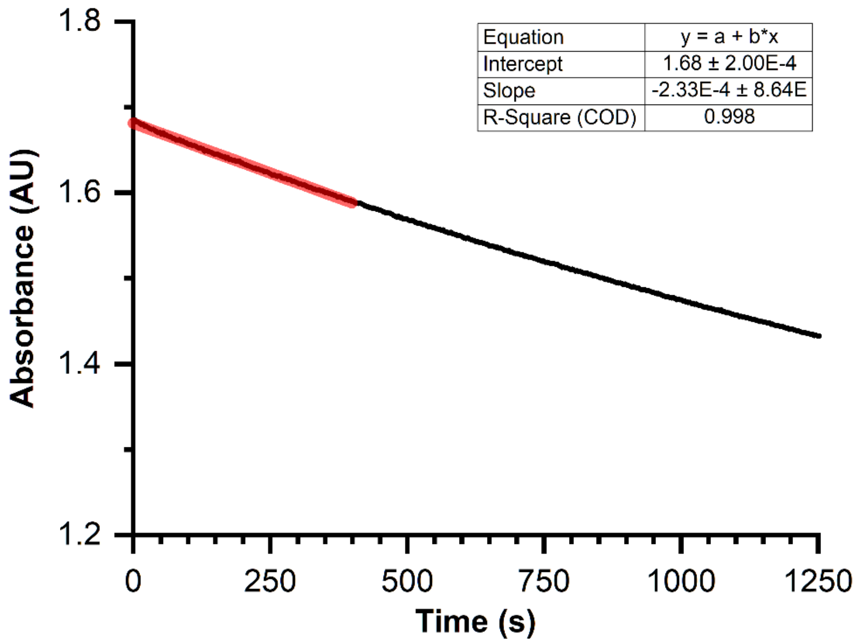

Figure S44. Representative fit of the initial decay of the peak at λ_{max} = 782 nm for the reaction between **[^{bq}LCu(NEt$_3$)]$^+$** (0.125 mM) and xanthene-d_2 (18.75 mM, 150 equiv) in DMF at 25 °C. The slope of the fit corresponds to the initial rate of the reaction.

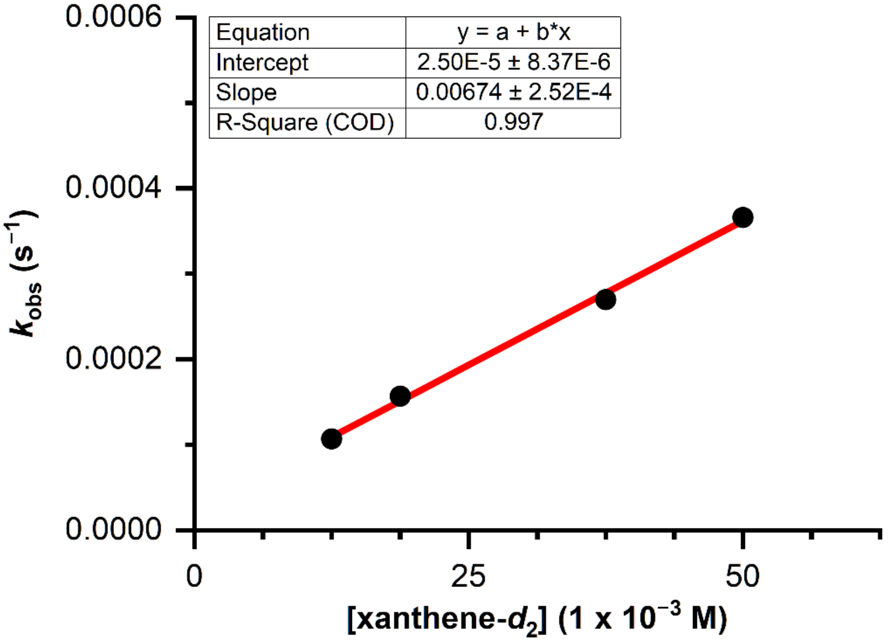

Figure S45. Linear fit of k_{obs} versus concentration of xanthene-d_2 (6.25–50 mM; 50–400 equiv) for the reaction of **[^{bq}LCu(NEt$_3$)]$^+$** (0.125 mM) in DMF at 25 °C. The slope of the fit corresponds to the second-order rate constant (k_2).

6.2. [^{sq}LCu(NEt$_3$)]

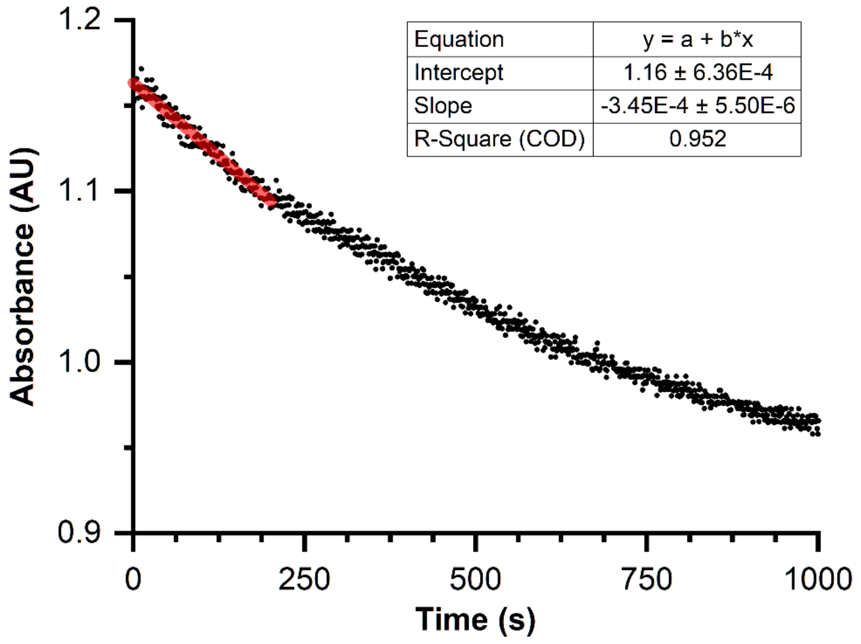

Figure S46. Representative fit of the initial decay of the peak at λ_{max} = 1020 nm for the reaction between **[^{sq}LCu(NEt$_3$)]** (0.125 mM) and TEMPOH (6.25 mM, 50 equiv) in DMF at 25 °C. The slope of the fit corresponds to the initial rate of the reaction.

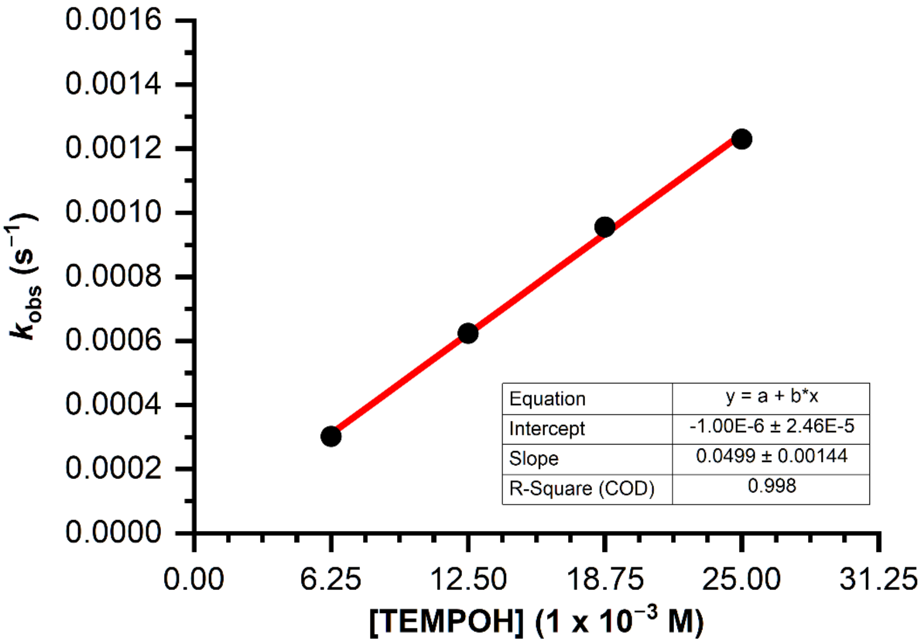

Figure S47. Linear fit of k_{obs} versus concentration of TEMPOH (6.25–25 mM; 50–200 equiv) for the reaction of **[^{sq}LCu(NEt$_3$)]** (0.125 mM) in DMF at 25 °C. The slope of the fit corresponds to the second-order rate constant (k_2).

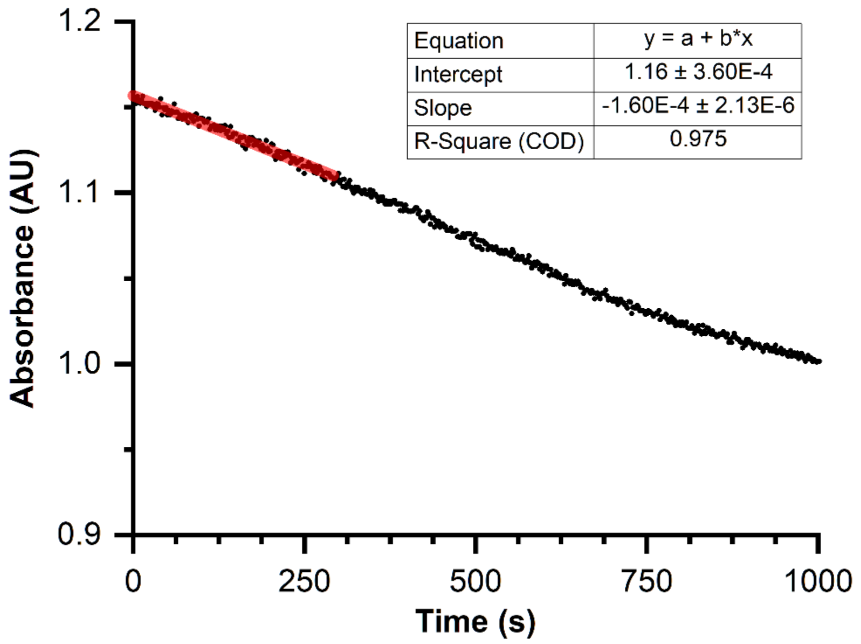

Figure S48. Representative fit of the initial decay of the peak at λ_{max} = 1020 nm for the reaction between **[^{sq}LCu(NEt$_3$)]** (0.125 mM) and TEMPOD (6.25 mM, 50 equiv) in DMF at 25 °C. The slope of the fit corresponds to the initial rate of the reaction.

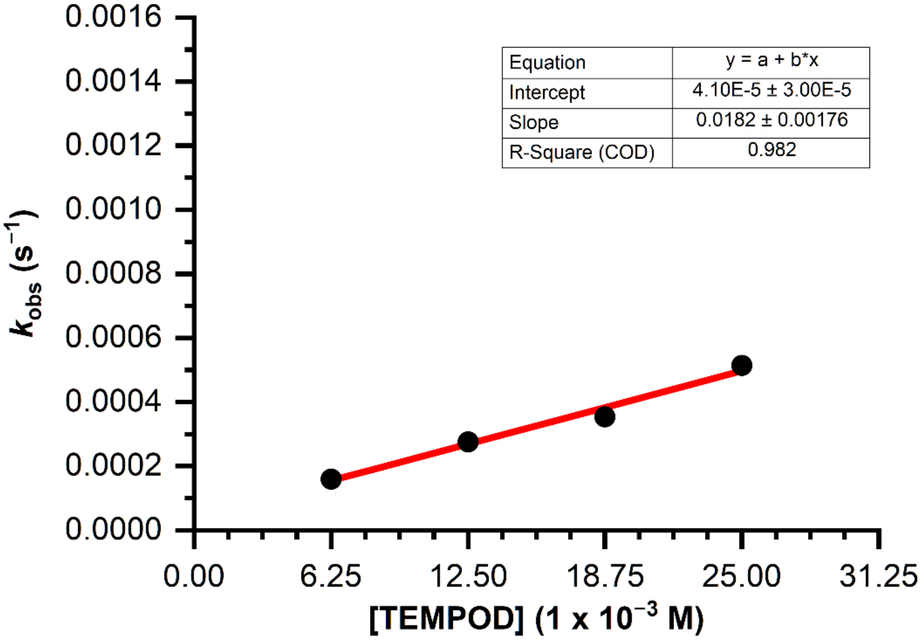

Figure S49. Linear fit of k_{obs} versus concentration of TEMPOD (6.25–25 mM; 50–200 equiv) for the reaction of **[^{sq}LCu(NEt$_3$)]** (0.125 mM) in DMF at 25 °C. The slope of the fit corresponds to the second-order rate constant (k_2).

7. Reaction of $[^{bq}LCu(NEt_3)]^+$ with xanthene monitored by ^1H NMR

^1H NMR spectroscopy was used to identify and quantify the oxidation products formed in the reaction between **$[^{bq}LCu(NEt_3)]^+$** and xanthene. The products were assigned as xanthydrol, xanthone, and bixanthene, based on comparison with authentic samples (xanthydrol), spike-sample experiments (xanthone), and literature data (bixanthene). Blank experiments using the starting xanthene confirmed the presence of a 2% xanthone impurity, which was accounted for when determining the yield of xanthone.

The ^1H NMR spectrum of bixanthene is unreported in DMF-d_7, however, it has been reported in DMSO-d_6.[17] Based on the similar polarity and hydrogen-bonding properties of DMF and DMSO, we assume that the chemical shifts in DMF-d_7 are comparable to those reported in DMSO-d_6.

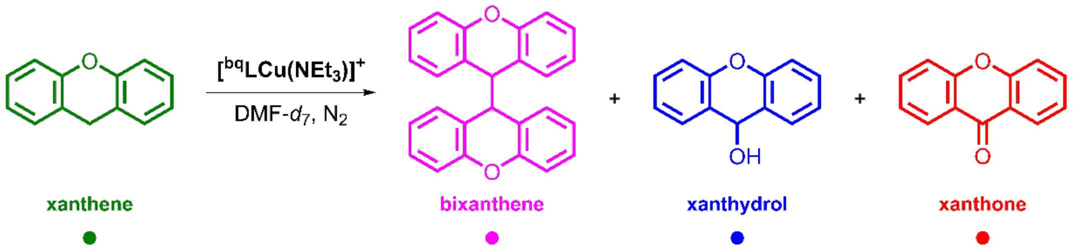

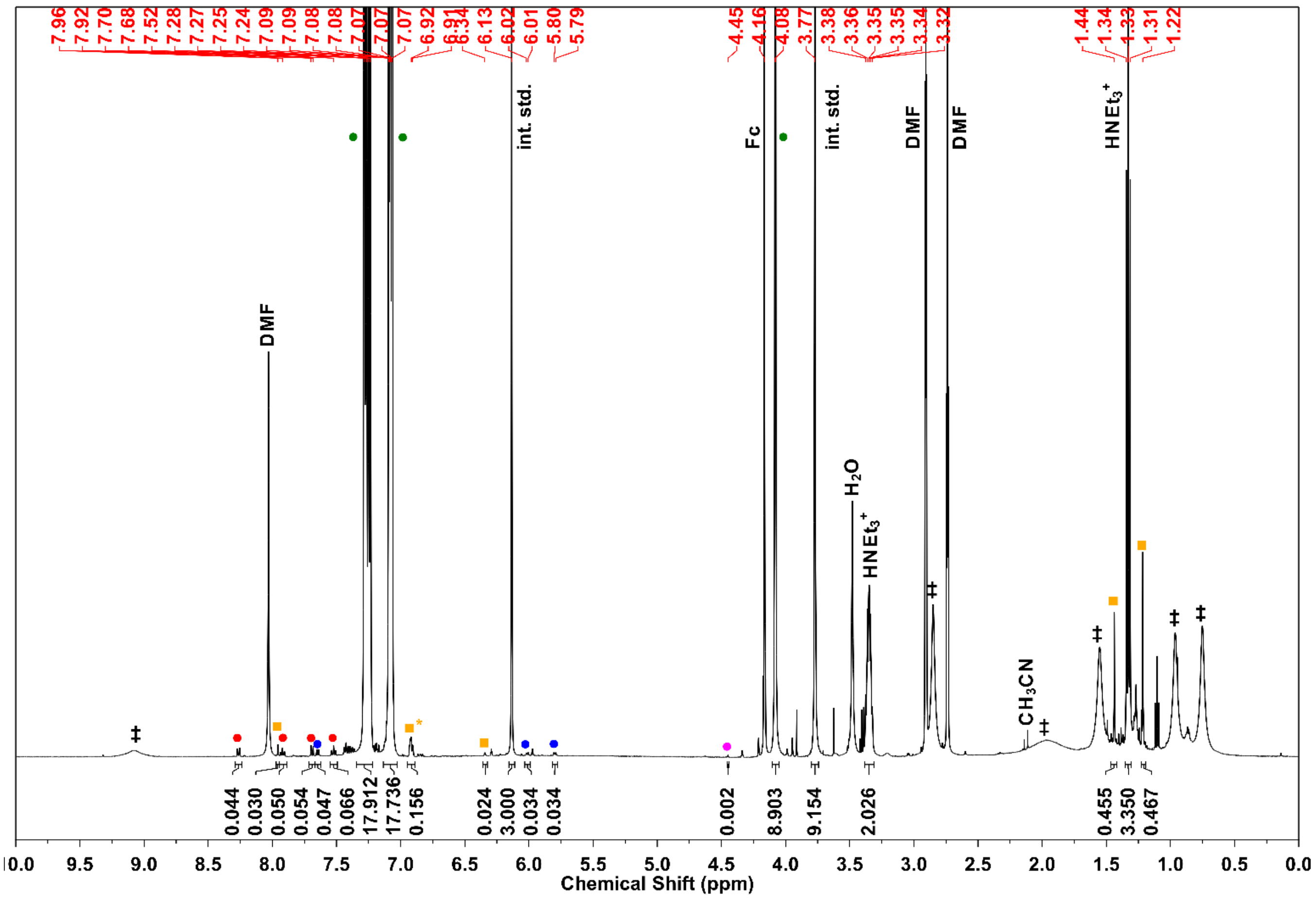

Figure S50. ^{1}H NMR spectrum of the reaction between $[^{bq}LCu(NEt_3)]^+$ (8.0 mM) with xanthene (80 mM, 10 equiv) in DMF-d_7. Peaks labeled "●" correspond to xanthene; "●" to xanthone; "●" to xanthydrol; "●" to bixanthene, and "■" to the cuprous species $[(^{cat}LH_2)Cu^I(DMF)]$. The broad signals labeled "‡" are assigned to paramagnetic Cu^{II} species, and the peak labeled "**Fc**" corresponds to ferrocene. *Note:* The aromatic $[(^{cat}LH_2)Cu^I(DMF)]$ peaks at 6.92 ppm ("■*") overlap with impurities in the xanthene.

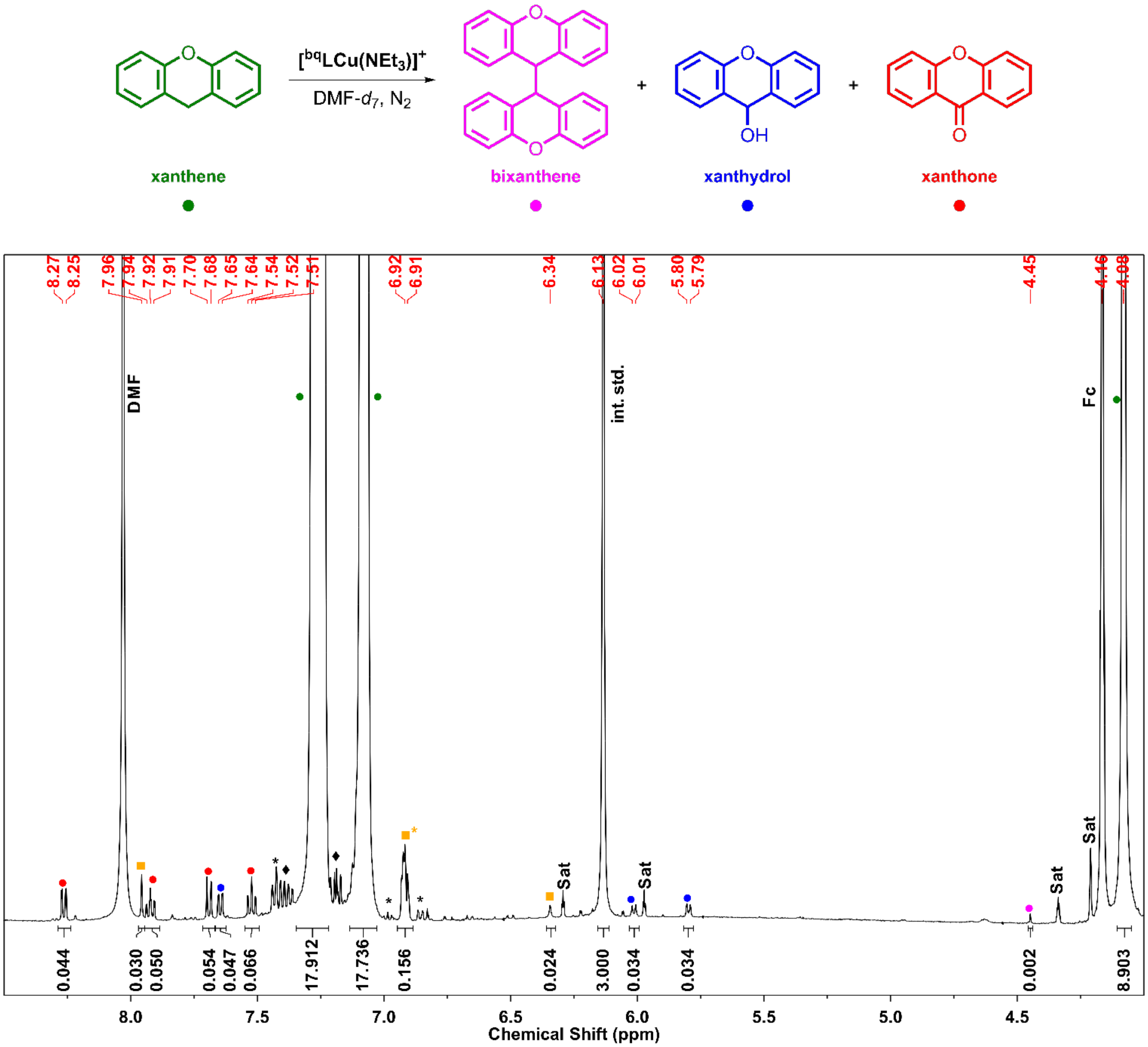

Figure S51. Expanded ^{1}H NMR spectrum (8.4–4.0 ppm) of the reaction between **[^{bq}LCu(NEt$_3$)]$^+$** (8.0 mM) with xanthene (80 mM, 10 equiv) in DMF-*d*$_7$. Peaks labeled "●" correspond to xanthene; "●" to xanthone; "●" to xanthydrol; "●" to bixanthene, and "■" to the cuprous species **[(^{cat}LH$_2$)Cu^I(DMF)]**. Signals labeled "*" are assigned to impurities in xanthene, and those labeled "**Sat**" indicate satellite peaks. Peaks labeled "♦" correspond to unidentified products. The peak labeled "**Fc**" corresponds to ferrocene. *Note:* For clarity, the large aromatic xanthene signals in the 7.0–7.3 ppm region were not peak-picked. The aromatic **[(^{cat}LH$_2$)Cu^I(DMF)]** resonance at 6.92 ppm ("■*") overlaps with impurities in the xanthene.

Table S8. Equivalents, yields, and stoichiometry of the xanthene oxidation products formed in the reaction between **[^{bq}LCu(NEt$_3$)]$^+$** and xanthene as determined by ^{1}H NMR spectroscopy.

	Equiv relative to [Cu]$_0$	Yield (%)*	Stoichiometry (nH$^+$/ne$^-$)
bixanthene	0.00150	0.3	2
xanthydrol	0.0456	9	2
xanthone	0.0179	7	4

Scheme S6. Reaction scheme and yields of xanthene oxidation products for the reaction between **[^{bq}LCu(NEt$_3$)]$^+$** and xanthene. Yields are relative to [Cu] and are normalized to reflect the electron stoichiometry for the oxidation of xanthene to the corresponding product.

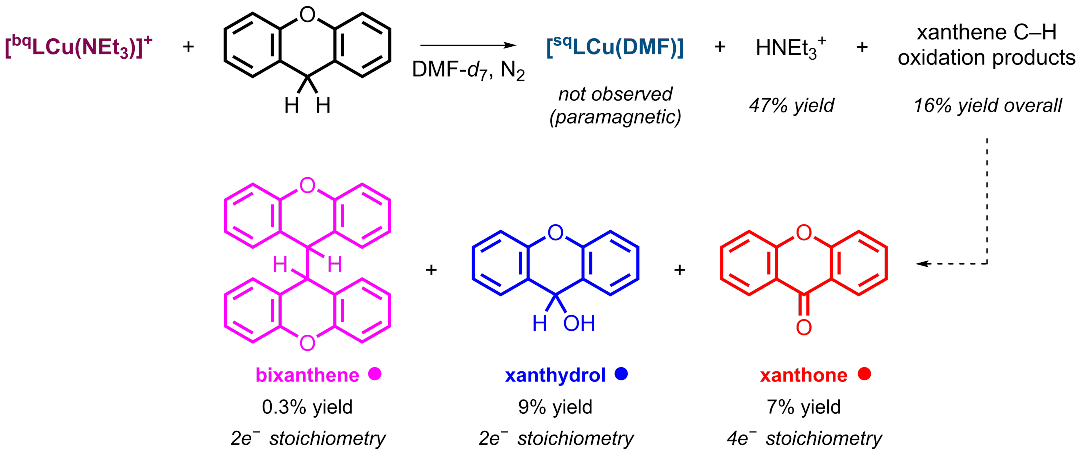

Note: As shown in the NMR spectra above, a small amount of **[(^{cat}LH$_2$)Cu^I(DMF)]$^-$** is observed. This may arise from protonation of **[^{sq}LCu(DMF)]** followed by disproportionation under the reaction conditions. Alternatively, **[^{sq}LCu(DMF)]** may potentially react with xanthydrol, as the related complex **[^{sq}LCu(NEt$_3$)]** has been shown to be capable of dehydrogenating benzylic alcohols.[8] These interpretations are tentative and require further investigation.

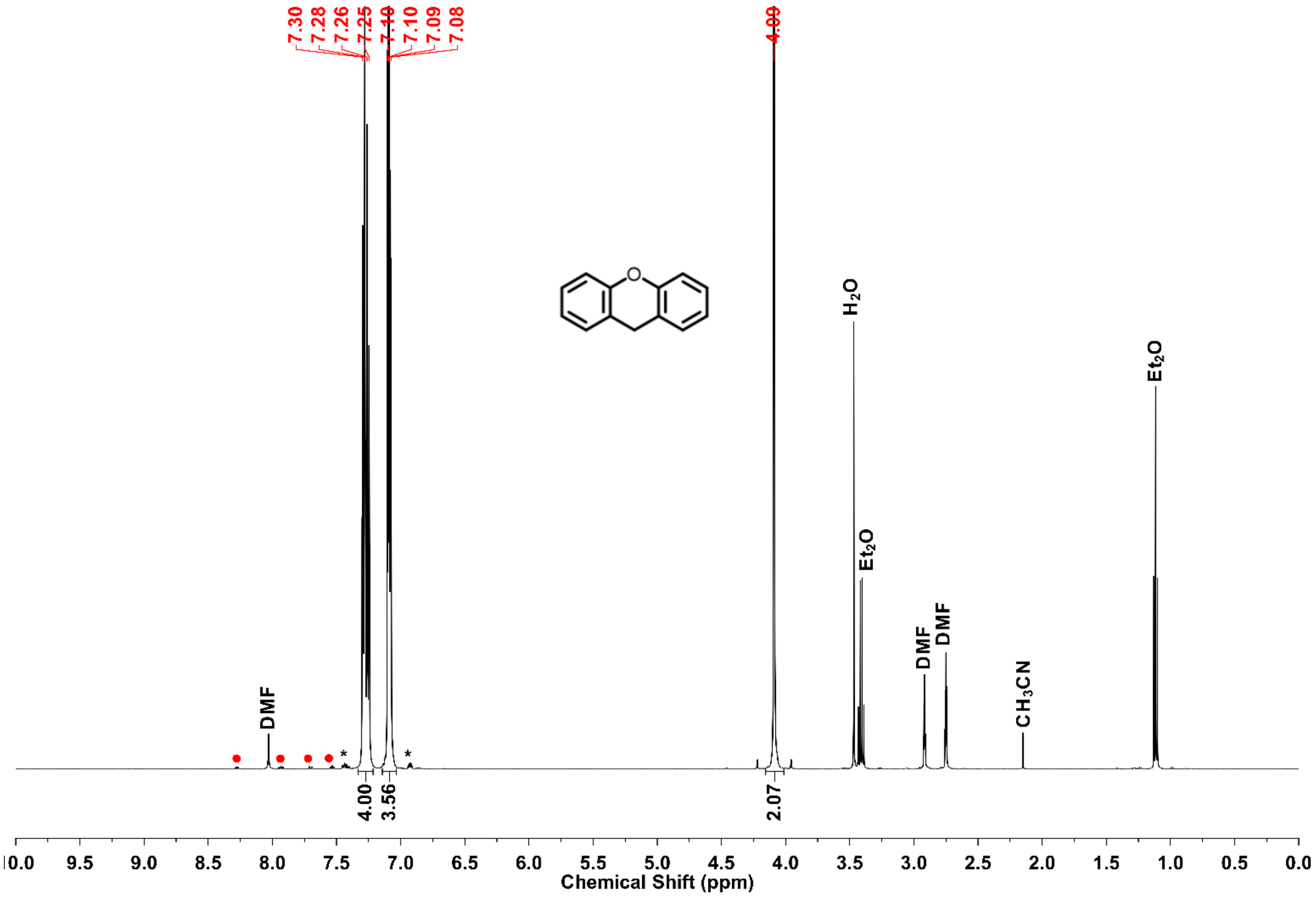

Figure S52. ^{1}H NMR spectrum of xanthene in DMF-d_7. Peaks labeled "●" correspond xanthone and those labeled "*" are unidentified impurities in xanthene. Xanthone is present in 2% relative to xanthene in the starting material. Solvent impurities in the DMF-d_7 are labeled accordingly.

xanthene: ^{1}H NMR (500 MHz, DMF-d_7) δ [ppm]: 7.27 (m, 4H), 7.09 (m, 4H), 4.09 (s, 2H).

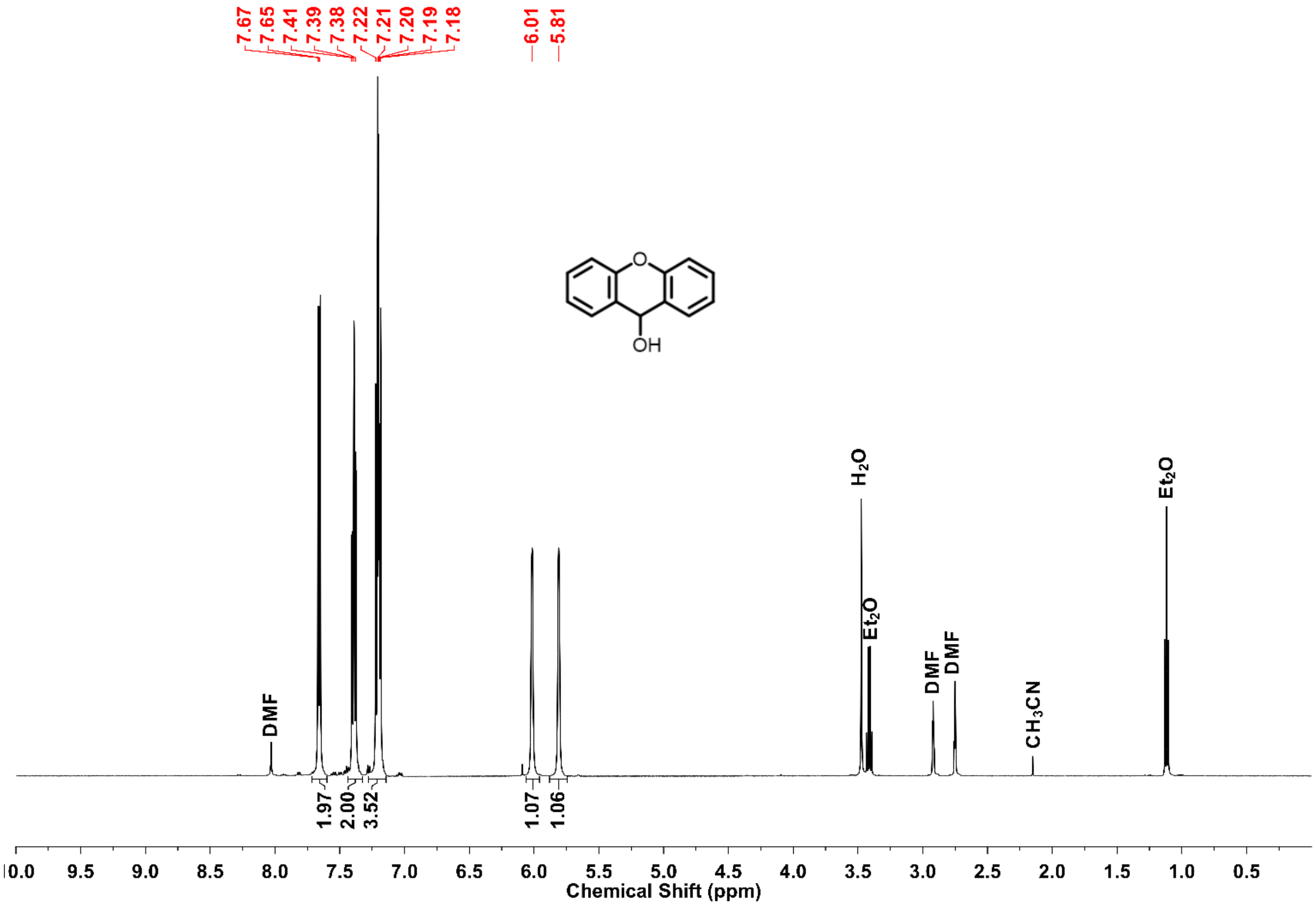

Figure S53. ^{1}H NMR spectrum of xanthydrol in DMF-d_7. Solvent impurities in the DMF-d_7 are labeled accordingly.

xanthydrol: ^{1}H NMR (500 MHz, DMF-d_7) δ [ppm]: 7.66 (d, J = 6.2 Hz, 2H), 7.39 (t, J = 7.7 Hz, 2H), 7.26 – 7.14 (m, 4H), 6.01 (s, 1H), 5.81 (s, 1H).

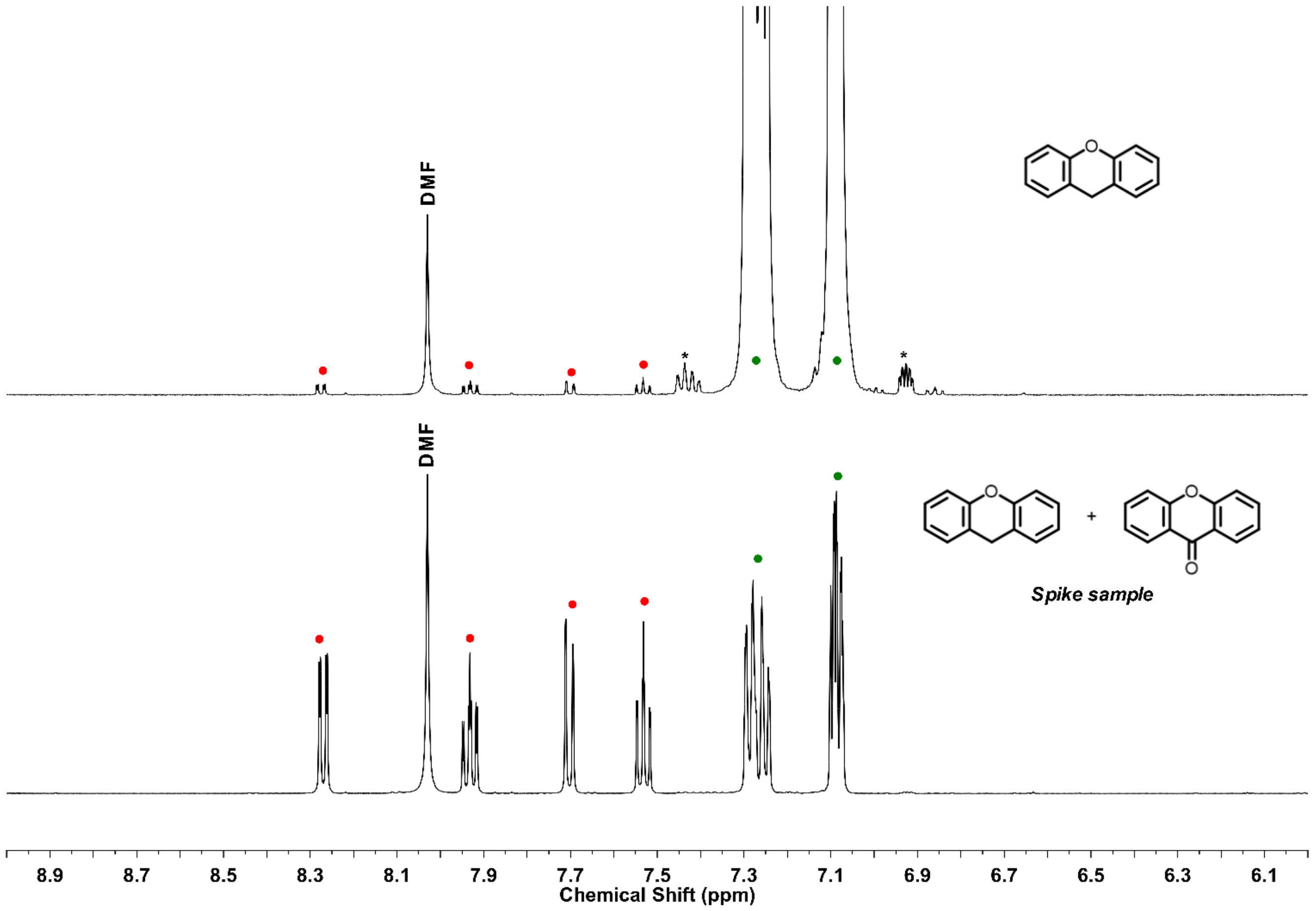

Figure 54. ^{1}H NMR spectra of xanthene (top) and xanthene spiked with xanthone (bottom) in DMF-d_7. Peaks labeled with "*" are unidentified impurities in xanthene.

xanthone: ^{1}H NMR (500 MHz, DMF-d_7) δ [ppm]: 8.27 (dd, J = 7.9, 1.6 Hz, 2H), 7.93 (ddd, J = 8.7, 7.1, 1.7 Hz, 2H), 7.70 (dd, J = 8.4, 0.6 Hz, 2H), 7.53 (t, J = 7.5 Hz, 2H).

8. References

(1) Hebert, D. D.; Puri, A.; Ye, D.; McAninch, A.; Chisholm, A.; Siegler, M. A.; Swart, M.; Garcia-Bosch, I. Synthesis and Characterization of Copper Complexes Featuring a Redox-Active ONO Ligand in Three Molecular Oxidation States. *Inorg. Chem.* **2025**, *64* (22), 11204–11218. https://doi.org/10.1021/acs.inorgchem.5c01578.

(2) De Vries, J. G.; Kellogg, R. M. Reduction of Aldehydes and Ketones by Sodium Dithionite. *J. Org. Chem.* **1980**, *45* (21), 4126–4129. https://doi.org/10.1021/jo01309a011.

(3) Mader, E. A.; Davidson, E. R.; Mayer, J. M. Large Ground-State Entropy Changes for Hydrogen Atom Transfer Reactions of Iron Complexes. *J. Am. Chem. Soc.* **2007**, *129* (16), 5153–5166. https://doi.org/10.1021/ja0686918.

(4) Barras, J.-P.; Davies, S. G.; Metzler, M. R.; Edwards, A. J.; Humphreys, V. M.; Prout, K. Synthesis and Reactivity of the Pentamethylcyclopentadienyl Iron Acetyl Complex [(H5-C5Me5)Fe(CO)(PPh3)COMe]. *J. Organomet. Chem.* **1993**, *461* (1–2), 157–165. https://doi.org/10.1016/0022-328x(93)83287-6.

(5) Kang, Y.; Chen, H.; Jeong, Y. J.; Lai, W.; Bae, E. H.; Shaik, S.; Nam, W. Enhanced Reactivities of Iron(IV)-Oxo Porphyrin Pi-Cation Radicals in Oxygenation Reactions by Electron-Donating Axial Ligands. *Chem. Eur. J.* **2009**, *15* (39), 10039–10046. https://doi.org/10.1002/chem.200901238.

(6) Favier, I.; Duñach, E. New Protic Salts of Aprotic Polar Solvents. *Tetrahedron Lett.* **2004**, *45* (17), 3393–3395. https://doi.org/10.1016/j.tetlet.2004.03.025.

(7) Zarkesh, R. A.; Ziller, J. W.; Heyduk, A. F. Four-Electron Oxidative Formation of Aryl Diazenes Using a Tantalum Redox-Active Ligand Complex. *Angew. Chem. Int. Ed Engl.* **2008**, *47* (25), 4715–4718. https://doi.org/10.1002/anie.200800812.

(8) Chaudhuri, P.; Hess, M.; Weyhermüller, T.; Wieghardt, K. Aerobic Oxidation of Primary Alcohols by a New Mononuclear Cu(II) -Radical Catalyst. *Angew. Chem. Int. Ed Engl.* **1999**, *38* (8), 1095–1098. https://doi.org/10.1002/(SICI)1521-3773(19990419)38:8<1095::AID-ANIE1095>3.0.CO;2-I.

(9) Hebert, D. D.; Cohen, E. P. *Uv_pro v0.6.6*; Zenodo, 2025. https://doi.org/10.5281/ZENODO.14963404.

(10) Wu, T.; Puri, A.; Qiu, Y. L.; Ye, D.; Sarma, R.; Wang, Y.; Kowalewski, T.; Siegler, M. A.; Swart, M.; Garcia-Bosch, I. Tuning the Thermochemistry and Reactivity of a Series of Cu-Based 4H+/4e- Electron-Coupled-Proton Buffers. *Inorg. Chem.* **2024**, *63* (20), 9014–9025. https://doi.org/10.1021/acs.inorgchem.4c00835.

(11) Agarwal, R. G.; Coste, S. C.; Groff, B. D.; Heuer, A. M.; Noh, H.; Parada, G. A.; Wise, C. F.; Nichols, E. M.; Warren, J. J.; Mayer, J. M. Free Energies of Proton-Coupled Electron Transfer Reagents and Their Applications. *Chem. Rev.* **2022**, *122* (1), 1–49. https://doi.org/10.1021/acs.chemrev.1c00521.

(12) Wu, T.; Rajabimoghadam, K.; Puri, A.; Hebert, D. D.; Qiu, Y. L.; Eichelberger, S.; Siegler, M. A.; Swart, M.; Hendrich, M. P.; Garcia-Bosch, I. A 4H+/4e- Electron-Coupled-Proton Buffer Based on a

Mononuclear Cu Complex. *J. Am. Chem. Soc.* **2022**, *144* (37), 16905–16915. https://doi.org/10.1021/jacs.2c05454.

(13) Wu, T.; MacMillan, S. N.; Rajabimoghadam, K.; Siegler, M. A.; Lancaster, K. M.; Garcia-Bosch, I. Structure, Spectroscopy, and Reactivity of a Mononuclear Copper Hydroxide Complex in Three Molecular Oxidation States. *J. Am. Chem. Soc.* **2020**, *142* (28), 12265–12276. https://doi.org/10.1021/jacs.0c03867.

(14) Hebert, D.; Cohen, E. P. *Uv_pro: V0.8.0*; Zenodo, 2025. https://doi.org/10.5281/ZENODO.16757699.

(15) Wise, C. F.; Agarwal, R. G.; Mayer, J. M. Determining Proton-Coupled Standard Potentials and X-H Bond Dissociation Free Energies in Nonaqueous Solvents Using Open-Circuit Potential Measurements. *J. Am. Chem. Soc.* **2020**, *142* (24), 10681–10691. https://doi.org/10.1021/jacs.0c01032.

(16) Kütt, A.; Tshepelevitsh, S.; Saame, J.; Lõkov, M.; Kaljurand, I.; Selberg, S.; Leito, I. Strengths of Acids in Acetonitrile. *European J. Org. Chem.* **2021**, *2021* (9), 1407–1419. https://doi.org/10.1002/ejoc.202001649.

(17) Pan, H.-R.; Wu, J.; Tsai, C.-M.; Liao, P.-J.; Hsu, H.-F. Basicity-Controlled C-H Bond Activation by a Structurally Characterized Ni(III)-Hydroxo Complex. *J. Am. Chem. Soc.* **2025**, *147* (31), 27855–27861. https://doi.org/10.1021/jacs.5c06941.

9. Appendix

9.1. Abbreviations

- DMF•TfOH (N,N-dimethylformamidinium triflate)
- tmpda (N,N,N′,N′-tetramethylpropane-1,3-diamine)
- Fc (ferrocene)
